# Thianthrenium Chemistry for Identification of Protein–Protein Interactions in Cells

**DOI:** 10.1021/jacs.5c16665

**Published:** 2025-12-01

**Authors:** Kostiantyn Bohdan, Philipp Hartmann, Sven Müller, Dario Marchionni, Christian Preisinger, Julia Beatrice Jacobs, Lara Vogelsang, Marie Sophie Sterling, Karl-Josef Dietz, Tobias Ritter

**Affiliations:** † 28314 Max-Planck-Institut für Kohlenforschung, Kaiser-Wilhelm-Platz 1, 45470 Mülheim an der Ruhr, Germany; ¶ Institute of Organic Chemistry, RWTH Aachen University, Landoltweg 1, 52074 Aachen, Germany; § Proteomics Facility, Interdisciplinary Centre for Clinical Research (IZKF), RWTH Aachen University, 52074 Aachen, Germany; ∥ Biochemistry and Physiology of Plants, Faculty of Biology, Bielefeld University, Universitätsstraße 25, 33615 Bielefeld, Germany

## Abstract

We developed a synthetically readily accessible dicationic thianthrenium reagent for reliable identification of protein–protein interactions (PPIs) *in cellulo*. Cysteine (Cys)-selective Michael addition to *in situ* generated alkenyl thianthrenium salts leads to formation of reactive episulfonium intermediates on protein surfaces that covalently trap all nucleophilic amino acids of interacting proteins. Short and chemically stable ethylene linkages are introduced in a single step and thereby provide short distance restraints for reliable modeling of PPI interfaces. The fundamental advance described in this work is the development of a reagent for identification of low-abundant interprotein cross-links via episulfonium chemistry enabled by the introduction of an alkyne tag for downstream enrichment of cross-linked products without an extension of the linkage length between the two cross-linked amino acids. Based on the fast single-step cross-linking, we show that our method allows for more accurate structure prediction of reported PPIs compared to data produced by commonly used bifunctional cross-linking reagents. We further demonstrate that our approach enables identification of previously undocumented PPIs in human cancer cells by targeting native Cys residues and that the obtained short distance restraints provide valuable information for modeling of PPI surfaces.

## Graphical abstract

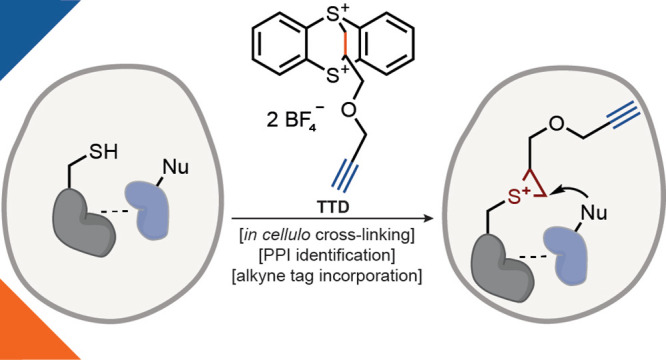

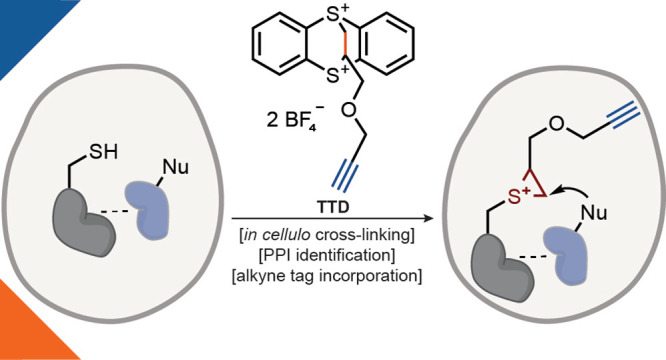

## Introduction

Transient and permanent protein–protein interactions govern fundamental intracellular processes such as signal transduction and regulation of cellular metabolism.^1,2^ Dysregulation or non-native protein interactions can cause diseases and, therefore, PPI identification can provide new drug targets based on the modeled interaction surfaces.^3,4^ Among the methods that have been developed to detect PPIs in the native cellular environment,^5^ chemical cross-linking stands out because weak and transient interactions between proteins can be captured via formation of stable covalent linkages. The identity of the cross-linked peptides and the derived distance restraints provide insights into the topology of the detected PPIs.^6^ Most chemical cross-linking reagents are bifunctional electrophiles^7^ that target surface-exposed lysine residues^8^ (Figure 1a). The reagents can carry bioorthogonal groups for enrichment of cross-linked species but enrichment tag incorporation requires linker elongation^9^ or bulky functionalities that impair cellular uptake.^10^ Extended linker length and the long half-life of generated intermediates can lead to capturing of false-positive PPIs or unreliable PPI structure modeling.^7^ Generation of carbene intermediates is an alternative strategy that relies on fast formation of short interprotein cross-links upon irradiation (Figure 1b). Photoreactive carbene precursors like diazirines can be introduced proteome-wide by metabolic labeling or via heterobifunctional cross-linkers.^11–13^ However, both methods rely on UV light for carbene generation. Cross-linking with heterobifunctional reagents occurs in two separate steps, whereas metabolic diazirine incorporation requires optimization for each cell type^14,15^ and does not permit affinity enrichment of low-abundance cross-linked products. Fractionation enhances the detection sensitivity in complex samples but remains less effective than affinity-based enrichment.^16,17^ No current method enables proteome-wide formation of short-lived intermediates on native residues for single-step short-distance cross-linking and affinity tag incorporation for downstream enrichment. Herein, we report a new thianthrenium reagent that enables formation of short and stable cross-links along with incorporation of an enrichment tag that does not elongate the linker and allows for efficient identification of new PPIs in cellular environments. The salient features of the method include single-step cross-linking between native residues, enrichment of cross-linked products for enhanced sensitivity, and short distance restraints for reliable PPI structure modeling.

**Figure 1:**
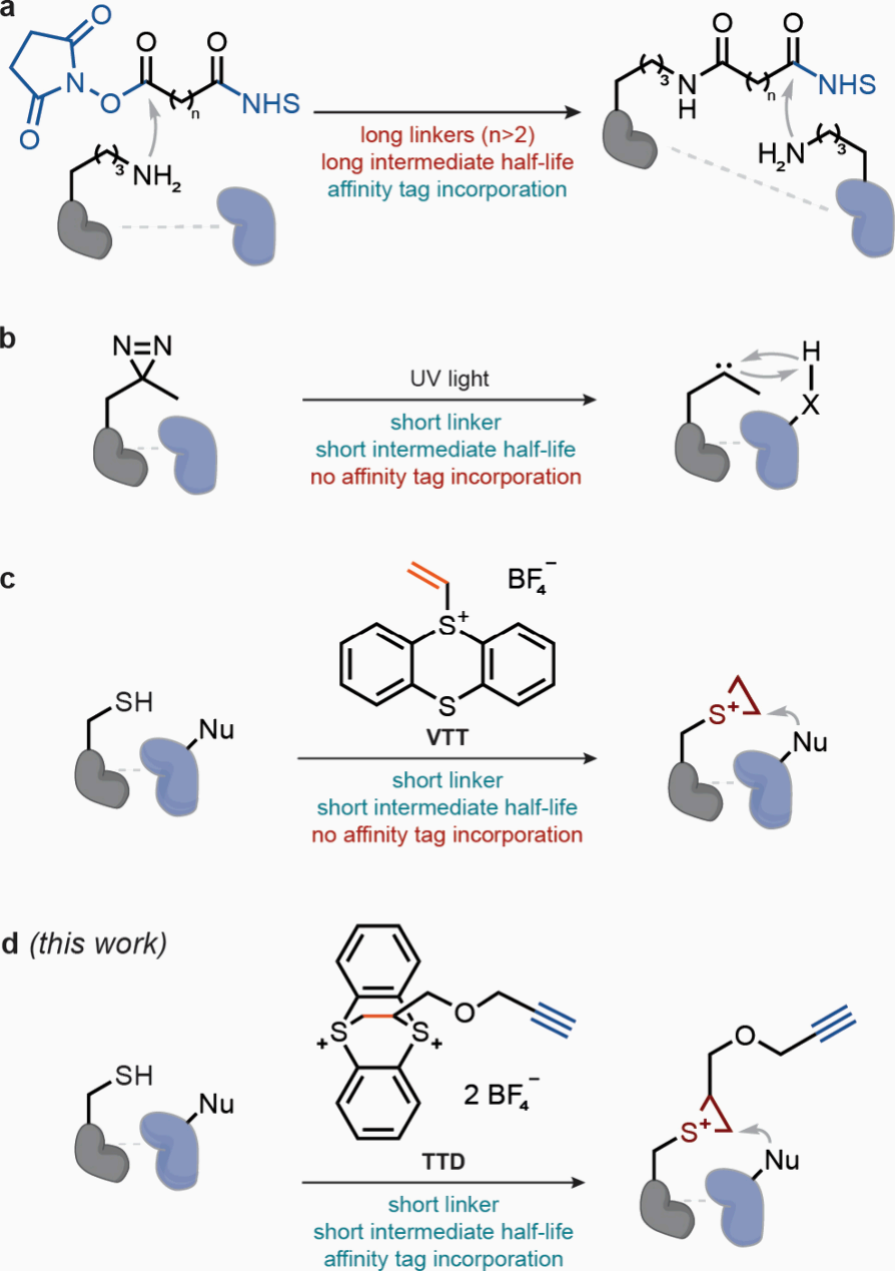
Cross-linking strategies for PPI identification. (a) Cross-linking via two subsequent reactions of homobifunctional lysine-selective reagents with lysine residues. NHS, *N*-hydroxysuccinimide. (b) Cross-linking via incorporation of diazirine-containing unnatural amino acids and reactive carbene generation upon UV light exposure. (c) **VTT**-mediated cross-linking via episulfonium formation. (d) Episulfonium-mediated cross-linking via enrichable thianthrenium reagent for identification of low-abundant interprotein cross-links (this work). **TTD**, thianthrenium dication reagent developed in this work.

We previously established that vinylthianthrenium tetrafluoroborate (**VTT**) enables Cys umpolung and short cross-link formation for fast protein cross-linking *in vitro* ^18^ and *in cellulo*.^19^ Rate-determining Michael addition to Cys-reactive **VTT** leads to fast displacement of thianthrene as a leaving group and formation of electrophilic episulfonium intermediates on proteins, which are rapidly trapped by neighboring nucleophiles to form stable products with introduced moieties close to the protein surfaces. In contrast to bifunctional electrophilic reagents commonly used for protein cross-linking, **VTT**-mediated cross-link formation occurs in a single step due to the high reactivity of the generated episulfonium intermediate and unlike diazirine-based cross-linkers does not require activation via UV light to generate reactive intermediates in a separate step. However, **VTT** cannot be used in cross-linking studies that require subsequent enrichment of cross-linked products because obtained products are linked via a simple ethylene linkage that does not permit further chemical manipulation (Figure 1c). We hypothesized that a reagent that forms substituted episulfonium intermediates upon intramolecular displacement of thianthrene could enable single-step interprotein cross-linking without an increase in distance restraints generated for subsequent PPI modeling studies. We proposed that a short linker can be used to connect the bioorthogonal alkyne tag with the reactive thianthrenium moiety and ensure a minimal effect on the reagent size and polarity to preserve potential cell permeability and therefore efficient intracellular labeling by the reagent. Our hypothesis was that the high reactivity of the episulfonium intermediates combined with enrichment of cross-linked products would enable identification of low-abundant interprotein cross-links from short-distance PPIs that could not be detected using the established lysine-targeting cross-linking methods, as these rely on the presence of two lysine residues within the interaction interface (Figure 1d).

## Results and Discussion

### Synthesis and Reactivity of the Cross-Linking Reagent

Olefins readily undergo cycloaddition with activated thianthrene-S-oxides to form [4 + 2] dicationic cycloadducts that can then be deprotonated to yield alkenyl thianthrenium salts.^20^ Our aim was to synthesize an episulfonium precursor from an olefin equipped with a terminal alkyne tag and to ensure that the resulting reagent retained cell permeability comparable to **VTT** despite chemical modification. A short linker that contains an ether group was designed to compensate for introduction of the lipophilic alkyl chain and provide minimal influence on the reagent’s polarity and water solubility. Thianthrenation of 3-allyloxy-1-propyne led to formation of a water-soluble, bench-stable [4 + 2] cycloaddition product (**TTD**, **1**) from commercially available reagents in a single step in 82% yield on a gram scale (Figure 2a). Unlike the symmetrical dication used in the synthesis of **VTT**,^21^ the unsymmetrical **TTD** forms a mixture of Cys-reactive alkenyl thianthrenium salts upon rapid (*t*_1/2_ = 3 s) deprotonation in sodium phosphate buffer (see Supporting Information, Figure S6). Calculations revealed that the formed isomers exhibit size and polarity similar to those of **VTT**. For example, the calculated lipophilicity index (logP) values (4.28–4.42) are slightly smaller than that of **VTT** (4.64), whereas the calculated topological polar surface area (TPSA) of each compound is 59.8 Å^2^, only 18% higher than the calculated TPSA of **VTT** (50.6 Å^2^). The similar logP and TPSA suggest that the formed alkenyl thianthrenium salts, like **VTT**, should be able to penetrate cell membranes to react with proteins *in cellulo*.^22^

**Figure 2:**
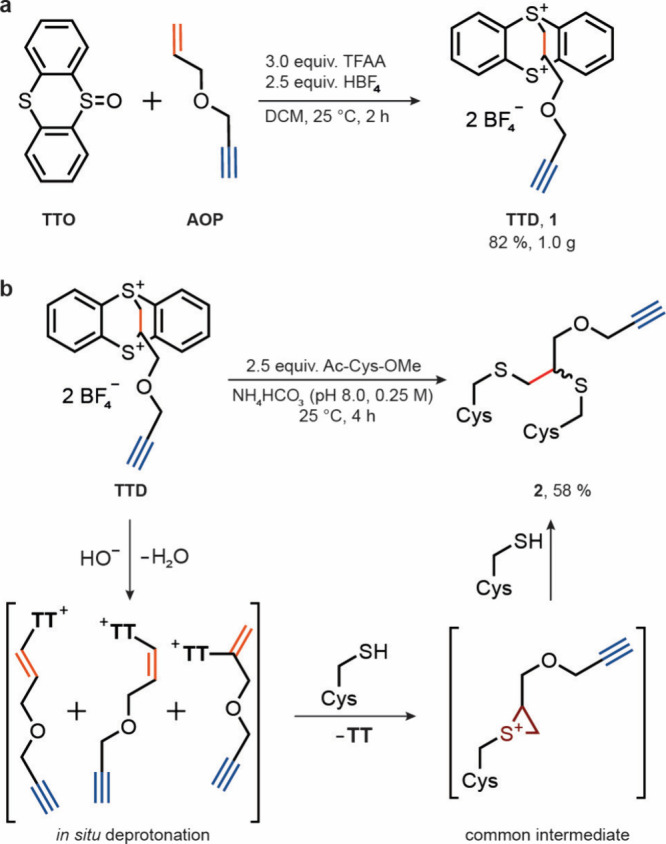
Synthesis and reactivity of thianthrenium (**TT**) dication **TTD**. (a) Single-step synthesis of alkyne-containing thianthrenium reagent **TTD**. TTO, thianthrene-*S*-oxide; AOP, 3-allyloxy-1-propyne; TFAA, trifluoroacetic anhydride; DCM, dichloromethane. (b) Proposed mechanism for the reaction of **TTD** with *N*-acetyl-l-cysteine-methylester (Ac-Cys-OMe) in ammonium bicarbonate buffer.

A single constitutional isomer is obtained when **TTD** reacts with two equivalents of cysteine in basic ammonium bicarbonate buffer (Figure 2b). Due to the fast deprotonation of **TTD**, the reactive alkenylthianthrenium salts are formed *in situ* and prior to the reaction of **TTD** with Cys. Subsequent Michael addition and intramolecular thianthrene displacement leads to formation of a common episulfonium intermediate. To exclude competing nucleophilic substitution chemistry, we conducted the reaction in a deuterated buffer and observed incorporation of a single deuterium atom into the formed product (see Supporting Information, Figures S7–S11). This observation is consistent with the formation of a sulfonium ylide intermediate subsequent to the Michael addition and excludes formation of the product via nucleophilic substitution reaction at **TTD** that would not lead to deuterium incorporation. The overall transformation proceeds in a single operation because the formation of the reactive episulfonium species leads to rapid reaction with nucleophiles in close proximity. To test whether **TTD** can be used directly for protein cross-linking without the need to isolate single alkenylthianthrenium isomers, we treated the Ubiquitin K63C mutant with **TTD** in phosphate buffer in the absence of additional exogeneous nucleophiles and observed species with a mass consistent with that of cross-linked products formed by intramolecular episulfonium opening as the main product, along with the product resulting from episulfonium opening with water (see Supporting Information, Figures S18–S22). To further establish the applicability of dicationic thianthrenium reagents for direct use in bioconjugation, we analyzed product mixtures obtained when the Ubiquitin K63C mutant was treated with either **VTT** or its dicationic precursor (see Supporting Information, Figures S23–S35). The identical product distribution in both cases indicates that *in situ* deprotonation of the [4 + 2] dicationic thianthrenium cycloadducts is facile and therefore does not have to be performed as a part of the reagent synthesis. Moreover, no reaction was observed between **TTD** and native Ubiquitin that contains no reduced Cys residues (see Supporting Information, Figures S36–S38). The results indicate that **TTD** can serve as an alternative to **VTT** for Cys-selective protein cross-linking that requires downstream enrichment of the cross-linked products and confirm that deprotonation of bench-stable **TTD** in a separate step is not necessary to achieve protein cross-linking.

### TTD-Mediated *in Cellulo* Alkyne Incorporation

To determine whether **TTD** can label cysteine residues *in cellulo*, we treated confluent HEK cells with **TTD** and washed the cells with phosphate-buffered saline (PBS) before lysis. All reaction products that result from nucleophilic ring-opening of the episulfonium intermediate possess an alkyne tag and should serve as substrates for subsequent cycloaddition reactions. The cell lysates were subjected to a copper-catalyzed azide–alkyne cycloaddition (CuAAC) reaction with a fluorophore to visualize proteins labeled *in cellulo*. Sodium dodecyl sulfate–polyacrylamide gel electrophoresis (SDS-PAGE) revealed proteome-wide fluorescence, indicating efficient cell penetration and alkyne diversification, both crucial for follow-up enrichment studies with the obtained mixtures (Figure 3a). To confirm that protein labeling occurred with high Cys-selectivity within a whole proteome, we set up a CuAAC reaction with the lysate and biotin-azide, followed by reduction, alkylation, and trypsin digestion. We then performed an affinity enrichment of modified digested peptides with NeutrAvidin beads that enable isolation of biotin-modified peptides from the resin without addition of detergents.^23^ LC–MS/MS analysis of the enriched peptides revealed 86% site-selectivity for cysteine in the intracellular environment (Figure 3b). To assess the enrichment specificity, we compared the number of peptides that carried the biotin modification to the number of unmodified peptides. Only 7.4 ± 0.1% of the detected peptides were not modified with biotin, which indicated a high enrichment specificity.

**Figure 3:**
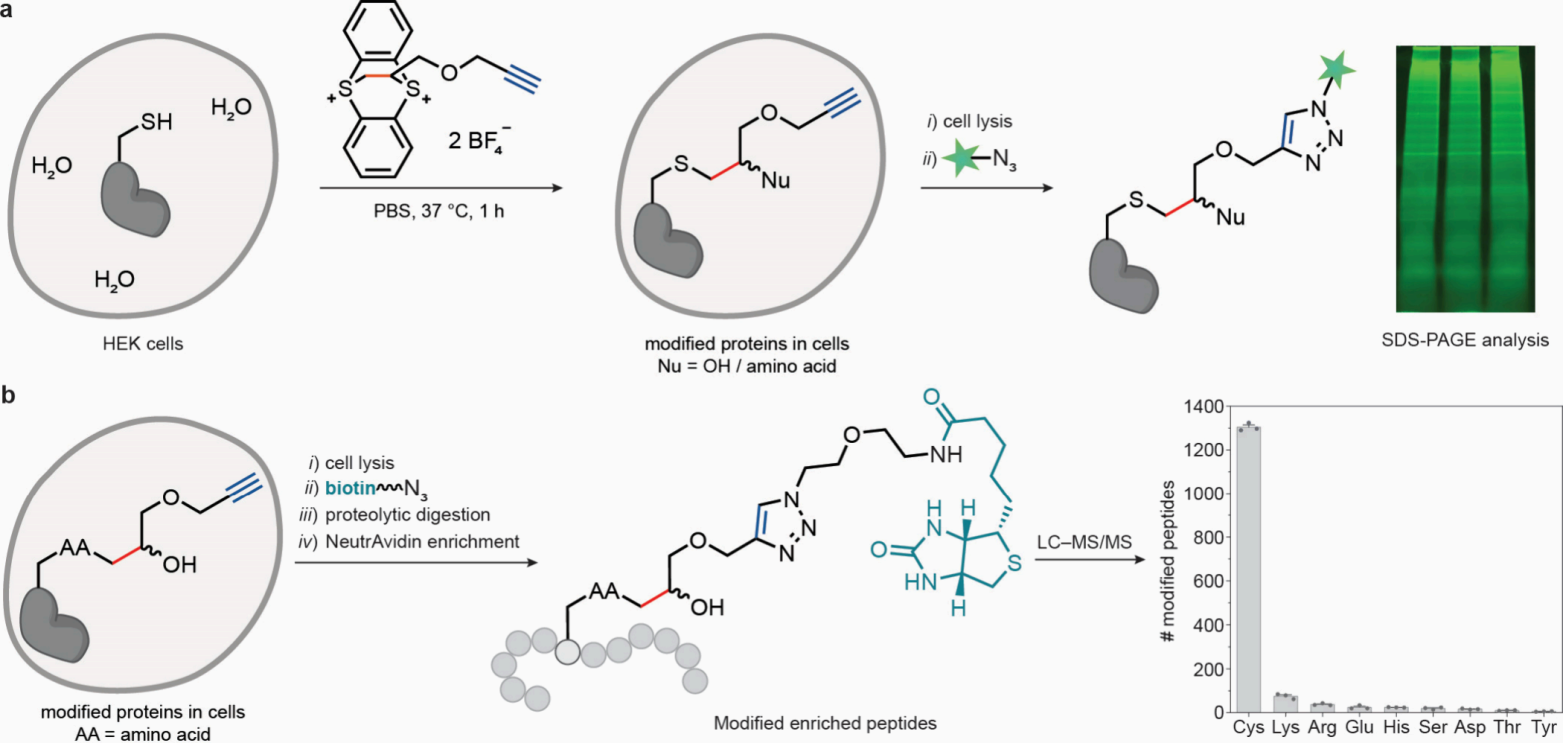
*In cellulo* incorporation and follow-up modifications of the alkyne tag. (a) Proteome-wide alkyne incorporation and fluorescence SDS-PAGE analysis of the isolated lysates from three biological replicates after the CuAAC reaction with 6-FAM (6-carboxyfluorescein) azide. PBS, phosphate-buffered saline; Nu, nucleophile; AA, amino acid. (b) Biotin-based enrichment of modified peptides and selectivity profile of modified peptides detected by LC–MS/MS. The values are means ± SE of *n* = 3 independent experiments. Only one of the two possible constitutional isomers for each product is depicted in each case.

### Cross-Linking Data Analysis

After establishment of the cysteine selectivity and enrichment specificity, we performed a cross-link search for peptides in which Cys is covalently bound to a nucleophilic amino acid (Glu, Asp, Ser, Thr, Cys, His, Tyr, Lys, Arg), C-, and N- protein termini, respectively. To ensure the reliable identification of protein–protein interactions from cross-linking experiments we applied a rigorous false discovery rate (FDR) control.^24^ The search results were filtered to retain only those with a FDR below 1%, yielding 1534 intraprotein and 100 interprotein cross-links among three biological replicates. Based on the 1% threshold that was set for the FDR estimation, it is expected that only one of 100 identified interprotein cross-links is a false positive.^25,26^ Gene-ontology (GO) annotation of cross-linked proteins revealed that the cross-link formation was achieved for proteins in all major cell compartments, including challenging targets for chemoproteomic labeling, such as nucleus and mitochondria^27,28^ (Figure 4a). Cross-links to all nucleophilic amino acids and the C-terminus were detected among the identified intermolecular cross-links (Figure 4b), which were mapped to 39 proteins and 22 PPIs. To examine the chemoselectivity of the episulfonium intermediate toward different nucleophilic amino acid residues, we compared amino acid frequency distributions in the identified cross-links with those in the proteome. The analysis revealed no increased selectivity for nonbasic amino acid residues, in line with high reactivity of the **TTD**-derived episulfonium intermediate (see Supporting Information, Figure S40) and the data obtained for **VTT**-mediated cross-linking.^19^ For example, the sterically hindered secondary alcohol of Thr, the aromatic hydroxy group of Tyr, and the carboxylate of Glu or Asp can all be captured by the substituted episulfonium intermediate and thereby increase the probability of short-distance PPI detection. Basic amino acids Arg and Lys are less frequently identified within the detected cross-links, consistent with their lowered nucleophilicity at the physiological pH in cells. The high reactivity of the episulfonium intermediate often ensures formation of multiple cross-links on the interface of interacting proteins and therefore further enhances identification confidence in the obtained data sets and minimizes false-discovery rates. In our data set, only two PPIs were supported by fewer than three interprotein cross-links (Figure 4c). To ensure a high level of confidence, these PPIs were excluded from the subsequent analysis.^29^

**Figure 4:**
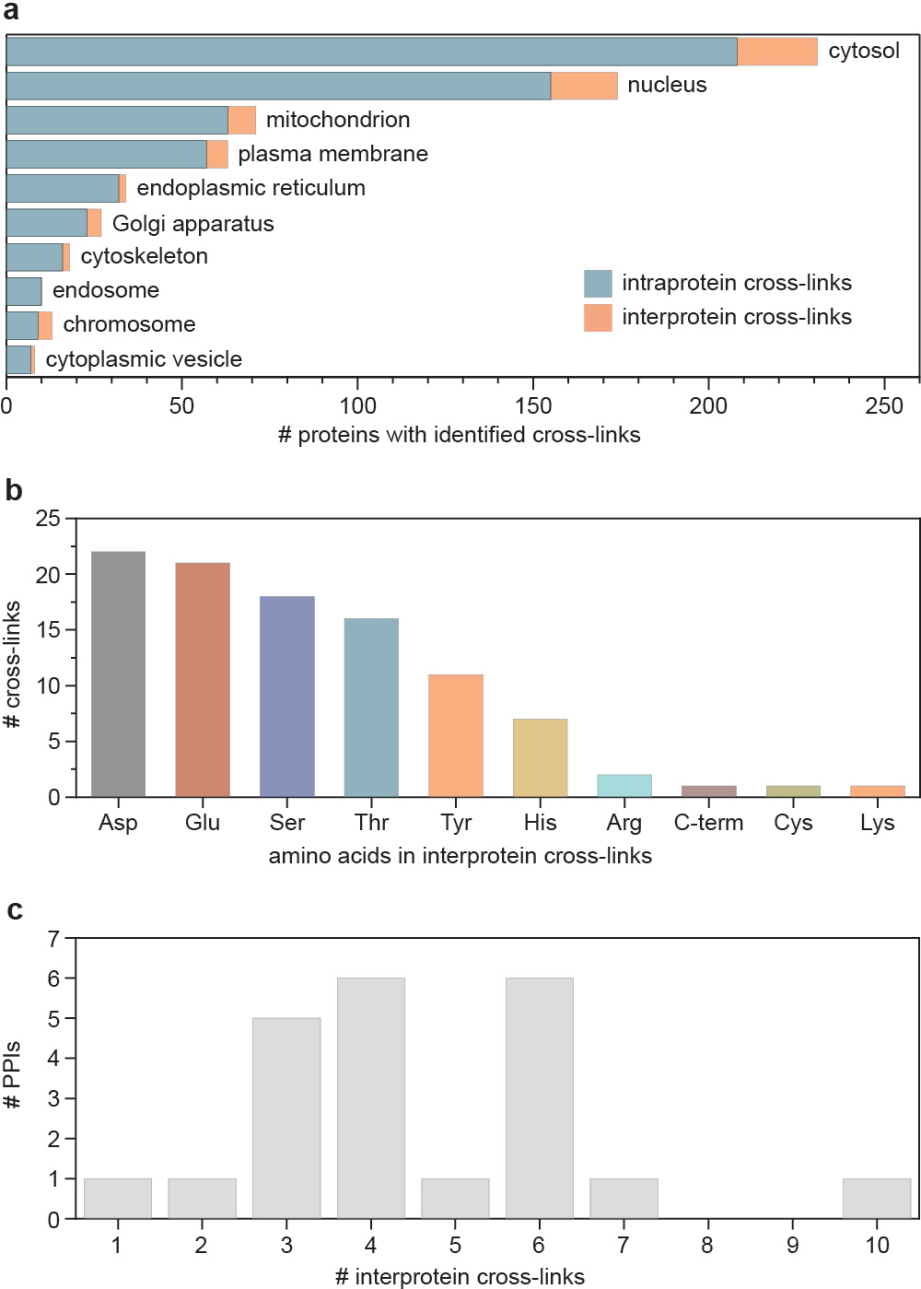
Overview of the cross-linking data. (a) Gene ontology-annotated intracellular localization of proteins for which at least two cross-links were detected in the data set. (b) Distribution of amino acids in the detected heteromeric cross-links. (c) Number of heteromeric cross-links per identified PPI.

### Validation of Generated Distance Restraints

Among the 20 PPIs with at least three identified intermolecular cross-links, 15 interactions are annotated in the STRING database.^30^ We used STRING database scores to assess the validity of the identified PPIs.^31^ All detected PPIs have STRING scores above the medium-confidence threshold of 0.5, with only two interactions below the high-confidence cutoff of 0.9. This result indicates that the fast episulfonium-mediated cross-link formation and strict false-discovery rate control of the obtained cross-linking results provide PPI data of high confidence. We benchmarked the validity of the cross-link restraints by determining the distances between **TTD**-cross-linked residues in the reported structure of interacting proteins CCT5 and CCT7 in the TCP-1 ring complex, an essential protein-folding chaperonin.^32^ The reported structure of this PPI accommodates all residues cross-linked with **TTD** within its interaction interface, and was selected to validate the calculated distance restraints with identified cross-links on an experimentally verified PPI structure. The C_α_ atoms of all cross-linked residues are only up to 17 Å away from the C_α_ of the Cys backbone (Figure 5a), consistent with the distance range of 4–23 Å for ethylene-bridged amino acids,^19^ similarly precise as for the 10–25 Å distances obtained from photo-cross-linking approaches.^33^ These short interprotein cross-links support the high reactivity of generated episulfonium intermediates that undergo immediate trapping with neighboring residues and indicate no negative influence of the incorporated alkyne tag on the obtained distance restraints. The obtained data suggested that the short distance restraints of up to 23 Å can be used for modeling PPI structures based on the **TTD**-derived cross-linking data, and that our method compares favorably to bifunctional lysine-selective reagents that provide distance restraints at least 10 Å longer than that of **TTD**.^34,35^ We then conducted protein docking studies with CCT5 and CCT7 based on the **TTD** distance restraints to verify that docking studies with the obtained cross-links enable reliable prediction of the PPI interface. The predicted PPI structure has high alignment with the reported PDB structure of the proteins in the TCP-1 ring complex, as exemplified by the DockQ^36^ score of 0.95 (Figure 5b), and further verifies the potential of the method in high-confidence PPI modeling.

**Figure 5:**
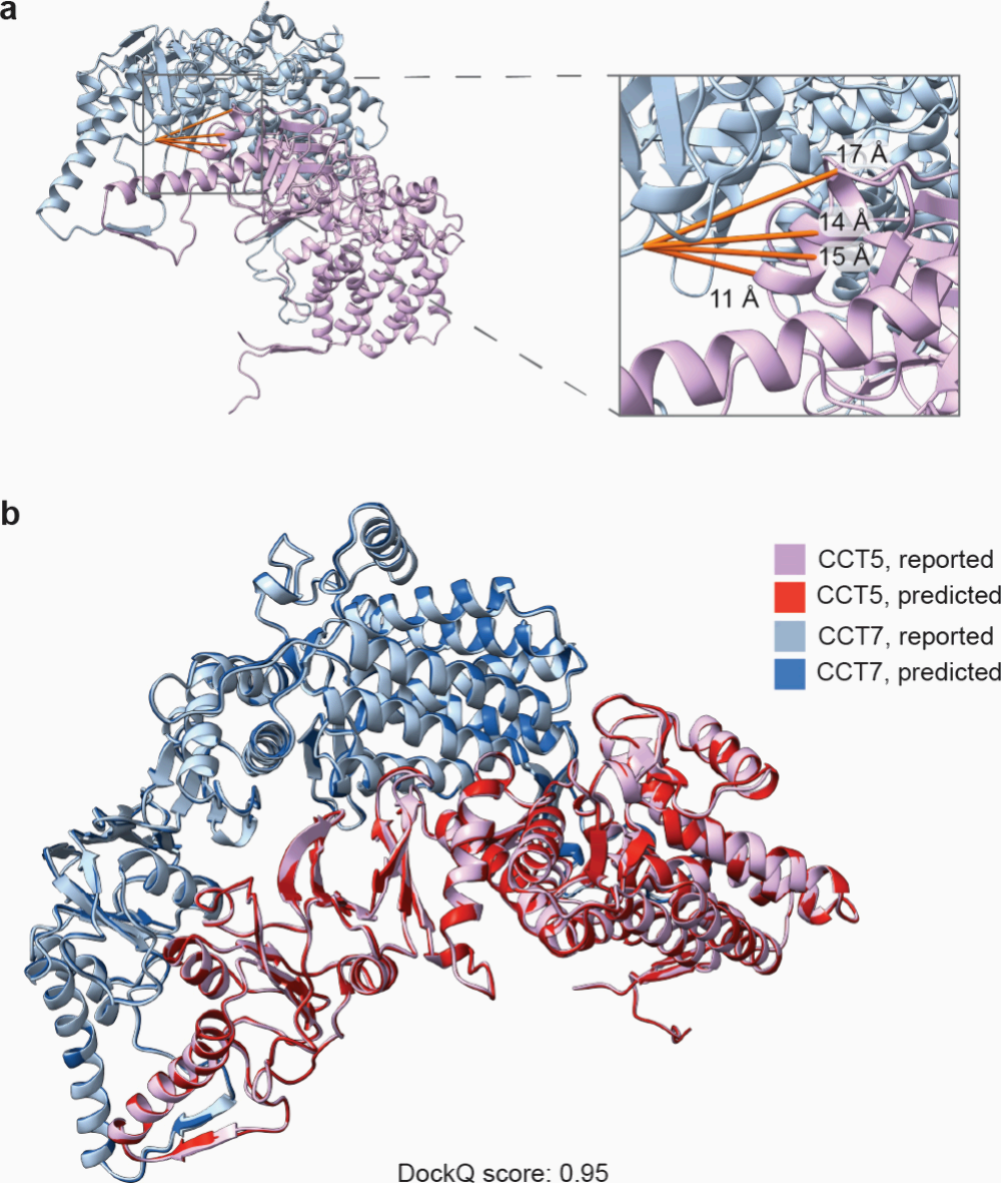
Cross-link mapping and PPI structure alignment. (a) Reported structure of interacting proteins in the chaperonin TCP-1 ring complex with the mapped detected cross-links. (b) Evaluation of cross-linking-mediated approach for structure prediction of interacting proteins CCT5 and CCT7. Docking was performed with HADDOCK 2.4,^37^ and the structure was aligned with the reported PDB structure (7NVL) and visualized using Chimera X 1.10.^38,39^

### PPI Structure Prediction for Reported PPIs

Based on the short distance restraints produced by **TTD**, we hypothesized that the obtained cross-linking data can be used to model the structures of PPIs that were previously identified but lack reliable structural data. We first examined the PPI between VDAC2 and VDAC3, pore-forming β-barrel channels embedded in the outer mitochondrial membrane, where they control ATP, ADP, and ion exchange, and act as regulators of mitochondria-driven cell-death pathways.^40–42^ Studies with the antitumor drug Erastin showed that both VDAC2 and VDAC3 are required for ferroptosis, an iron-dependent oxidative cell death.^43–45^ In mammalian cells, the interaction has been identified in large-scale affinity-purification experiments, indicating that the two proteins reside in the same protein complex on the organelle surface.^46,47^ Despite the available functional evidence, no experimental structure or mutational mapping of the interaction interface exists. Furthermore, prediction tools like AlphaFold-Multimer that solely rely on statistical patterns from structural data provide only low-confidence models.^48,49^ We modeled the VDAC complex using **TTD**-derived cross-linking restraints and obtained a structure consistent with the expected natural parallel orientation of the β-barrels within the mitochondrial membrane^50–52^ (Figure 6a). When docking was performed based on distance restraints obtained from previously reported data^27^ with the lysine-selective cross-linker **DSSO** (disuccinimidyl sulfoxide),^53,54^ the top-scoring model suggested an orthogonal orientation of the two ion channels, which is not in agreement with the biological function of ion channel proteins as well as the reported structures of other VDAC complexes.^50–52^ The discrepancy is likely caused by the long C_α_–C_α_ restraints of up to 35 Å produced by **DSSO**,^53,54^ which are too flexible to ensure the naturally parallel orientation of the two proteins. The result demonstrates that our strategy provides shorter and more reliable distance restraints than Lys-selective cross-linkers.

**Figure 6:**
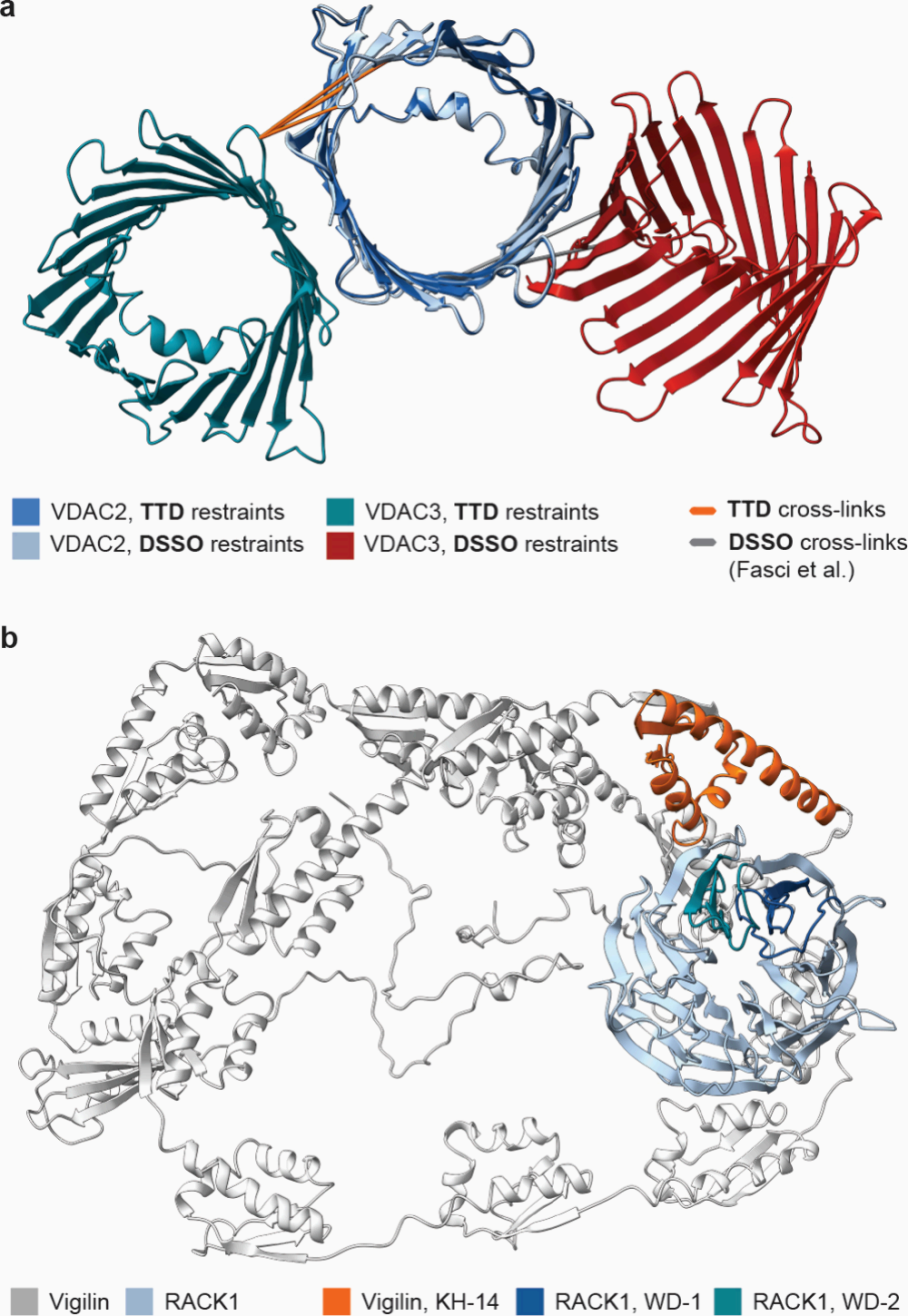
Predicted PPI structures of the reported interactions. (a) Predicted structure of interaction between VDAC2 and VDAC3 based on cross-linking restraints obtained in this study or reported by Fasci et al.^27^ and mapped identified cross-links. (b) Predicted structure of interaction between RACK1 and Vigilin based on the obtained cross-linking restraints. Docking was performed with HADDOCK 2.4,^37^ and the structures were visualized using Chimera X 1.10.^38,39^

In addition to the identified cross-links between VDAC proteins, our data included seven cross-links between RACK1, a conserved scaffolding protein, and Vigilin, an RNA-binding regulator of mRNA translation.^55,56^ A recent RACK1 interactome study showed that the association with Vigilin is required for efficient dengue-virus replication in mammalian cells, making the interaction a potential target to address a disease with no approved antiviral therapeutics.^57^ Consistent with the previously reported data on the binding between the RACK1 and Vigilin homologues in yeast, coimmunoprecipitation experiments in HEK293 cells indicated that the KH-14 domain in Vigilin is necessary for the complex formation with RACK1, but no structure of the interacting proteins was reported.^57,58^ We performed a docking study based on the **TTD**-identified cross-linking restraints and obtained a PPI structure that is consistent with an interaction between Vigilin’s C-terminal KH-14 domain and WD-1 and WD-2 domains of RACK1 (Figure 6b). Our study provides the first chemical cross-linking data on the association between Vigilin and RACK1 in mammalian cells and enables prediction of the PPI interface based on the experimentally obtained distance restraints.

### Identification of Previously Unreported PPIs

Having established that our method enables characterization of the interaction surfaces for PPIs without available structural data, we then reviewed the PPIs detected by our approach and identified those protein–protein interactions that have not been previously reported. Apart from the RACK1 interaction with Vigilin, we detected a previously undocumented interaction of RACK1 with CDV3, a protein that is associated with tumorigenesis in colon, stomach, and liver.^59^ Our data set contains other PPIs that were not identified before, and involve the kinase RACK 1,^55^ the dehydrogenases MDH2 ^60^ and PHGDH,^61^ the hydrolase EPHX1,^62^ and the deglycase PARK7 ^63^ that all have been linked to cancer-induced dysregulation. The GO-annotation of the cellular localization indicates that all proteins in the identified PPIs are localized in the same or adjacent cellular compartments with reported cross-compartment interactions^64–66^ (Figure 7a). While further experiments are needed to determine whether the detected PPIs are transient interactions or stable complexes, one of the identified PPIs, between the centrosomal protein of 112 kDa (CEP112) and the D-3-phosphoglycerate dehydrogenase (PHGDH), is supported by cross-links that were detected in two biological replicates, which indicate the formation of a stable interaction complex. PHGDH is a cytosolic enzyme that catalyzes the rate-determining step of the l-serine biosynthesis pathway and imparts cytosolic protein synthesis.^61^ Because CEP112 has been implicated in local control of translation at the centrosome,^67^ and metabolic enzymes have been reported to associate with centrosomal proteins,^68^ we speculate that, if PHGDH and CEP112 interact, PHGDH-dependent serine metabolism might influence centrosome-associated translation^69^ and thereby affect cell differentiation via interaction with CEP112. We performed docking studies to predict the putative structure of the interaction surface using **TTD**-derived distance restraints and obtained a model that suggests binding of PHGDH to the N-terminal region of CEP112 (Figure 7b). Analysis of interchain atom–atom contacts^70,71^ revealed that the predicted interaction interface is dominated by electrostatic interactions (see Supporting Information, page S36). The calculated dissociation constant of 3 × 10^–7^ M at 25 °C is compatible with formation of a medium-affinity interaction complex^72^ and thereby the identification of the PPI in two biological replicates. The results indicate that Cys-selective interprotein cross-linking with **TTD** and the efficient enrichment of short-distance cross-linked products can be used to identify previously undocumented PPIs in cells and model the interaction surface using a set of reliable distance restraints. We expect our results to provide a starting point for detailed studies of the identified PPIs, given the localization compatibility of the proteins in the unreported PPIs.

**Figure 7:**
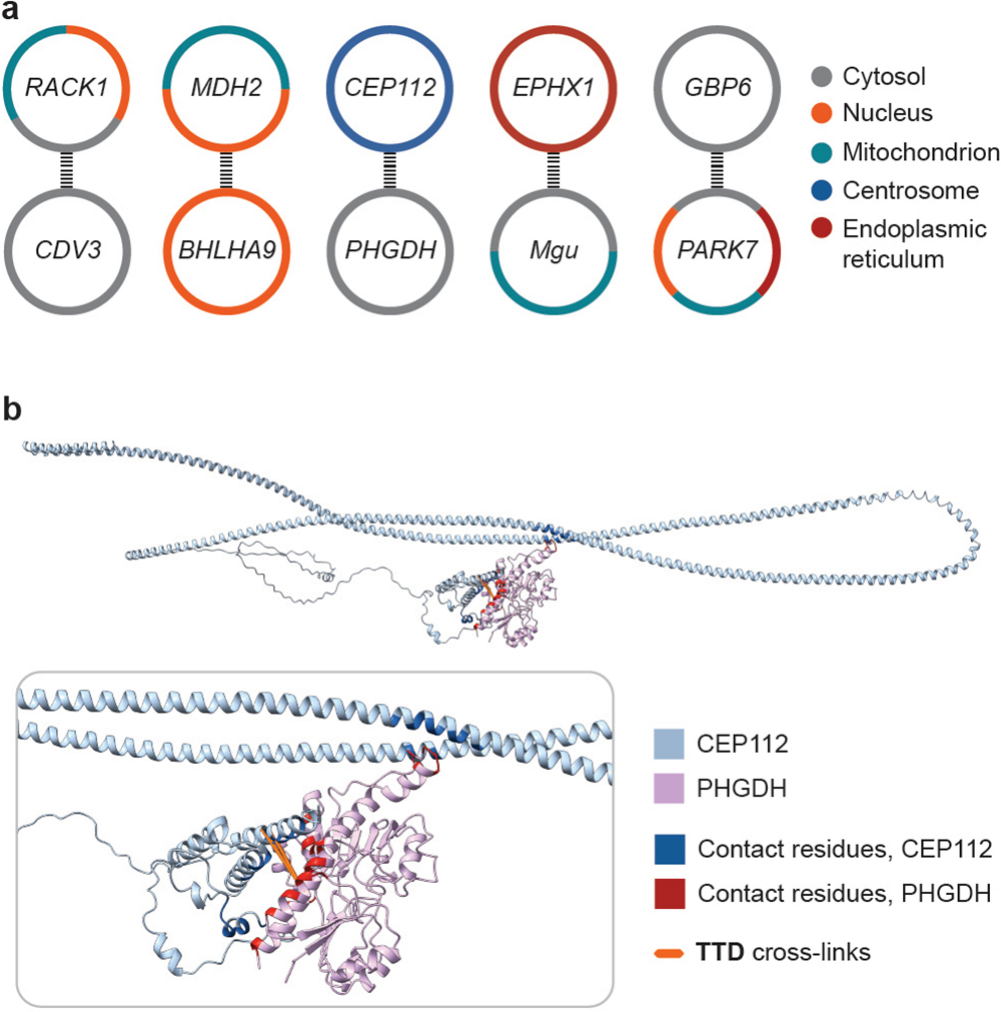
Cellular localization for proteins in the previously unreported PPIs and putative structure of interaction between PHGDH and CEP112. (a) Annotation of cellular localization for proteins in previously undocumented PPIs that were identified in this work. Dashed lines indicate detected PPIs between the proteins. RACK1, receptor of activated protein C kinase 1; CDV3, protein CDV3 (carnitine deficiency-associated gene expressed in ventricle 3) homologue; MDH2, mitochondrial malate dehydrogenase; BHLHA9, class A basic helix–loop–helix protein 9; CEP112, centrosomal protein of 112 kDa; PHGDH, d-3-phosphoglycerate dehydrogenase; EPHX1, epoxide hydrolase 1; Mgu, uroporphyrinogen-III synthase; GBP6, guanylate-binding protein 6; PARK7, Parkinson disease protein 7. (b) Predicted structure of interaction between PHGDH and CEP112, zoom on the identified contact residues and mapped **TTD**-derived cross-links. Docking was performed with HADDOCK 2.4;^37^ contact residues were identified using PRODIGY,^70,71^ and the structure was visualized using Chimera X 1.10.^38,39^

## Conclusions

We have developed a reagent that enables single-step cross-linking, identification, and characterization of PPIs in a cellular environment via umpolung of native Cys residues into reactive episulfonium intermediates. The synthetically readily available thianthrenium reagent enables proteome-wide Cys-selective labeling and incorporates an alkyne tag for enrichment of the modified products without the need for linker elongation. We have established that Cys-reactive alkenylthianthrenium can be rapidly generated *in situ*, and that deprotonation of thianthrenium cycloaddition products as a separate synthetic step is redundant. Although low-abundant Cys residues are targeted with our approach, the formation of reactive episulfonium intermediates ensures efficient capturing of short distance PPIs via rapid formation of multiple cross-link pairs to all nucleophilic amino acids. The short distance restraints enabled by high episulfonium reactivity allow reliable modeling of PPI interfaces and provide a less error-prone alternative to the widely used bifunctional reagents with long linker lengths. Because naturally low abundant Cys can be readily incorporated into proteins, we anticipate that our method will provide an efficient one-step protocol for *in cellulo* interactome studies based on the cross-linking between native residues.

## Supplementary Material



## Notes

Experimental procedures, analytical methods, and spectroscopic data (PDF)
