# Supplementary material for: Thianthrenium Chemistry for Identification of Protein–Protein Interactions in Cells
Source: J Am Chem Soc. 2025 Dec 1;147(49):45576–84. doi: 10.1021/jacs.5c16665 (PMC12703657; doi:10.1021/jacs.5c16665)
Supplement: Supplementary file 1 [file ja5c16665_si_001.pdf]

SUPPORTING INFORMATION

Thianthrenium chemistry for identification of protein−protein interactions in cells

Kostiantyn Bohdan[1,2,‡], Philipp Hartmann[1,2,‡], Sven Müller[1,2], Dario Marchionni[1], Christian Preisinger[3], Julia Beatrice Jacobs[1], Lara Vogelsang[4], Marie Sophie Sterling[1], Karl-Josef Dietz[4], and Tobias Ritter[*,1]

[1]Max-Planck-Institut für Kohlenforschung, Kaiser-Wilhelm-Platz 1, 45470 Mülheim an der Ruhr, Germany.

[2]Institute of Organic Chemistry, RWTH Aachen University, Landoltweg 1, 52074 Aachen, Germany.

[3]Proteomics Facility, Interdisciplinary Centre for Clinical Research (IZKF), RWTH Aachen University, 52074 Aachen, Germany.

[4]Biochemistry and Physiology of Plants, Faculty of Biology, Bielefeld University, Universitätsstraße 25, 33615 Bielefeld, Germany.

‡These authors contributed equally.

*E-mail: ritter@kofo.mpg.de

TABLE OF CONTENTS

TABLE OF CONTENTS 1

ABBREVIATIONS 3

MATERIALS AND METHODS 4

Starting Materials 4
NMR Spectroscopy 4
Mass Spectrometry 4
LC–MS 4
LC–MS/MS 5
SDS-PAGE 5
Miscellaneous 6

EXPERIMENTAL DATA 7

Synthesis and characterization of small molecules 7

Synthesis of **1** 7
Synthesis of **2** 7
Stability of **1** under ambient atmosphere 9
Deprotonation of **1** in phosphate buffer 9
Deprotonation half-life of **TTD** in NaP_i 10
Calculated TPSA and logP 11

Deuterium incorporation studies 12

Product characterization using NMR 12
Product characterization using MS 14

Reactivity with proteins 15

Characterization of protein starting materials 15
Reactions with Ubiquitin K63C 17
Control experiment with Ubiquitin 25

Experiments with HEK cells 27

Sample preparation for LC–MS/MS measurements 28

CuAAC reaction with biotin-azide 28
Protocol for biotin enrichment 28

SDS-PAGE analysis 30

CuAAC reaction with 6-FAM-azide 30

DATA ANALYSIS 31
Labeling efficiency and enrichment specificity 31
Cross-link data analysis 32
Cross-link search 32
Identified PPIs 33
GO annotations 34
Docking studies 35
General 35
CCT5 and CCT7 35
VDAC2 and VDAC3 35
RACK1 and HDLBP 35
CEP112 and PHGDH 36
Data availability 37
NMR DATA 38
NMR Characterization of **1** 38
NMR Characterization of **2** 41
Alkenyl TT salts **1e**, **1z**, **1t** 50
Deprotonation product **1e** 50
Deprotonation product **1z** 51
Deprotonation product **1t** 52
REFERENCES 60

ABBREVIATIONS

Abbreviation	Full Name
BCA	Bicinchoninic Acid
CuAAC	Copper-catalyzed azide-alkyne cycloaddition
DMEM	Dulbecco's Modified Eagle Medium
DCM	Dichlormethane
DMSO	Dimethyl sulfoxide
DPBS	Dulbecco's Phosphate-Buffered Saline
DTT	Dithiothreitol
EDTA	Ethylenediaminetetraacetic acid
6-FAM	6-Carboxyfluorescein
FCS	Fetal Calf Serum
FDR	False discovery rate
FWHM	Full Width at Half Maximum
HCD	Higher Energy C Trap Dissociation
MQ	Milli-Q
NP-40	Nonidet P-40
PBS	Phosphate-Buffered Saline
PEG	Polyethylene Glycol
SDS	Sodium Dodecyl Sulfate
SP3	Single-pot, solid-phase-enhanced sample preparation
TCEP	Tris(2-carboxyethyl)phosphine
THPTA	Tris(3-hydroxypropyltriazolylmethyl)amine
TPSA	Topological polar surface area
VTT	Vinylthianthrenium tetrafluoroborate

MATERIALS AND METHODS

Starting Materials

Reagents

3-(Allyloxy)-1-propyne was purchased from Sigma Aldrich (#763659). Biotin-PEG1-azide was purchased from BroadPharm (#BP-40775). 6-FAM azide was purchased from Carl Roth (#7806.2). THPTA was purchased from TCI Chemicals (#T3171). TCEP•HCl was purchased from Carl Roth (#HN95.1). DTT was purchased from Chempur (#GC3928). **VTT** and the dicationic **VTT** precursor were synthesized as previously described[1].

Solvents

Water used to prepare buffers and as a solvent was of ultra-high quality (UHQ) grade (18.2 MΩ·cm^{-1}). Acetonitrile (>99%) and dimethyl sulfoxide (>99%) were purchased from Fisher Scientific. Anhydrous DCM was obtained from Phoenix Solvent Drying Systems.

NMR Spectroscopy

Chemical shifts are reported in ppm (δ) relative to tetramethylsilane (TMS) with the solvent (residual) peak as the internal standard. For ^{1}H NMR: HDO, δ 4.79; CHD_2CN, δ 1.94; DMSO-d_6, δ 2.50. For ^{13}C NMR: CD_3CN, δ 1.32; DMSO-d_6, δ 39.52. ^{19}F and ^{15}N NMR spectra were referenced relative to ^{1}H using a unified chemical shift scale. Data are reported as follows: s = singlet, d = doublet, t = triplet, q = quartet, quint = quintet, m = multiplet, br = broad; coupling constants in Hz.

NMR spectra were recorded on following instruments:

1. Bruker Avance III 500 spectrometer equipped with a BBFO probe head, operating at 500 MHz and 471 MHz, for ^{1}H and ^{19}F acquisitions, respectively

2. AVANCE III 600 spectrometer equipped with a triple-channel “TCI” cryogenic probehead (Bruker GmbH, Rheinstetten) operating at 600 MHz, 61 MHz, and 151 MHz, for ^{1}H, ^{15}N, and ^{13}C acquisitions, respectively. All experiments used standard Bruker pulse sequence with standard parameter sets found in libraries of Topspin 3.6.

Mass Spectrometry

High resolution Mass Spectrometry (HRMS) experiments for small molecules and peptides were performed on a Thermo Scientific™ Q Exactive Plus or a Thermo Scientific™ Q Exactive GC Orbitrap device.

LC–MS

Measurements were performed on Shimadzu LCMS-9030 with SCL-40, 2x LC-40D XS, SIL-40C XS, CTO-40C, SPD-M30A, equipped with an YMC mm Accura Triart Bio C4 column, 100 mm × 2.1 mm, 3 µm, flow rate 0.2 mL / min, 323 K.

Eluent A: 0.1% formic acid in UHQ-H_2O; Eluent B: acetonitrile + 0.1% formic acid.

The samples were separated using a linear gradient method. Initially, the gradient began with an isocratic condition of 10% B for 2.5 minutes, transitioning to 80% B over a period of 12.5 minutes. This was followed by a 3-minute isocratic run at 80% B. Subsequently, a linear gradient was applied to return to 10% B over 18 seconds.

Online desalting of the samples was performed using an Agilent AdvanceBio Desalting-RP column (dimensions: 12.5 x 2.1 mm) with a mobile phase of 10% B from 0.32 to 2.5 minutes, at a flow rate of 0.4 mL/min. Sample injection occurred at 0.1 minutes. Absorption spectra were recorded at a wavelength of 214 nm using a diode array detector (DAD).

LabSolutions 5.128 and LabSolutions Insight Explore 4.2 software was used for analysis and deconvolution.

LC–MS/MS

Acquisition of mass spectra was performed on an Exploris 480 mass spectrometer (Thermo Scientific) coupled to an RSLCnano UHPLC system (Thermo Scientific). The samples were dissolved in 3% (v/v) formic acid / 1% (v/v) acetonitrile, loaded onto a trapping column (Acclaim PepMap100, C18, 5 µm, 100 Å, 300 µm i.d. × 5 mm, Thermo Scientific) over 10 min, and then eluted onto the analytical column. Separation was achieved on an Aurora Ultimate column (C18, 25 cm, 75 µm internal diameter, IonOpticks) maintained at 45 °C. The peptide separation was performed with a binary mobile phase gradient using 0.1% (v/v) aqueous formic acid as mobile phase A and 80% (v/v) acetonitrile / 0.1% (v/v) formic acid as mobile phase B with a flow rate of 300 nL min^{-1}: 0–2 min, 2% B; 2–105 min, 2→30% B; 105–130 min, 30→40% B; 130–137 min, 40→99% B; 137–142 min, 99% B; 142–145 min, 95→2% B; and 145–160 min, 2% B. Electrospray ionization was achieved with a 2 kV spray voltage and a capillary temperature of 275 °C.

MS data were acquired in data-dependent mode based on the previously reported settings[2]. The MS^1 spectra were recorded over m/z 375–1500 at a resolution of 60,000 with an automated gain control (AGC) of 300% and a minimum intensity threshold of 5 × 10. Precursors with charge states from +3 to +8 were selected for fragmentation with dynamic exclusion set to automatic. Fragment spectra of the top 10 precursors were acquired at a resolution of 30,000, using a 1 Da isolation window, normalized stepped HCD collision energies of 21%, 27%, and 33%, normalized AGC of 100%, and a maximum injection time of 64 ms.

SDS-PAGE

SDS-PAGE sample preparation and analysis was carried out as instructed in the manual of Bio-Rad[3]. Fluorescence measurements were carried out as instructed in the manual of Bio-Rad utilizing the “Fluorescein” analysis of the Bio-Rad Laboratories ChemiDocMP system. Coomassie Blue staining of the gels was performed as instructed by the manufacturer. Fixation was performed for 15 min and Coomassie Blue staining was performed for 20 h. All proteins were analyzed with Tris/Glycine/SDS gel electrophoresis.

Image Lab Version 6.1.0 build 7 software from Bio-Rad Laboratories Inc. was used for analysis and processing of SDS-PAGE data.

Reagents used in the SDS-PAGE workflow:

Bio-Rad Laboratories Any kD MP TGX Stain-Free 10W 30 µL

Bio-Rad Laboratories 10x Tris/Glycine/SDS buffer

Bio-Rad Laboratories 4x Laemmli sample buffer

Bio-Rad Laboratories QC Colloidal Coomassie stain

Bio-Rad Laboratories Precision Plus Protein™ Dual Xtra Prestained Protein Standards

Devices used in the SDS-PAGE workflow:

Bio-Rad Laboratories ChemiDocMP

Bio-Rad Laboratories Mini-PROTEAN Tetra Cell

Bio-Rad Laboratories PowerPac Basic

Miscellaneous

Lyophilization of purified products was performed using a BÜCHI Lyovapor™ L-200. Reactions were conducted in an Eppendorf ThermoMixer® C equipped with an Eppendorf SmartBlock™ 1.5 mL. Sample preparation for LC–MS/MS analysis was performed in Protein LoBind® tubes from Eppendorf. Protein rebuffering was performed with AMICON® filters units from Sigma Aldrich. Lysate and protein concentrations were determined via BCA assay (ThermoFisher, #23225) using a Thermo Scientific™ NanoDrop™ One^{C}. All UV-vis measurements were recorded on a Shimadzu UV-vis Spectrophotometer UV-2600 with temperature controller Shimadzu S-1700 at (25 ± 0.1) °C.

Unless stated otherwise, Milli-Q water (MQ-H_2O) was used in all experiments.

EXPERIMENTAL DATA

Synthesis and characterization of small molecules

Synthesis of TTD, 1

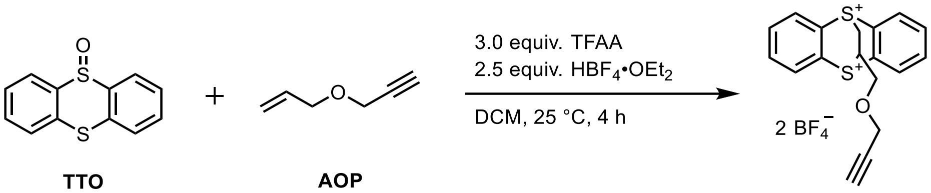

TTO **AOP** **TTD**, **1**, 82 %

Under ambient atmosphere, a 25 mL round-bottom flask was equipped with a Teflon-coated magnetic stirring bar, thianthrene-S-oxide (580 mg, 2.50 mmol, 1.00 equiv.), allyloxy-1-propyne (260 mg, 2.65 mmol, 1.05 equiv.), and 10.0 mL of anhydrous DCM (c = 0.25 M). The flask was capped with a rubber septum, a balloon filled with argon was connected, and the mixture was cooled down to –40 °C in an acetonitrile dry ice bath. Trifluoroacetic anhydride (1.0 mL, 1.6 g, 7.5 mmol, 3.0 equiv.) was added to the stirring (500 rpm) mixture, followed by dropwise (2 drops / second) addition of tetrafluoroboric acid-diethyl ether complex (0.85 mL, 1.0 g, 6.2 mmol, 2.5 equiv.). The mixture was stirred for 30 minutes at –40 °C. Afterwards, the cooling bath was removed and the mixture was stirred for 3.5 hours at 25 °C. The balloon and the rubber septum were removed, the precipitate was collected by filtration, washed with DCM (20 mL), Et_2O (20 mL), and dried under vacuum to give the title compound as a colorless powder (1.00 g, 2.06 mmol, 82.4 %).

NMR Spectroscopy:

^{1}H NMR (600 MHz, CD_3CN, 298 K, δ): 8.58 – 8.50 (m, 4H), 8.15 – 8.07 (m, 4H), 4.82 (dtd, *J* = 8.9, 5.9, 4.0 Hz, 1H), 4.42 (dd, *J* = 13.7, 8.9 Hz, 1H), 4.28 (dd, *J* = 11.4, 4.0 Hz, 1H), 4.14 – 4.04 (m, 2H), 3.83 (dd, *J* = 13.7, 5.8 Hz, 1H), 3.53 (dd, *J* = 11.4, 6.0 Hz, 1H), 2.81 (t, *J* = 2.4 Hz, 1H).

^{13}C NMR (151 MHz, CD_3CN, 298 K, δ): 137.5, 137.1, 137.0, 137.0, 136.9, 136.8, 136.6, 136.3, 126.8, 126.3, 125.4, 124.9, 78.8, 77.9, 66.7, 59.5, 56.2, 40.1.

^{19}F NMR (471 MHz, CD_3CN, 298 K, δ): –151.0.

HRMS-ESI (m/z) calc'd for $C_{18}H_{16}OS_2$ $[M – 2\ BF_4]^{2+}$, 156.0316; found, 156.0316; deviation: 0.0 ppm.

Synthesis of 2

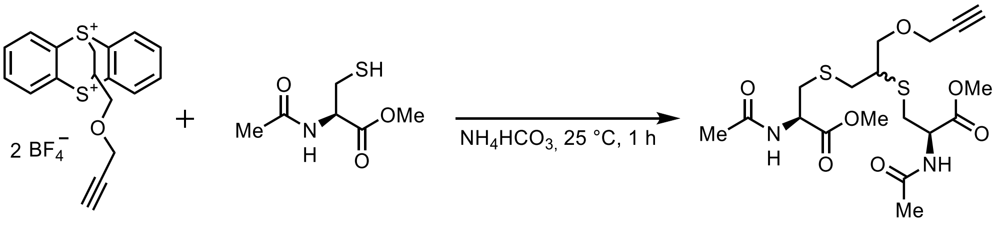

1, 1.0 equiv. **Ac-Cys-OMe**, 2.5 equiv. **2**, 58 %

Under ambient atmosphere, a 100 mL round-bottom flask was equipped with a Teflon-coated magnetic stirring bar and *N*-acetyl-L-Cysteine methyl ester (177 mg, 1.00 mmol, 2.50 equiv.). Then, 40 mL of a freshly prepared ammonium bicarbonate solution (0.25 M) was added, and the mixture was stirred for 2 minutes at 500 rpm at 25 °C. Next, solid **1** (194 mg, 400 µmol, 1.00 equiv.) was added to the flask over 1 minute and the mixture was stirred at 500 rpm at 25 °C for 1 hour. The reaction mixture was filtered, the filtrate was collected, and the filter cake was washed with H_2O (6 x 5 mL). The combined filtrate was lyophilized and the obtained residue was purified by HPLC on a YMC Triart C18 column ((150×30 mm, 5 µm) with a YMC Triart C18 precolumn (50×30mm, 5 µm), flow rate = 42.5 mL/min, 35 °C) with a linear gradient from 20:80 v/v (MeCN : 0.1% v/v TFA in H_2O) to 30:70 (MeCN : 0.1% v/v TFA in H_2O) over 20 minutes. The fractions containing the product (t ≈ 17 min) were collected and lyophilized to afford the title compound as a colorless solid (103 mg, 230 µmol, 57.5 % yield).

NMR Spectroscopy:

^{1}H NMR (600 MHz, DMSO-d$_6$, 298 K, δ): 8.40 – 8.35 (m, 2H), 4.47 – 4.40 (m, 2H), 4.15 (t, J = 2.5 Hz, 2H), 3.68 – 3.62 (m, 7H), 3.60 – 3.55 (m, 1H), 3.45 – 3.42 (m, 1H), 3.08 – 3.01 (m, 1H), 3.00 – 2.89 (m, 2H), 2.86 – 2.72 (m, 4H), 1.86 (s, 6H).

^{13}C NMR (151 MHz, DMSO-d$_6$, 298 K, δ): 171.3, 171.2, 171.2, 169.5, 169.5, 169.4, 80.0, 80.0, 77.5, 77.4, 70.7, 70.6, 57.7, 57.7, 52.4, 52.4, 52.2, 52.1, 45.3, 45.2, 40.1, 39.9, 39.8, 39.7, 39.4, 39.2, 39.1, 34.5, 34.3, 33.7, 33.6, 32.2, 22.3.

HRMS-ESI (m/z) calc'd for $C_{18}H_{28}N_2O_7S_2Na$ $[M+Na]^+$, 471.1230; found, 471.1231; deviation: 0.2 ppm

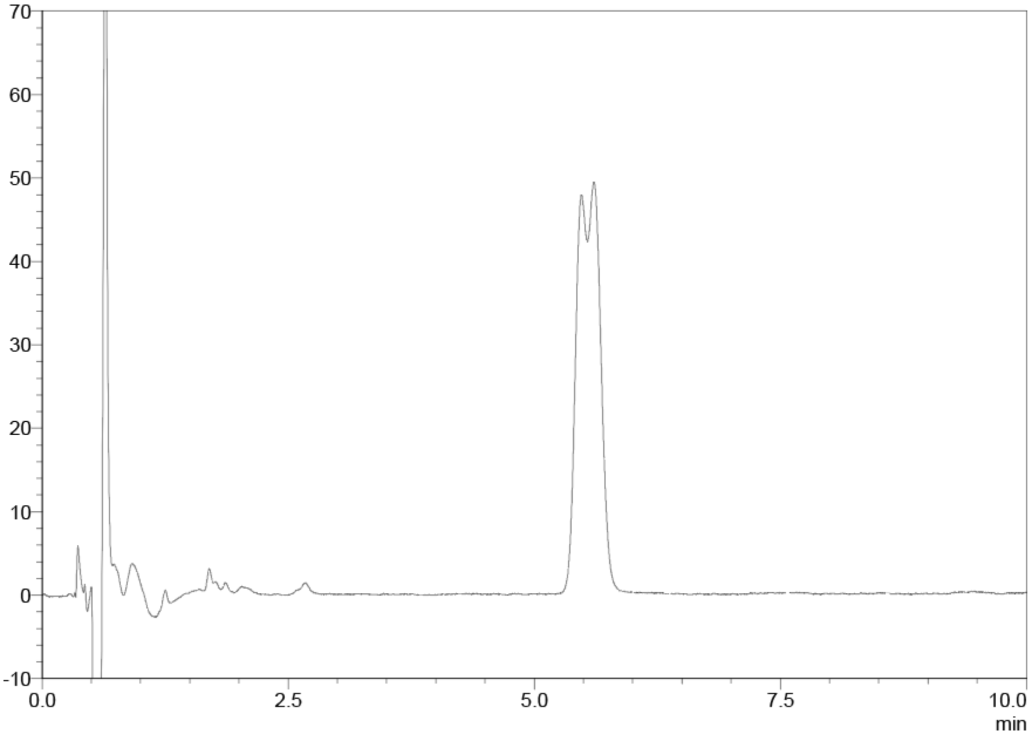

*Figure S1. Analytical (UV-Vis) HPLC trace of **2** (YMC-Triart C18 column, 50 x 3.0 mm, 1.9 µm, flow rate = 0.5 mL/min), eluent: 20:80 v/v MeCN : 0.1% v/v TFA in H_2O.*

Stability of 1 under ambient atmosphere

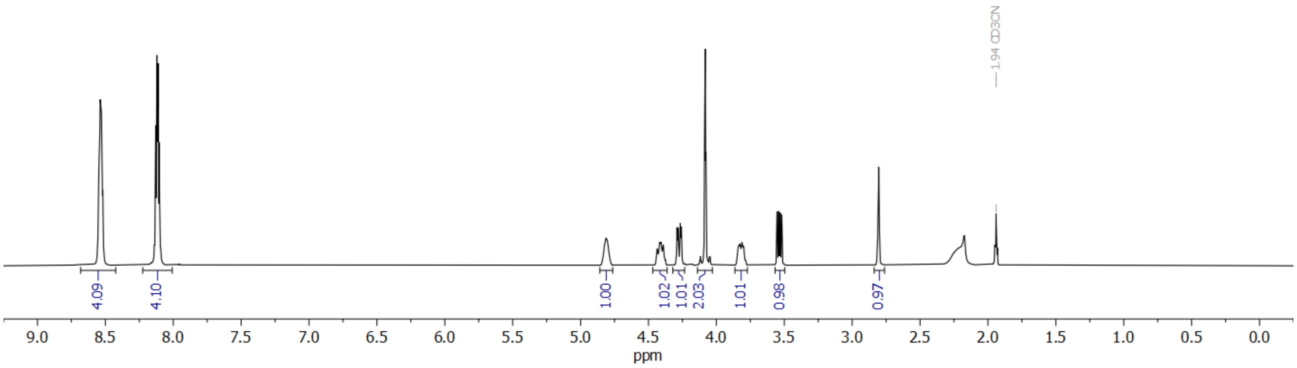

Figure S2. ^{1}*H NMR of* ***1***.

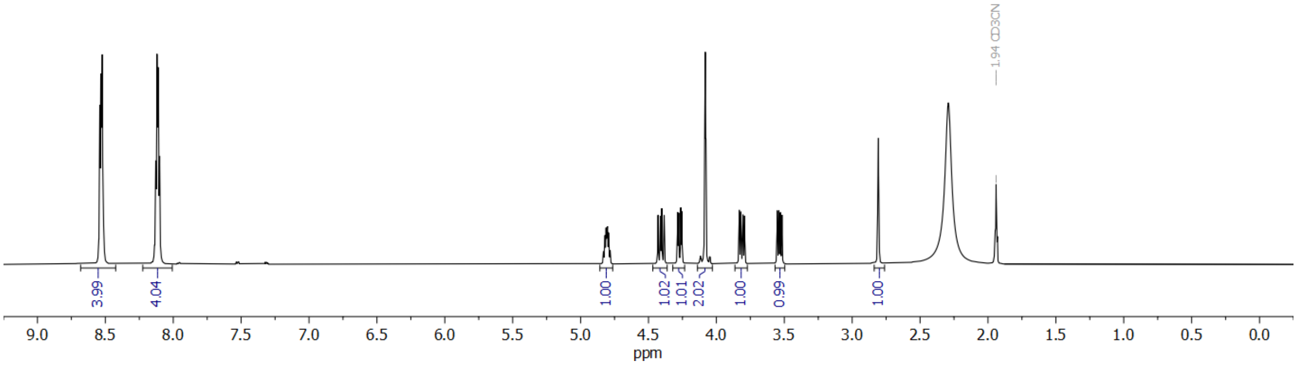

Figure S3. ^{1}*H NMR of* ***1*** *stored for two years at 20–25 °C under ambient atmosphere.*

Deprotonation of 1 in phosphate buffer

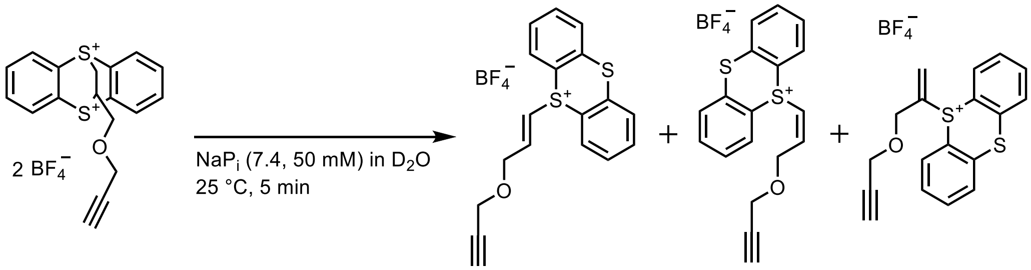

1 **1e** **1z** **1t**

At ambient temperature, a NaP$_i$ solution (50 µL, pH 7.4, 0.5 M) in D_2O was mixed with 400 µL of D_2O in a 1.5 mL tube. Then, a freshly prepared solution of **1** in D_2O (50 µL, 50 mM) was added to the tube, the mixture was vortexed for 10 seconds, and NMR spectra were acquired.

The product ratios were determined by integration of characteristic ^{1}H signals that were sufficiently resolved and the respective integral values were averaged. The resulting integral ratios are 4.4 (**1e**) : 1.4 (**1z**) : 1.0 (**1t**).

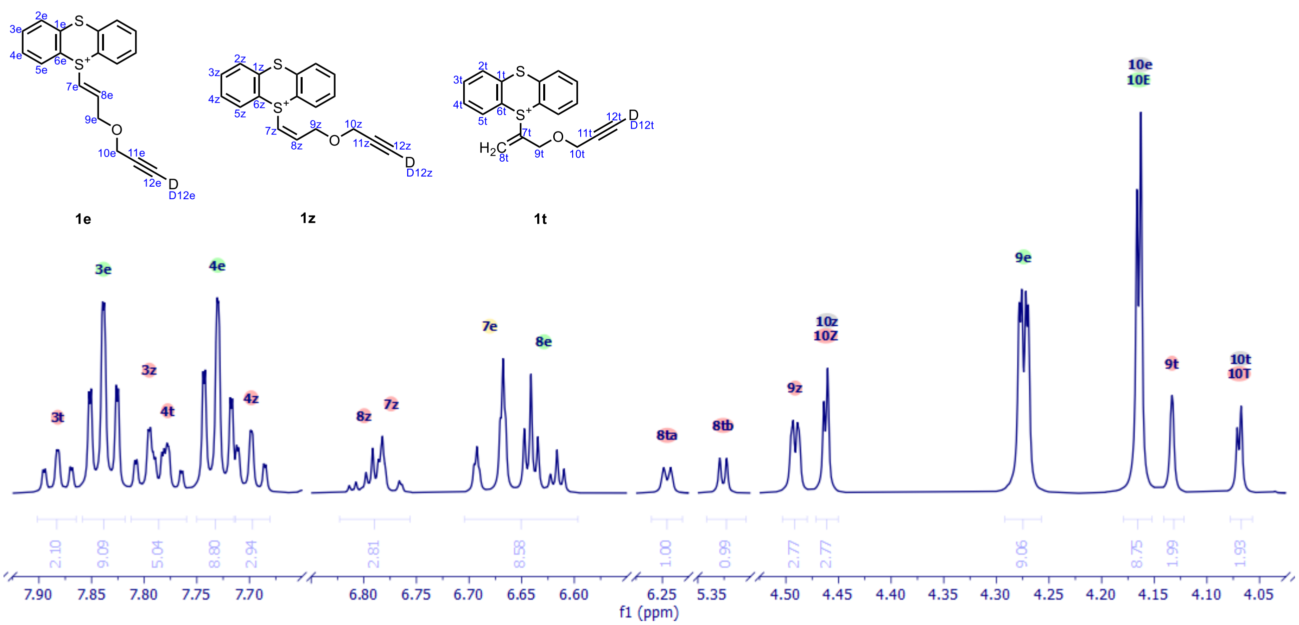

Figure S4. ^{1}H NMR spectrum of the peak areas used for the quantification of the product ratio. For details, see [full NMR characterization](#).

Deprotonation half-life of TTD in NaP$_i$

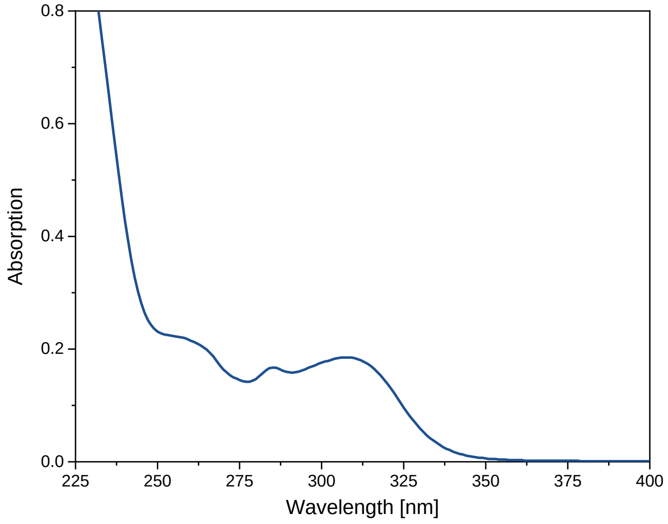

*Figure S5. Absorbance spectrum of **TTD** (50 µM) in water.*

The consumption of **TTD** over time was determined by measuring the absorbance change of **TTD** at 307 nm. Sodium phosphate buffer (pH 7.4, c = 50 mM) containing 0.140 M NaCl was used as solvent. The NaP$_i$ (1990 µL) stock solution was pipetted into a quartz cuvette charged with a Teflon-coated magnetic stirring bar. The cuvette was thermostated for 5 minutes at (25 ± 0.1) °C in the cell holder. After performing the background measurement, a stock solution of **TTD** in H_2O (10.0 µL, c = 10.0 mM) was added to the cuvette. The reaction mixture was homogenized by pipetting the mixture up and down. Then, the absorbance of **TTD** at 307 nm was measured while stirring. The first order rate constant for the deprotonation of **TTD** in buffer was determined by plotting ln[ΔAbs_{307nm}] against time and determining the slope of the linear fit for each graph.

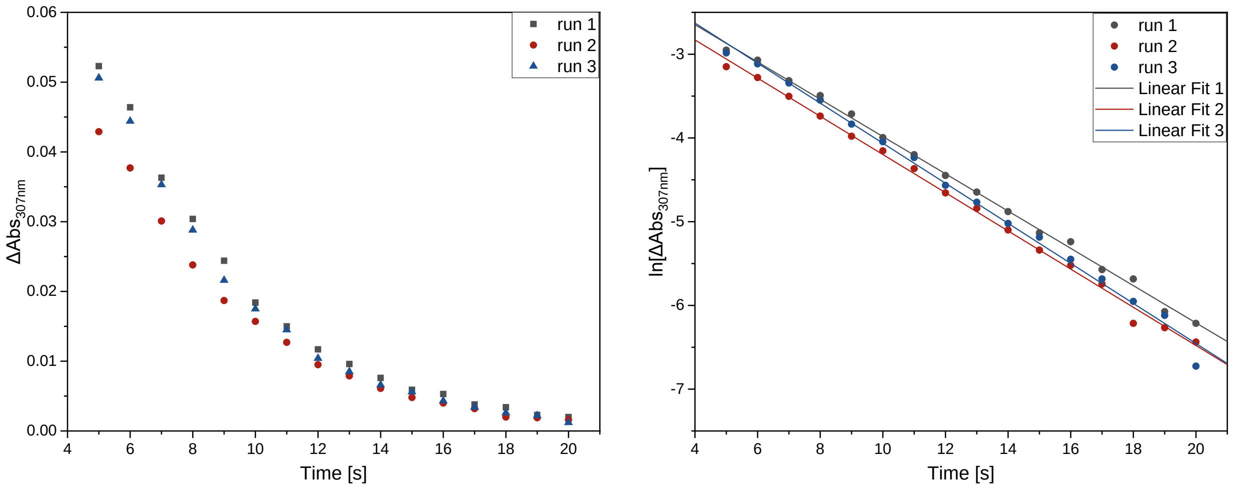

*Figure S6. Absorbance decay of **TTD** (50 μM) in NaP$_i$.*

Run	k_1, s^{-1}	$t_{1/2}$, s	R^2
1	0.223 ± 0.003	3.1	0.998
2	0.228 ± 0.004	3.0	0.996
3	0.239 ± 0.005	2.9	0.994

Based on the data from three runs: k_1(**TTD**) = 0.23 ± 0.005 s^{-1} and $t_{1/2}$ = ln2 / k_1 = 3.0 ± 0.1 s.

Calculated TPSA and logP

The TPSA and logP values for **1e**, **1z**, **1t**, and **VTT** were calculated using SwissADME[4].

Molecule	Canonical SMILES	*TPSA*	*XLOGP3*	WLOGP	MLOGP	Silicos-IT Log P	Consensus Log P
**VTT**	C=C[S+]1c2ccccc2Sc2c1cccc2	*50.6*	*4.64*	4.33	5.01	3.33	3.46
**1e**	C#CCOC/C=C/[S+]1c2ccccc2Sc2c1cccc2	*59.8*	*4.28*	4.43	4.85	3.97	3.51
**1z**	C#CCOC/C=C\[S+]1c2ccccc2Sc2c1cccc2	*59.8*	*4.28*	4.43	4.85	3.97	3.51
**1t**	C#CCOCC(=C)[S+]1c2ccccc2Sc2c1cccc2	*59.8*	*4.42*	4.43	4.85	3.98	3.54

Deuterium incorporation studies

Product characterization using NMR

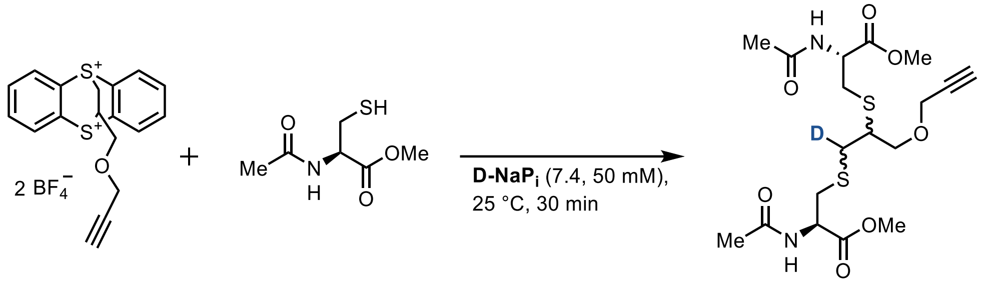

1, 1.0 equiv. **Ac-Cys-OMe**, 2.5 equiv. **2-D**

The reaction between **TTD** and Ac-Cys-OMe was performed in deuterated buffer (D-NaP$_i$, pH 7.4, c = 50 mM) and the reaction mixture was analyzed with NMR using 3-(trimethylsilyl)propionic-2,2,3,3-d$_4$ acid sodium salt (**TSP**) as an internal standard:

NaP$_i$ buffer in D_2O (50 µL, pH 7.4, c = 0.5 M), D_2O (295 µL), a freshly prepared stock solution of Ac-Cys-OMe in D_2O (100 µL, c = 50 mM), a stock solution of TSP in D_2O (50 µL, c = 50 mM) were pipetted into an Eppendorf tube and mixed by pipetting the mixture up and down. Subsequently, a freshly prepared stock solution of **TTD** in DMF-d$_7$ (5.0 µL, c = 0.50 M) was added to the tube to obtain a mixture containing **TTD** (5.0 mM, 1.0 equiv.), Ac-Cys-OMe (10 mM, 2.0 equiv.), **TSP** (5.0 mM, 1.0 equiv.) in D-NaP$_i$ (50 mM). The reaction mixture was vortexed for 15 seconds, incubated at 25 °C for 30 minutes at 600 rpm, and centrifuged for 5 minutes at 17,000 g. The obtained supernatant was used for the NMR measurements.

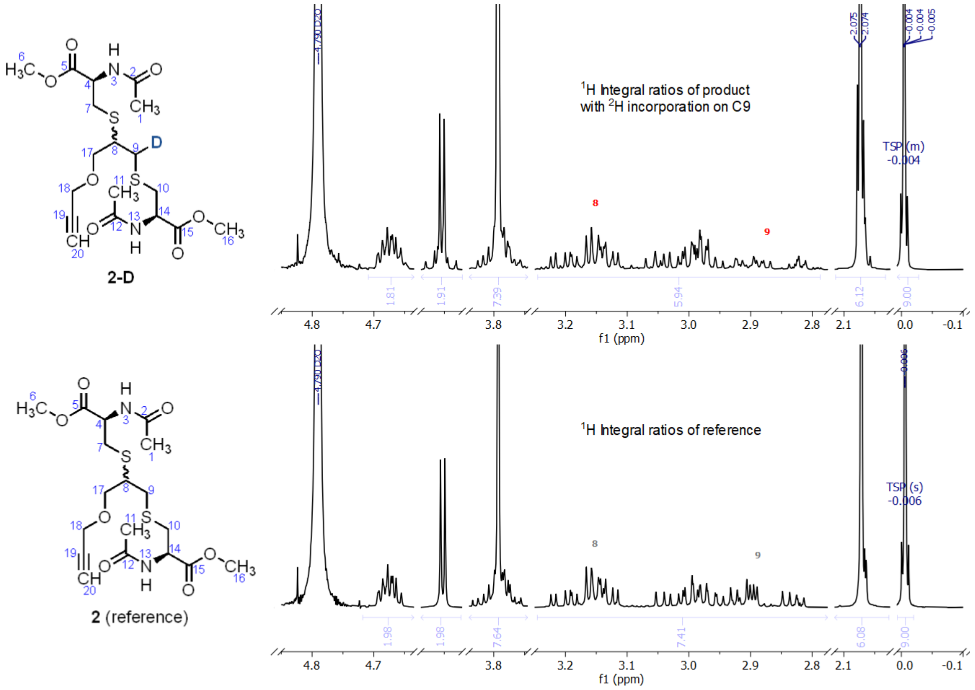

*Figure S7. Top: integrated ^{1}H NMR spectrum of the mixture containing **TTD** (5.0 mM), Ac-Cys-OMe (10 mM), and **TSP** (5.0 mM) in D-NaP$_i$ (pH 7.4, 50 mM).*
*Bottom: integrated ^{1}H NMR spectrum of the mixture containing **2** (5.0 mM) and **TSP** (5.0 mM) in D-NaP$_i$ (pH 7.4, 50 mM).*

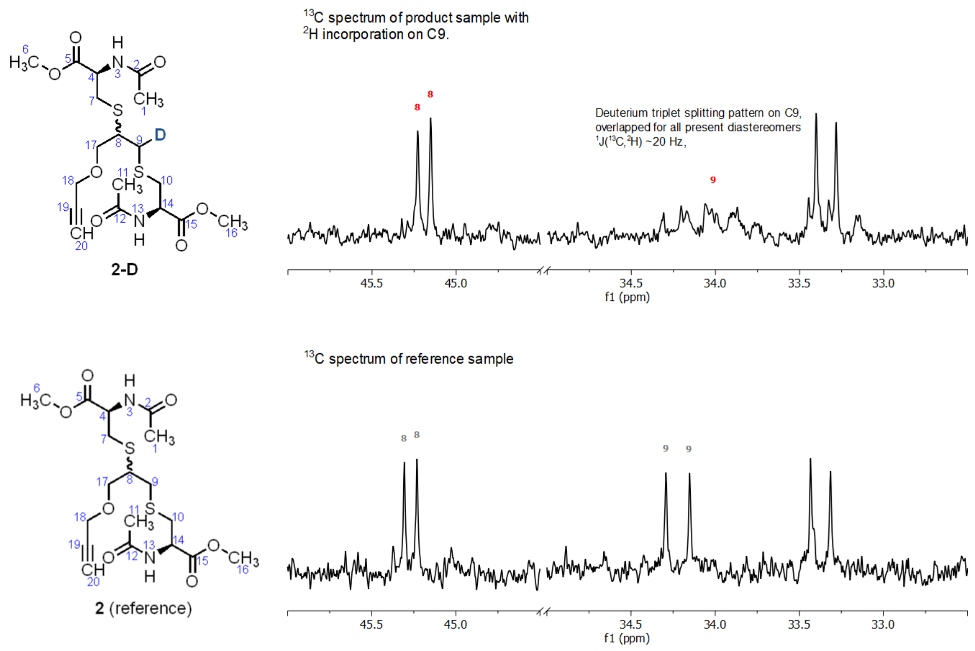

*Figure S8. Top: ^{13}C NMR spectrum of the mixture containing **TTD** (5.0 mM), Ac-Cys-OMe (10 mM), and **TSP** (5.0 mM) in D-NaP$_i$ (pH 7.4, 50 mM).*
*Bottom: ^{13}C NMR spectrum of the mixture containing **2** (5.0 mM) and **TSP** (5.0 mM) in D-NaP$_i$ (pH 7.4, 50 mM).*

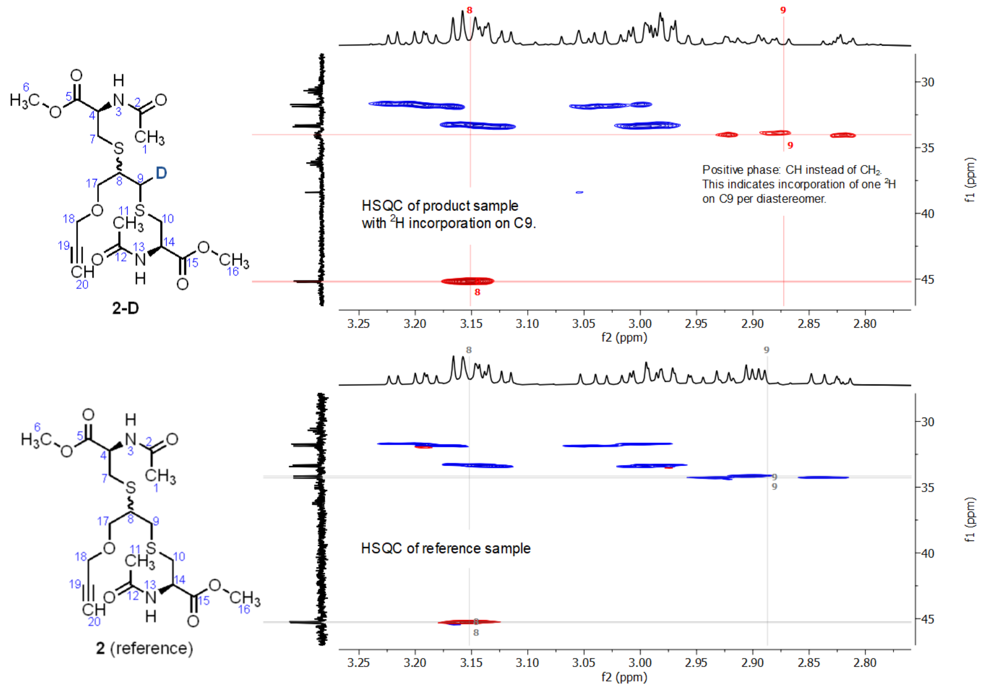

*Figure S9. Top: HSQC NMR spectrum of the mixture containing **TTD** (5.0 mM), Ac-Cys-OMe (10 mM), and **TSP** (5.0 mM) in D-NaP$_i$ (pH 7.4, 50 mM).*
*Bottom: HSQC NMR spectrum of the mixture containing **2** (5.0 mM) and **TSP** (5.0 mM) in D-NaP$_i$ (pH 7.4, 50 mM).*

Product characterization using MS

NaP_i buffer (50 µL, pH 7.4, c = 0.5 M), H_2O (320 µL), and a freshly prepared stock solution of Ac-Cys-OMe in H_2O (125 µL, c = 50 mM) were pipetted into an Eppendorf tube and mixed by pipetting the mixture up and down. Subsequently, a freshly prepared stock solution of **TTD** in DMF (5.0 µL, c = 0.50 M) was added to the tube to obtain a mixture containing **TTD** (5.0 mM, 1.0 equiv.) and Ac-Cys-OMe (13 mM, 2.5 equiv.) in NaP_i (50 mM). The reaction mixture was vortexed for 15 seconds, incubated at 25 °C for 30 minutes at 600 rpm, and centrifuged for 5 minutes at 17,000 xg. The obtained supernatant was used for the HRMS-measurement.

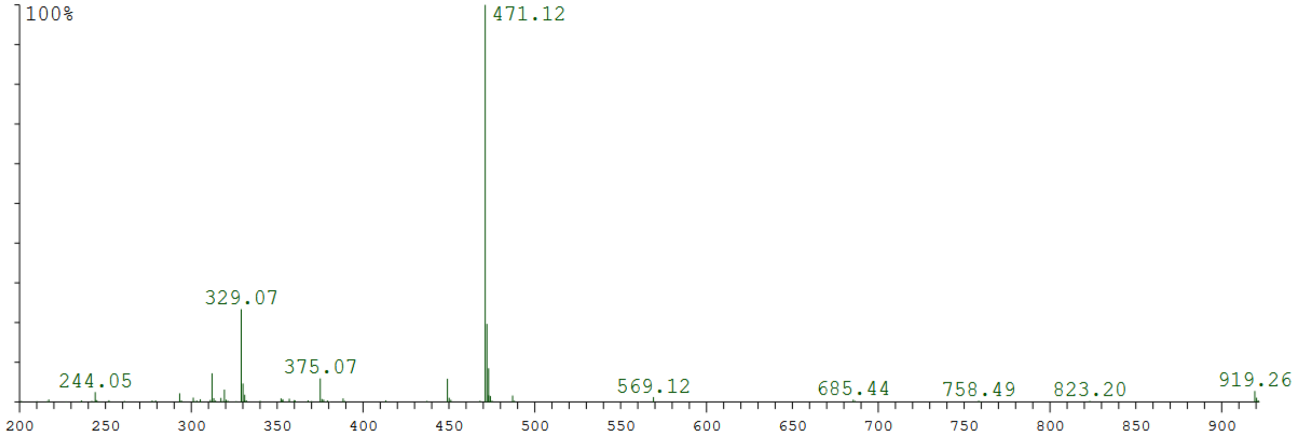

*Figure S10. HRMS analysis of the mixture containing 5.0 mM **TTD** and 13 mM Ac-Cys-OMe in NaP_i (pH 7.4, 50 mM). Calculated mass for $C_{18}H_{28}N_2O_7S_2$, $[M+Na]^+$: 471.1230; detected: 471.1229. Deviation: –0.2 ppm.*

NaP_i buffer in D_2O (50 µL, pH 7.4, c = 0.5 M), D_2O (320 µL), and a freshly prepared stock solution of Ac-Cys-OMe in D_2O (125 µL, c = 50 mM) were pipetted into an Eppendorf tube and mixed by pipetting the mixture up and down. Subsequently, a freshly prepared stock solution of **TTD** in DMF-d_7 (5.0 µL, c = 0.50 M) was added to the tube to obtain a mixture containing **TTD** (5.0 mM, 1.0 equiv.) and Ac-Cys-OMe (13 mM, 2.5 equiv.) in D-NaP_i (50 mM). The reaction mixture was vortexed for 15 seconds, incubated at 25 °C for 30 minutes at 600 rpm, and centrifuged for 5 minutes at 17,000 xg. The obtained supernatant was used for the HRMS-measurement.

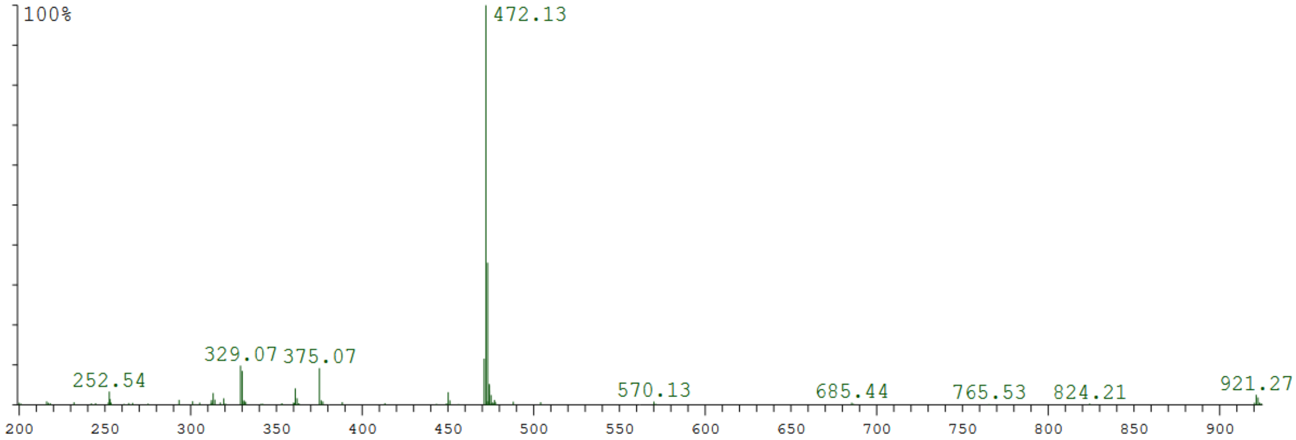

*Figure S11. HRMS analysis of the mixture containing 5.0 mM **TTD** and 13 mM Ac-Cys-OMe in D-NaP_i (pH 7.4, 50 mM). Calculated mass for $C_{18}H_{27}DN_2O_7S_2$, $[M+Na]^+$: 472.1293; detected: 472.1292. Deviation: –0.2 ppm.*

Reactivity with proteins

Characterization of protein starting materials

Ubiquitin was purchased from Sigma Aldrich (#93950).

Ubiquitin K63C was recombinantly expressed and purified as described previously[5], using a construct generated by subcloning Ubiquitin K63C from a plasmid obtained from Addgene (pMAL-c5X, Plasmid #86589). The gene was amplified using primers 5'-AAAAGCTAGCCAGATCTTCGTCAAGAC-GTTAAC-3' and 5'-TTTTAAGCTTTTAACCACCTCTTAGTCTTAAGACAAG-3' and inserted into pET28a via NheI and HindIII restriction sites. Protein expression in *E. coli* NiCo21 (DE3), Ni-NTA purification, dialysis and thrombin cleavage for removal of the His-tag followed the protocol described previously[5]. The protein mutant sequence: GSHMASQIFVKTLTGKTITLEVEPSDTIENVKAKIQDKEGIPPDQQRLIFAGKQLEDGRTLSDYNIQCESTLHLV LRLRGG*

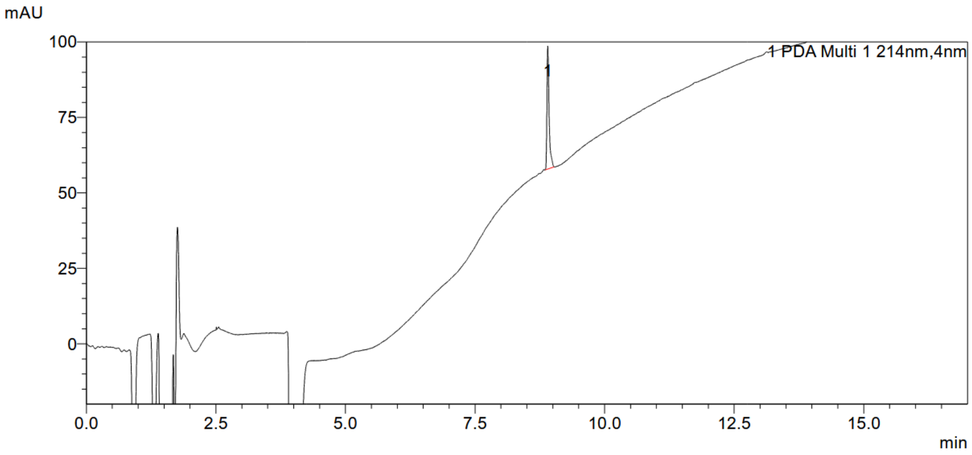

Figure S12. LC−MS analysis of Ubiquitin K63C pre-reduced with 20 equiv. TCEP: UV trace.

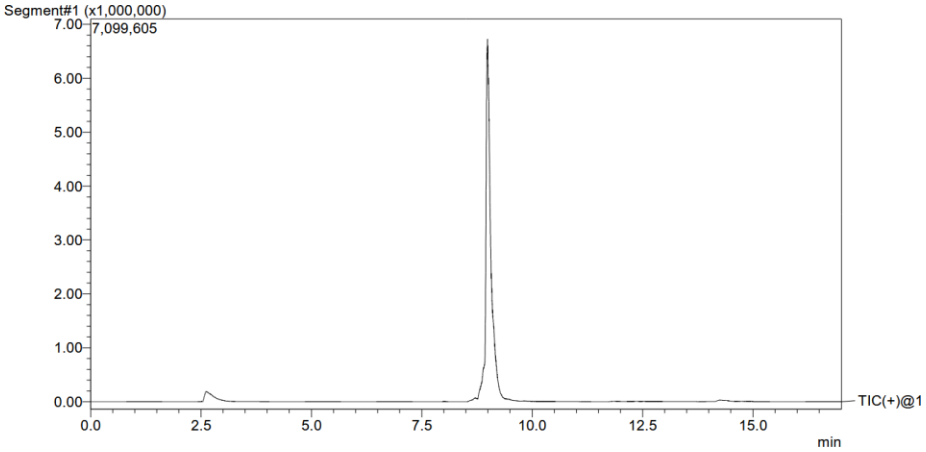

Figure S13. LC−MS analysis of Ubiquitin K63C pre-reduced with 20 equiv. TCEP: total ion chromatogram.

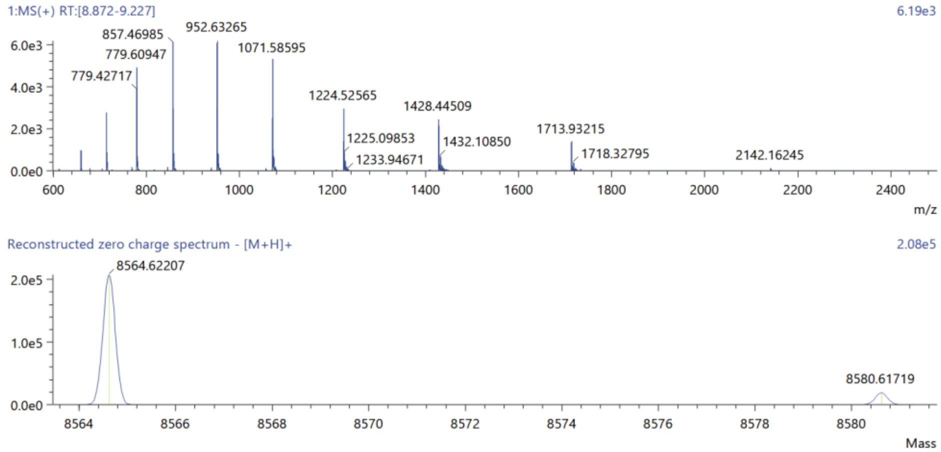

Figure S14. LC−MS analysis of Ubiquitin K63C pre-reduced with 20 equiv. TCEP: ion series and deconvoluted spectrum of the protein peak; calculated mass: Ubiquitin, 8564.751 Da. Observed masses: 8564.622 Da, 8580.617 Da.

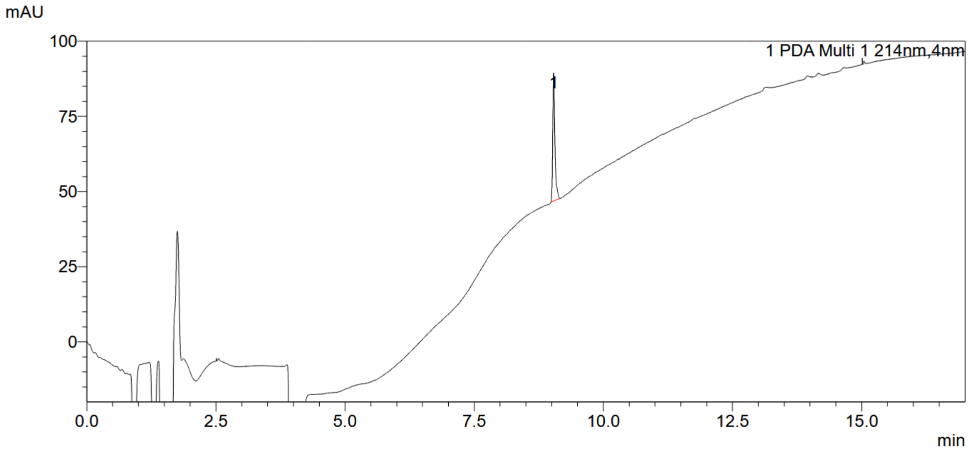

Figure S15. LC−MS analysis of Ubiquitin K63C pre-reduced with 20 equiv. TCEP: UV trace.

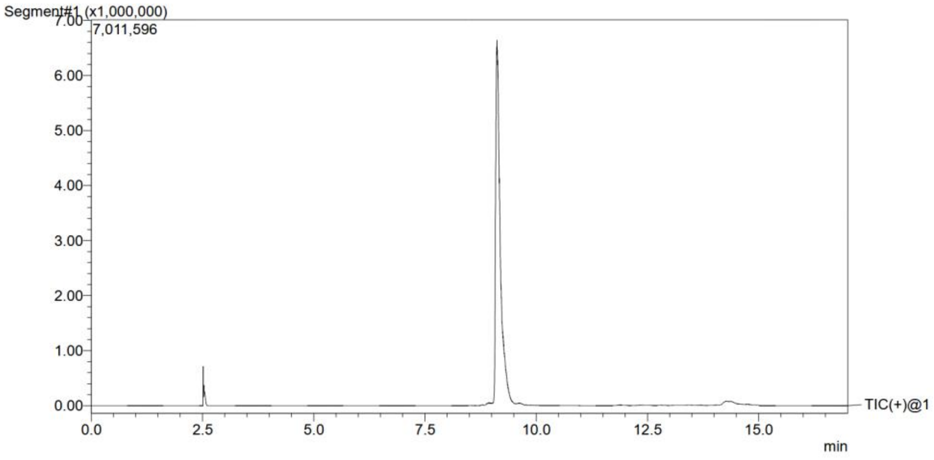

Figure S16. LC−MS analysis of Ubiquitin K63C pre-reduced with 20 equiv. TCEP: total ion chromatogram.

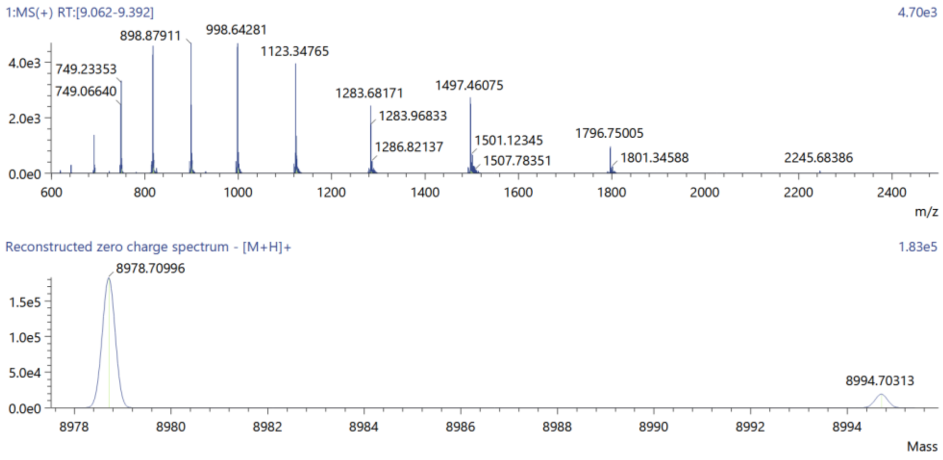

Figure S17. LC−MS analysis of Ubiquitin K63C pre-reduced with 20 equiv. TCEP: ion series and deconvoluted spectrum of the protein peak; calculated mass: Ubiquitin K63C, 8979.1454 Da. Observed masses: 8978.710 Da, 8994.703 Da.

Reactions with Ubiquitin K63C

Reaction with TTD

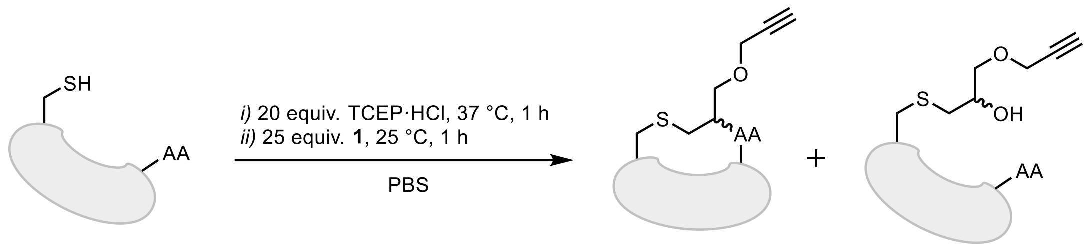

At 20–25 °C, 6.0 μL of Ubiquitin K63C (2.99 mg/mL, 1.0 equiv.) in NaP_i buffer (pH 7.0, c = 50 mM) was added to a 1.5 mL Eppendorf tube. Next, 92 μL phosphate-buffered saline (PBS) was added. Then, 1.0 μL of a TCEP stock solution (40 mM, 20 equiv.) in H_2O was added. The mixture was vortexed for 5 seconds, transferred into a Thermocycler pre-heated at 37 °C, and incubated at 37 °C for 1 hour at 600 rpm. Next, 1.0 μL of a freshly prepared stock solution of **1** in DMSO (50 mM, 25 equiv.) was added to the mixture. The reaction mixture was vortexed for 5 seconds, transferred into a Thermocycler pre-heated at 25 °C, and incubated at 25 °C at 600 rpm for 1 hour. A stock solution of β-mercaptoethanol (1.0 μL, 50 mM, 25 equiv.) in H_2O was added, the mixture was incubated for 15 minutes at 25 °C, centrifuged for 3 minutes at 17,000 xg, and the supernatant was analyzed using LC–MS.

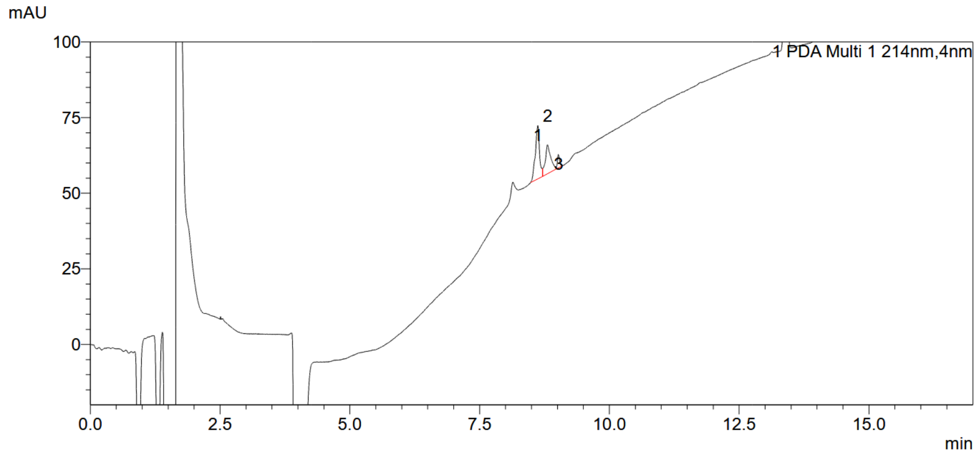

Figure S18. LC−MS analysis of the reaction mixture with Ubiquitin K63C: UV trace. Relative calculated areas: peak 1: 55 %, peak 2: 38 %, peak 3: 7 %.

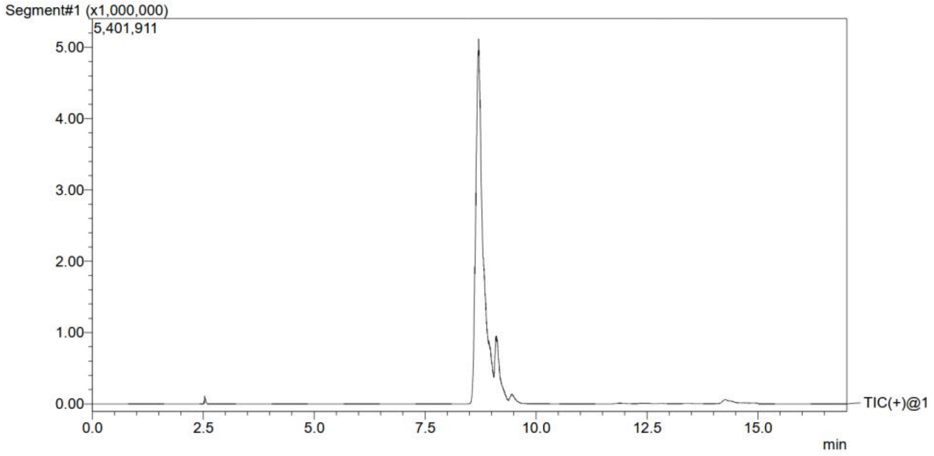

Figure S19. LC−MS analysis of the reaction mixture with Ubiquitin K63C: total ion chromatogram.

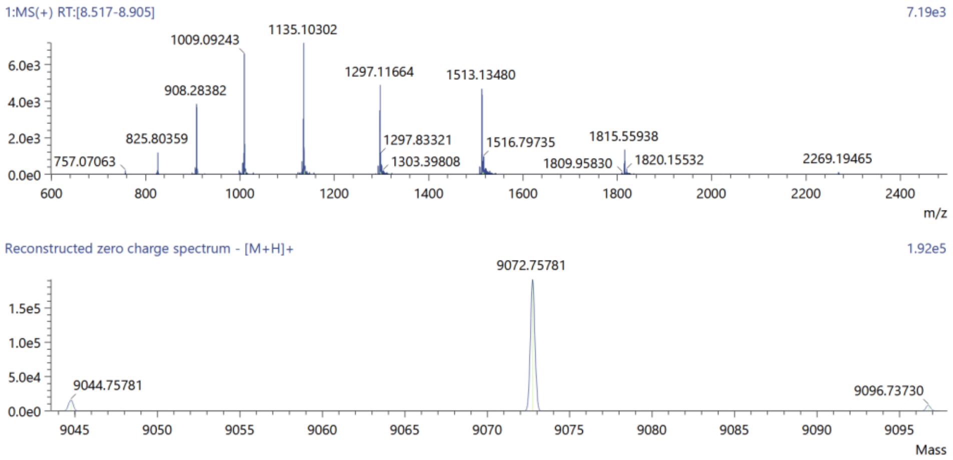

*Figure S20. LC−MS analysis of the reaction mixture with Ubiquitin K63C: ion series and deconvoluted spectrum of protein peak 1; calculated mass: **S1**, 9073.187 Da. Observed masses: 9072.758 Da, 9044.758 Da, 9096.737 Da.*

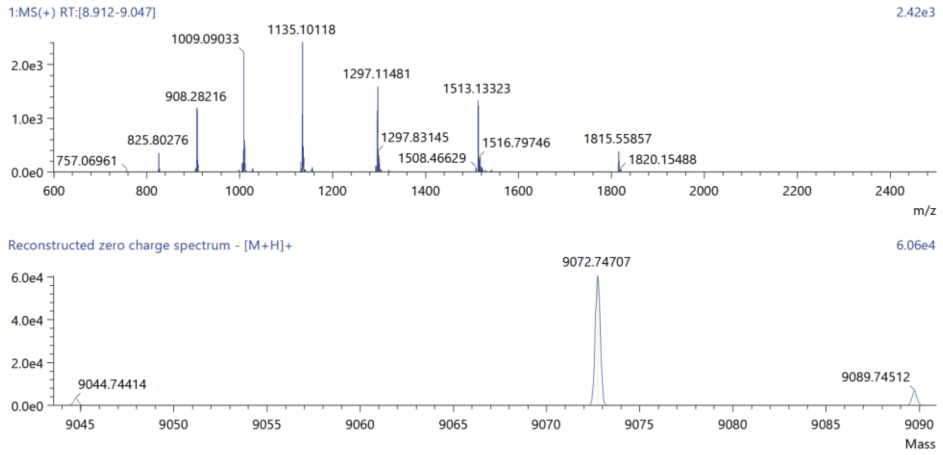

Figure S21. LC−MS analysis of the reaction mixture with Ubiquitin K63C: ion series and deconvoluted spectrum of protein peak 2; calculated mass: ***S1****, 9073.187 Da. Observed masses: 9072.747 Da, 9044.744 Da, 9089.745 Da.*

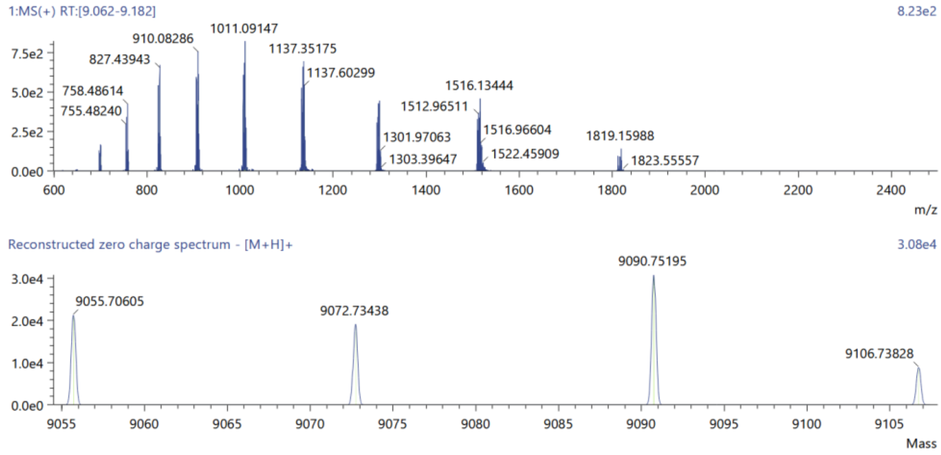

Figure S22. LC−MS analysis of the reaction mixture with Ubiquitin K63C: ion series and deconvoluted spectrum of protein peak 3; calculated mass: ***S1****, 9073.187 Da;* ***S2****, 9091.198 Da. Observed masses: 9055.706 Da, 9072.734 Da, 9090.752 Da, 9106.738 Da.*

Reaction with VTT

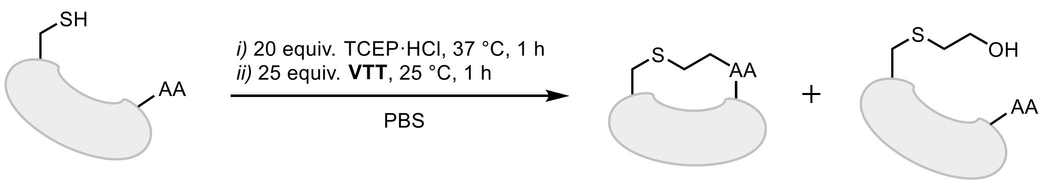

Ubiquitin K63C **S3** **S4**

At 20–25 °C, 6.0 µL of Ubiquitin K63C (2.99 mg/mL, 1.0 equiv.) in NaP_i buffer (pH 7.0, c = 50 mM) was added to a 1.5 mL Eppendorf tube. Next, 92 µL phosphate-buffered saline (PBS) was added. Then, 1.0 µL of a TCEP stock solution (40 mM, 20 equiv.) in H_2O was added. The mixture was vortexed for 5 seconds, transferred into a Thermocycler pre-heated at 37 °C, and incubated at 37 °C for 1 hour at 600 rpm. Next, 1.0 µL of a freshly prepared stock solution of **VTT** in DMSO (50 mM, 25 equiv.) was added to the mixture. The reaction mixture was vortexed for 5 seconds, transferred into a Thermocycler pre-heated at 25 °C, and incubated at 25 °C at

600 rpm for 1 hour. A stock solution of β-mercaptoethanol (1.0 µL, 50 mM, 25 equiv.) in H_2O was added, the mixture was incubated for 15 minutes at 25 °C, centrifuged for 3 minutes at 17,000 xg, and the supernatant was analyzed using LC–MS.

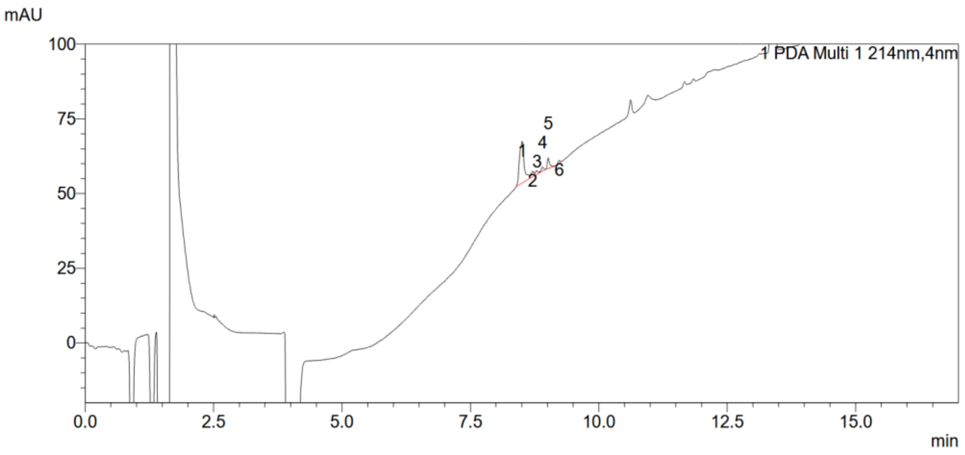

Figure S23. LC−MS analysis of the reaction mixture with Ubiquitin K63C: UV trace. Relative calculated areas: peak 1: 74 %, peak 2: 7 %, peak 3: 4 %, peak 4: 3 %, peak 5: 10 %, peak 6: 2 %.

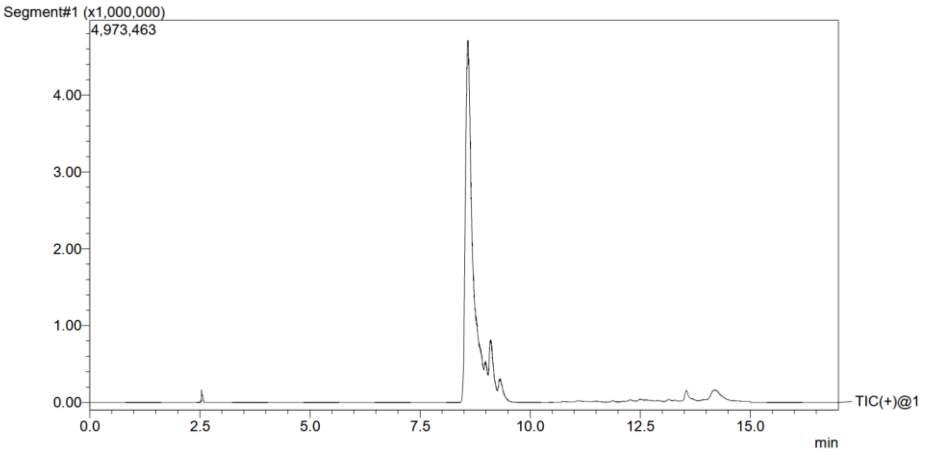

Figure S24. LC−MS analysis of the reaction mixture with Ubiquitin K63C: total ion chromatogram.

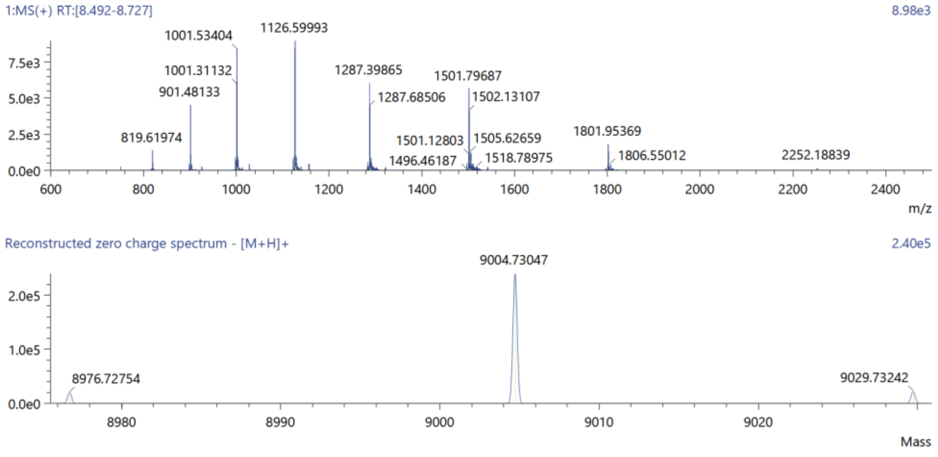

*Figure S25. LC−MS analysis of the reaction mixture with Ubiquitin K63C: ion series and deconvoluted spectrum of protein peak 1; calculated mass: **S3**, 9005.161 Da. Observed masses: 9004.730 Da, 8976.727 Da, 9029.732 Da.*

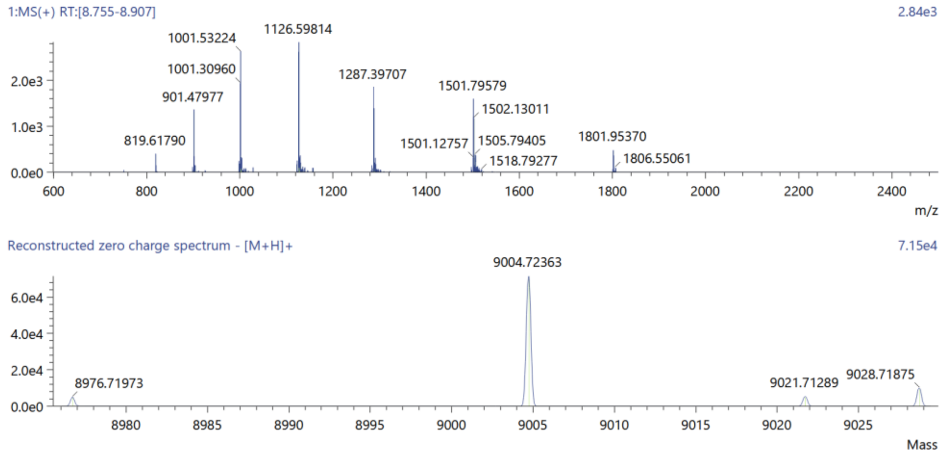

*Figure S26. LC−MS analysis of the reaction mixture with Ubiquitin K63C: ion series and deconvoluted spectrum of protein peaks 2+3; calculated mass: **S3**, 9005.161 Da; **S4**, 9023.172 Da. Observed masses: 9004.724 Da, 8976.720 Da, 9021.713 Da, 9028.719 Da.*

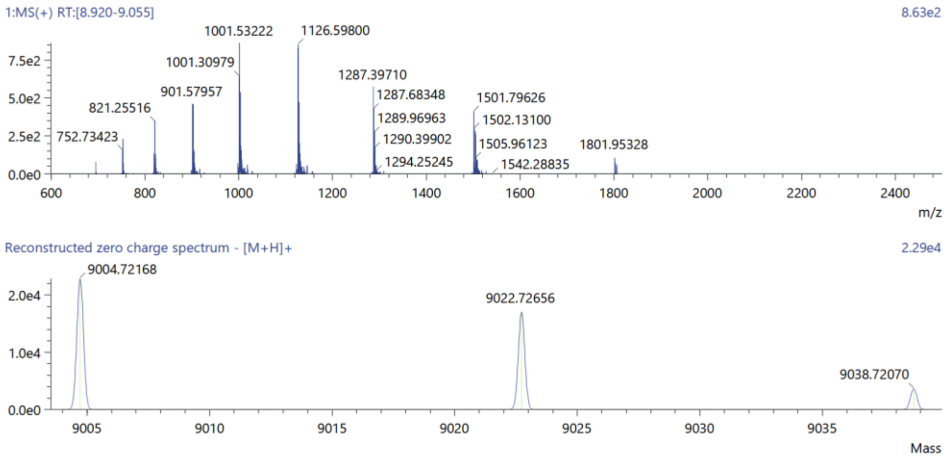

*Figure S27. LC−MS analysis of the reaction mixture with Ubiquitin K63C: ion series and deconvoluted spectrum of protein peak 4; calculated mass: **S3**, 9005.161 Da; **S4**, 9023.172 Da. Observed masses: 9004.722 Da, 9022.727 Da, 9038.721 Da.*

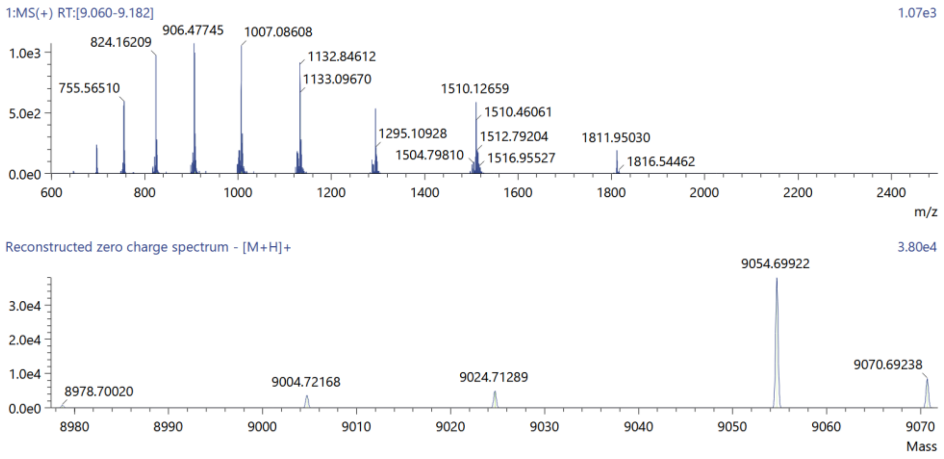

*Figure S28. LC−MS analysis of the reaction mixture with Ubiquitin K63C: ion series and deconvoluted spectrum of protein peak 5; calculated mass: **S3**, 9005.161 Da; **S4**, 9023.172 Da. Observed masses: 9054.699 Da, 8978.700 Da, 9004.722 Da, 9024.713 Da, 9070.692 Da.*

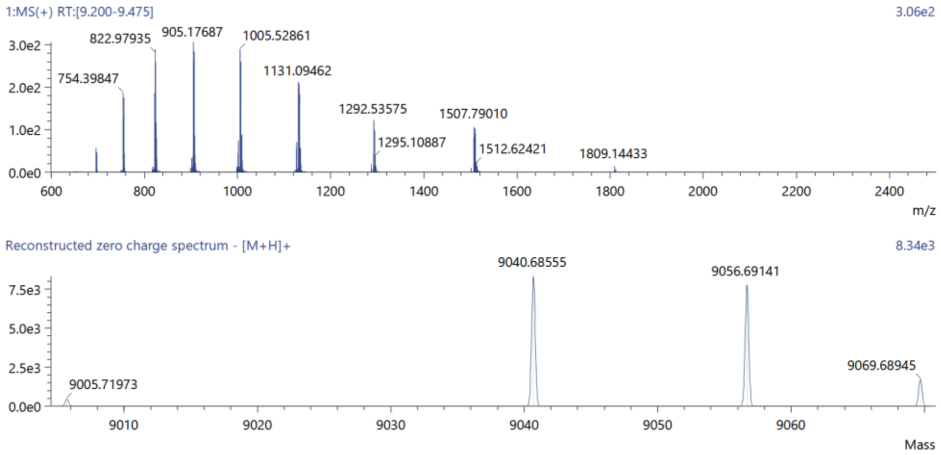

*Figure S29. LC−MS analysis of the reaction mixture with Ubiquitin K63C: ion series and deconvoluted spectrum of protein peak 6; calculated mass: **S3**, 9005.161 Da. Observed masses: 9040.686 Da, 9056.691 Da, 9005.720 Da, 9069.689 Da.*

Reaction with dicationic VTT precursor

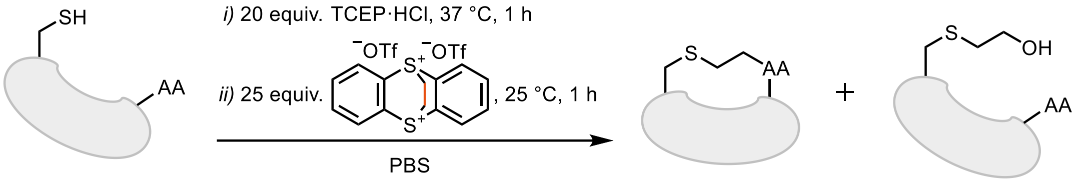

At 20–25 °C, 6.0 µL of Ubiquitin K63C (2.99 mg/mL, 1.0 equiv.) in NaP$_i$ buffer (pH 7.0, c = 50 mM) was added to a 1.5 mL Eppendorf tube. Next, 92 µL phosphate-buffered saline (PBS) was added. Then, 1.0 µL of a TCEP stock solution (40 mM, 20 equiv.) in H_2O was added. The mixture was vortexed for 5 seconds, transferred into a Thermocycler pre-heated at 37 °C, and incubated at 37 °C for 1 hour at 600 rpm. Next, 1.0 µL of a freshly prepared stock solution of the cyclic **VTT** precursor in DMSO (50 mM, 25 equiv.) was added to the mixture. The reaction mixture was vortexed for 5 seconds, transferred into a Thermocycler pre-heated at 25 °C, and incubated at 25 °C at 600 rpm for 1 hour. A stock solution of β-mercaptoethanol (1.0 µL, 50 mM, 25 equiv.) in H_2O was added, the mixture was incubated for 15 minutes at 25 °C, centrifuged for 3 minutes at 17,000 xg, and the supernatant was analyzed using LC–MS.

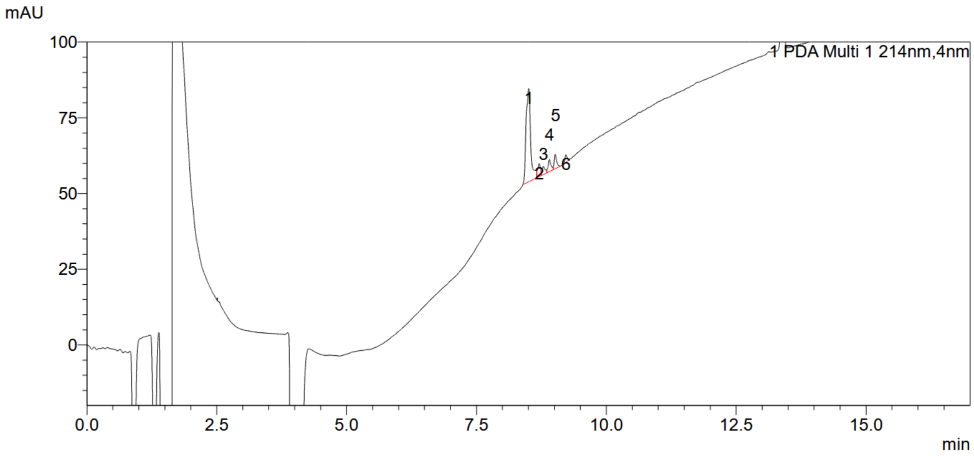

Figure S30. LC−MS analysis of the reaction mixture with Ubiquitin K63C: UV trace. Relative calculated areas: peak 1: 74 %, peak 2: 6 %, peak 3: 5 %, peak 4: 6 %, peak 5: 6 %, peak 6: 3 %.

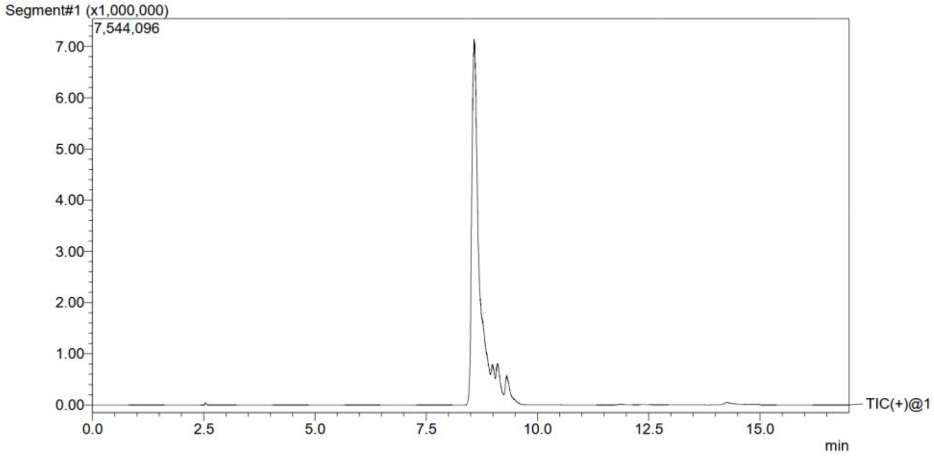

Figure S31. LC−MS analysis of the reaction mixture with Ubiquitin K63C: total ion chromatogram.

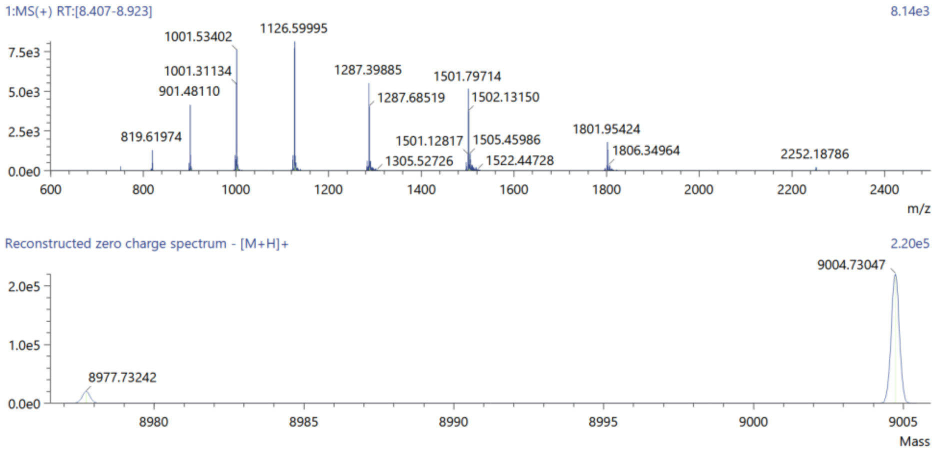

Figure S32. LC−MS analysis of the reaction mixture with Ubiquitin K63C: ion series and deconvoluted spectrum of protein peaks 1,2,3; calculated mass: ***S3****, 9005.161 Da. Observed masses: 9004.730 Da, 8977.732 Da.*

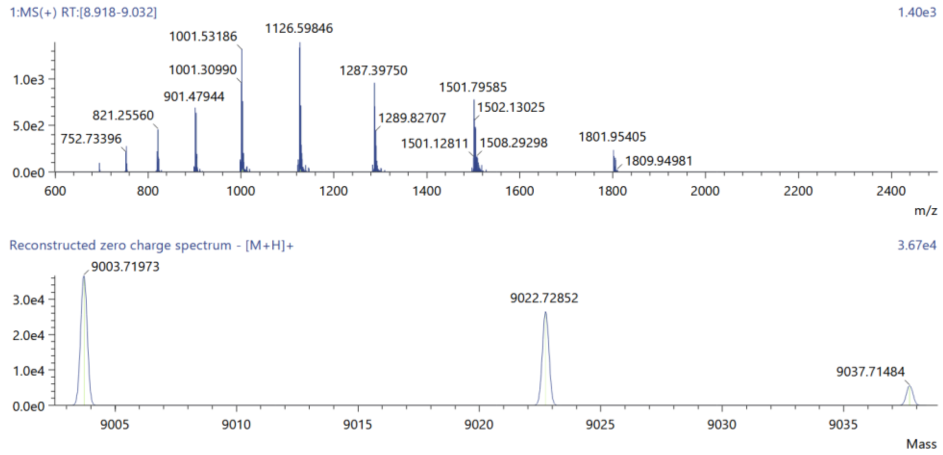

Figure S33. LC−MS analysis of the reaction mixture with Ubiquitin K63C: ion series and deconvoluted spectrum of protein peak 4; calculated mass: ***S3****, 9005.161 Da;* ***S4****, 9023.172 Da. Observed masses: 9003.720 Da, 9022.729 Da, 9037.715 Da.*

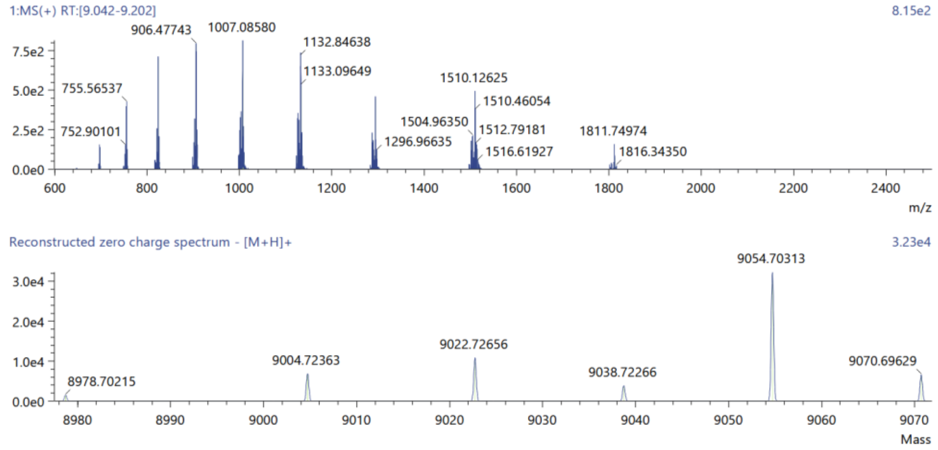

Figure S34. LC−MS analysis of the reaction mixture with Ubiquitin K63C: ion series and deconvoluted spectrum of protein peak 5; calculated mass: ***S3****, 9005.161 Da;* ***S4****, 9023.172 Da. Observed masses: 9054.703 Da, 8978.702 Da, 9004.724 Da, 9022.727 Da, 9038.723 Da, 9070.696 Da.*

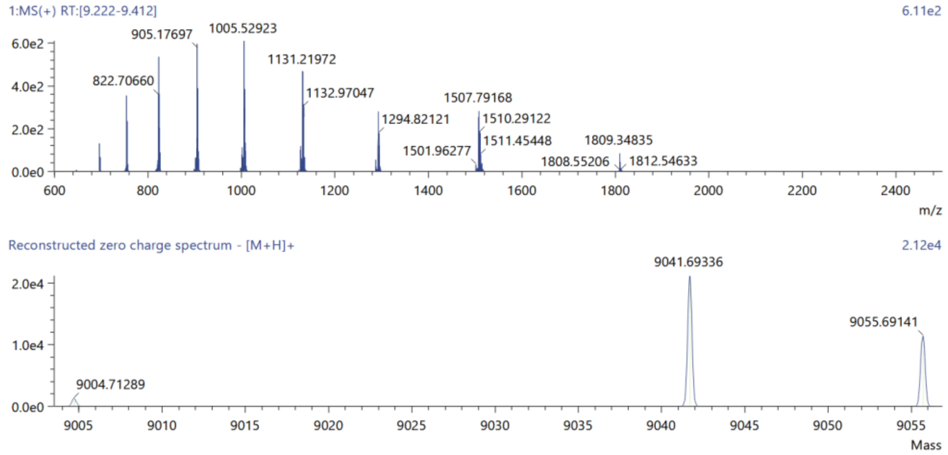

Figure S35. LC−MS analysis of the reaction mixture with Ubiquitin K63C: ion series and deconvoluted spectrum of protein peak 5; calculated mass: ***S3****, 9005.161 Da. Observed masses: 9041.693 Da, 9056.691 Da, 9004.713 Da, 9055.691 Da.*

Control experiment with Ubiquitin

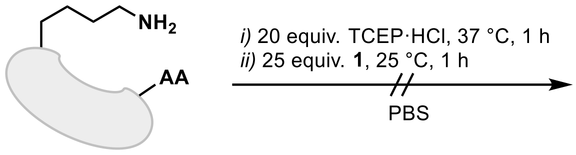

Ubiquitin

At 20–25 °C, 69 µL of Ubiquitin (0.25 mg/mL, 1.0 equiv.) in NaP_i buffer (pH 7.4, c = 50 mM) was added to a 1.5 mL Eppendorf tube. Next, 29 µL PBS was added. Then, 1.0 µL of a TCEP stock solution (40 mM, 20 equiv.) in H_2O was added. The mixture was vortexed for 5 seconds, transferred into a Thermocycler pre-heated at 37 °C, and incubated at 37 °C for 1 hour at 600 rpm. Next, 1.0 µL of a freshly prepared stock solution of **1** in DMSO (50 mM, 25 equiv.) was added to the mixture. The reaction mixture was vortexed for 5 seconds, transferred into a Thermocycler pre-heated at 25 °C, and incubated at 25 °C at 600 rpm for 1 hour. A stock solution of β-mercaptoethanol (1.0 µL, 50 mM, 25 equiv.) in H_2O was added, the mixture was incubated for 15 minutes at 25 °C, centrifuged for 3 minutes at 17,000 xg, and the supernatant was analyzed using LC–MS.

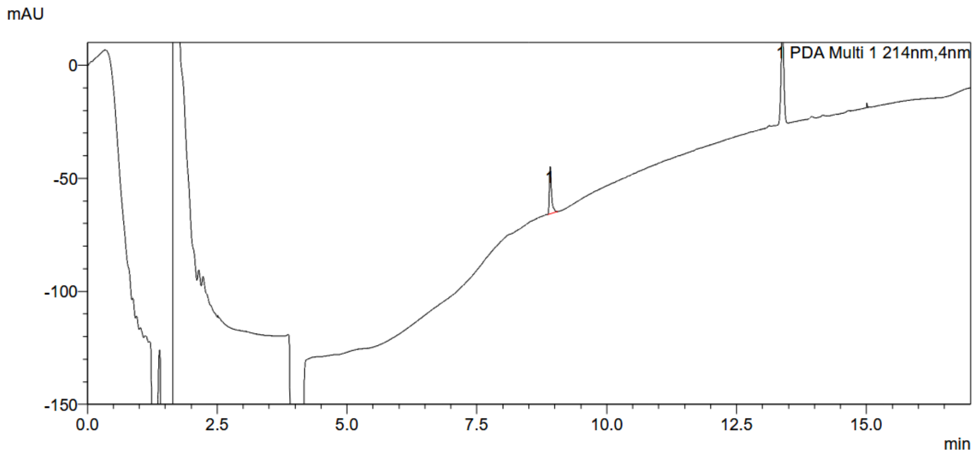

Figure S36. LC−MS analysis of the reaction mixture with Ubiquitin: UV trace.

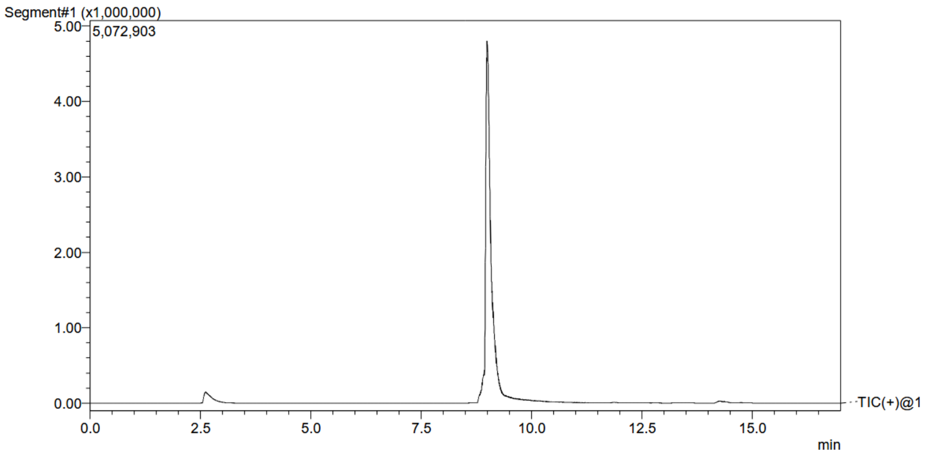

Figure S37. LC−MS analysis of the reaction mixture with Ubiquitin: total ion chromatogram.

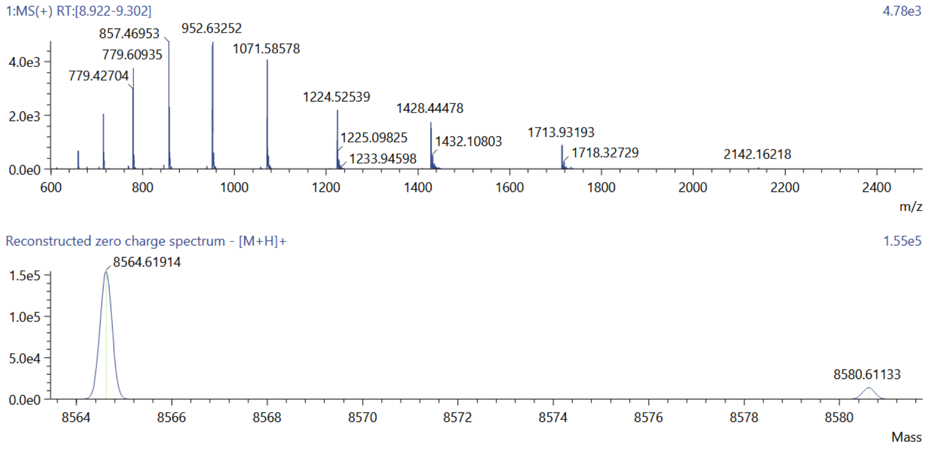

Figure S38. LC−MS analysis of the reaction mixture with Ubiquitin: ion series and deconvoluted spectrum of the protein peak; calculated mass: Ubiquitin, 8564.751 Da. Observed masses: 8564.619 Da, 8580.611 Da.

Experiments with HEK cells

The experiment was performed with three biological replicates.

In each case, confluent (~95%) HEK cells in 1x Gibco DMEM + GlutaMAX Medium supplemented with 10% of heat-inactivated FCS in a 10 cm culture plate were washed 2 times with 5 mL of DPBS and 10 mL of DPBS was added. Next, 100 µL of a freshly prepared 0.1 M stock solution of **1** in DMSO was added within 5 s (c_{final} = 1.0 mM) and the cells were incubated for 1 hour at 37 °C in 5% CO_2 in a humidified atmosphere. Next, each mixture was transferred to a 15 mL Falcon tube and centrifuged for 5 minutes at 500 xg at 4 °C. The supernatants were discarded, 2 mL of DPBS was added to each tube, and the cell pellets were resuspended by gentle shaking of the tube. Then, each mixture was centrifuged for 5 minutes at 500 xg at 4 °C. The cells were washed two more times with 2 mL DPBS, the last centrifugation step was performed for 5 minutes at 1,500 xg at 4 °C, the supernatants were discarded, and the cell pellets were frozen and kept at –78 °C until further processing.

To lyse the cells, the cell pellets were thawed, and each cell pellet was resuspended in 1.0 mL of the lysis buffer (HEPES (pH 7.4, 20 mM); 0.14 M NaCl; 0.5% (v/v) NP-40; 1 × cOmplete™, Mini, EDTA-free Protease Inhibitor Cocktail) by pipetting. The mixtures were incubated for 30 minutes at 4 °C after addition of the lysis buffer. The mixtures were vortexed for 10 seconds immediately, 10 minutes, and 20 minutes after addition of the lysis buffer. The tubes were centrifuged for 15 minutes at 21,000 xg at 4 °C, the supernatants were collected, and the protein concentration was determined via BCA assay.

Sample preparation for LC–MS/MS measurements

CuAAC reaction with biotin-azide

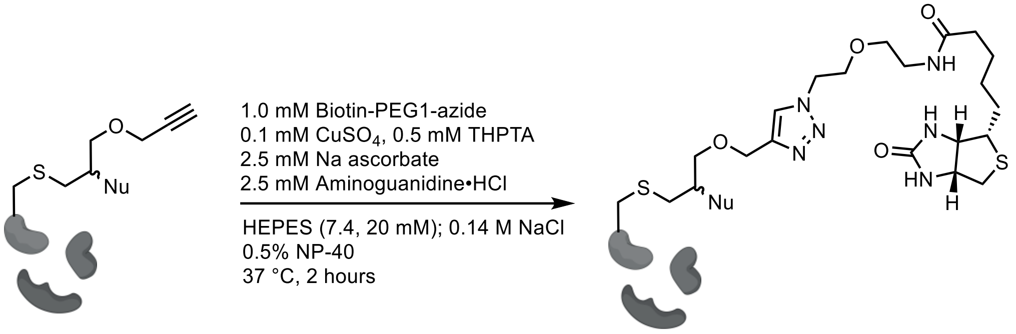

Lysis buffer (HEPES (pH 7.4, 20 mM); 0.14 M NaCl; 0.5% (v/v) NP-40; 1 × cOmplete™, Mini, EDTA-free Protease Inhibitor Cocktail) was added to 1.5 mL tubes containing lysates to obtain mixtures with 0.5 mg protein in 234 µL lysis buffer. Then, 13 µL of a 40 mM biotin-PEG1-azide stock solution in DMSO was added (c_{final} = 1.0 mM) and each mixture was vortexed for 1 s. Next, 10 µL of Cu-THPTA mixture (5 mM $CuSO_4$ and 25 mM THPTA in H_2O) premixed at 37 °C for 10 minutes was added ($c_{final}(CuSO_4)$ = 0.1 mM; c_{final}(THPTA) = 0.5 mM). Then, 5.0 µL of an aminoguanidine hydrochloride stock solution (0.25 M in H_2O) was added (c_{final} = 2.5 mM), followed by addition of 5.0 µL of a sodium ascorbate stock solution (0.25 M in H_2O; c_{final} = 2.5 mM). The mixtures were vortexed for 2 s, transferred into a Thermocycler pre-heated at 37 °C, and incubated at 37 °C for 2 hours at 1000 rpm.

The obtained samples were used for biotin enrichment.

Protocol for biotin enrichment

The same procedure was used for the enrichment of each replicate:

1. **Reduction and alkylation**: 65 µL of HEPES (pH 8.0, 1.0 M) was added to a lysate after CuAAC reaction (each mixture contained 0.5 mg protein in 500 µL). Then, 33 µL of a 20% (w/w) stock solution of SDS in H_2O and a 9.3 µL of a 0.70 M stock solution of TCEP in H_2O were added to the sample. The mixture was incubated for 30 minutes at 37 °C at 1000 rpm, 43 µL of a 0.60 M chloroacetamide stock solution in H_2O was added, and the mixture was incubated for 1 hour at 37 °C at 1000 rpm.
2. **SP3-digestion**: 100 µL of the magnetic beads stock (combined 1:1 Sera-Mag SpeedBeads (GE Healthcare, cat. no. 45152105050250) and Sera-Mag SpeedBeads (GE Healthcare, cat. no. 65152105050250)) was added to the reduced and alkylated lysate, the beads were resuspended by vortexing the mixture for 1 s, followed by addition of 750 µL of abs. EtOH. The mixture was incubated for 10 minutes at 24 °C at 1000 rpm, transferred to a magnetic rack, and the beads were washed three times with 1000 µL of 80 % (v/v) EtOH in H_2O according to the SP3-protocol[6]. The on-bead digestion was performed in 400 µL of HEPES buffer (pH 8.0, 50 mM) containing 10 µg of Trypsin (Promega, #V5111) at 37 °C at 1000 rpm for 18 hours.
3. **Heat treatment**: The sample was centrifuged for 10 minutes at 21,000 xg, the supernatant was transferred to a new tube and incubated for 10 minutes at 95 °C at 1000 rpm to ensure the inactivation of trypsin prior to addition to NeutrAvidin beads.

4. **Biotin-NeutrAvidin binding**: 125 µL of NeutrAvidin beads slurry (ThermoFischer, #29200) was washed 3 times with 1 mL of PBS. Each washing step included adding the buffer to the beads, vortexing the mixtures for 1 s, centrifuging at 1,700 xg for 2 minutes at 20–25 °C, and discarding the supernatant. Then, the heat-treated peptide mixture was added to the tube with washed NeutrAvidin beads at 20–25 °C, the mixture was vortexed for 2 s, and transferred to a Thermocycler pre-cooled to 25 °C. The mixture was incubated for 2 hours at 25 °C at 1000 rpm.
5. **Washing step**: The mixture was centrifuged at 1,700 xg for 2 minutes at 20–25 °C, the supernatant was discarded. The beads were washed five times with 1 mL of PBS and four times with 1 mL of H_2O. Each washing step included adding PBS or water to the beads, vortexing the mixture for 3 s, centrifuging at 1,700 xg for 2 minutes at 20–25 °C, and discarding the supernatant.
6. **Peptide release**: The washed beads were resuspended in 150 µL of the elution buffer (80% MeCN, 0.1% formic acid (v/v) in H_2O), vortexed for 3 s, and incubated for 10 minutes at 25 °C at 1000 rpm[7]. The mixture was centrifuged at 1,700 xg for 2 minutes at 20–25 °C, the supernatant was transferred to a new tube. Then, the beads were resuspended in 150 µL of the elution buffer and incubated for 10 minutes at 72 °C at 1000 rpm. The mixture was centrifuged at 1,700 xg for 2 minutes at 20–25 °C, the supernatant was combined with the mixture from the first elution step.
7. **Washing step**: The beads were washed 2 times with 100 µL of the release buffer. Each washing step included adding the release buffer (80% MeCN, 0.1% formic acid (v/v) in H_2O) to the beads, vortexing the mixtures for 3 s, centrifuging at 1,700 xg for 2 minutes at 20–25 °C, and combining the supernatant with the mixture from step 6. The combined mixture in the elution buffer was evaporated using SpeedVac.
8. **Separation of the remaining beads**: The pellet obtained in step 7 was washed 3 times with H_2O. First, the pellet was resuspended in 35 µL of H_2O, vortexed for 15 s, and centrifuged for 5 min at 21,000 xg at 20–25 °C. Then, 30 µL of the supernatant was carefully transferred to a new tube. The washing step was repeated two more times with the addition and transferring 30 µL of H_2O each time. The combined mixtures without remaining beads were evaporated using SpeedVac and analyzed via LC–MS/MS.

SDS-PAGE analysis

CuAAC reaction with 6-FAM-azide

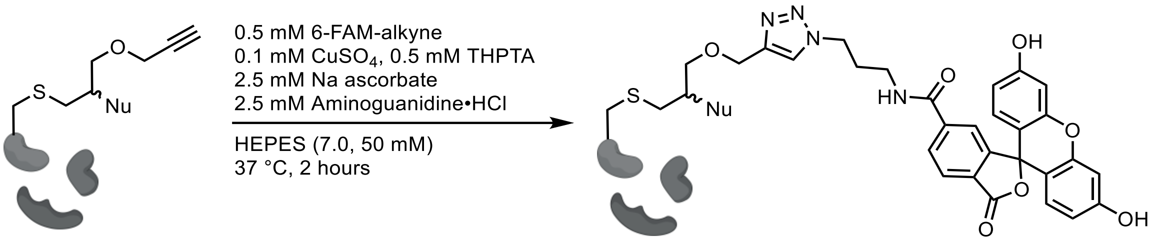

In each case, the reactions were performed as follows: At 20–25 °C, HEPES (pH 7.0; 50 mM) buffer was added to the lysate to obtain final volume of 94 µL. Then, 2.1 µL of a 24 mM 6-FAM azide stock solution in DMSO was added (c_{final} = 0.5 mM). Next, 2.0 µL of a Cu-THPTA mixture (5 mM $CuSO_4$ and 25 mM THPTA in H_2O) premixed at 37 °C for 10 minutes was added ($c_{final}(CuSO_4)$ = 0.1 mM; c_{final}(THPTA) = 0.5 mM). Next, 1.0 µL of a sodium ascorbate stock solution (0.25 M in H_2O) was added (c_{final} = 2.5 mM), followed by addition of 1.0 µL of an aminoguanidinium hydrochloride stock solution (0.25 M in H_2O, c_{final} = 2.5 mM). The mixture was vortexed for 2 s, transferred into a Thermocycler pre-heated at 37 °C, and incubated at 37 °C for 2 hours at 600 rpm.

Next, the samples were rebuffered five times in HEPES (pH 7.0, 50 mM) utilizing 3 kDa Amicon filter units. After rebuffering, the sample volumes were adjusted to 45 µL by addition of the HEPES buffer and the reaction mixtures were analyzed via SDS-PAGE analysis loading 10 µg protein per lane.

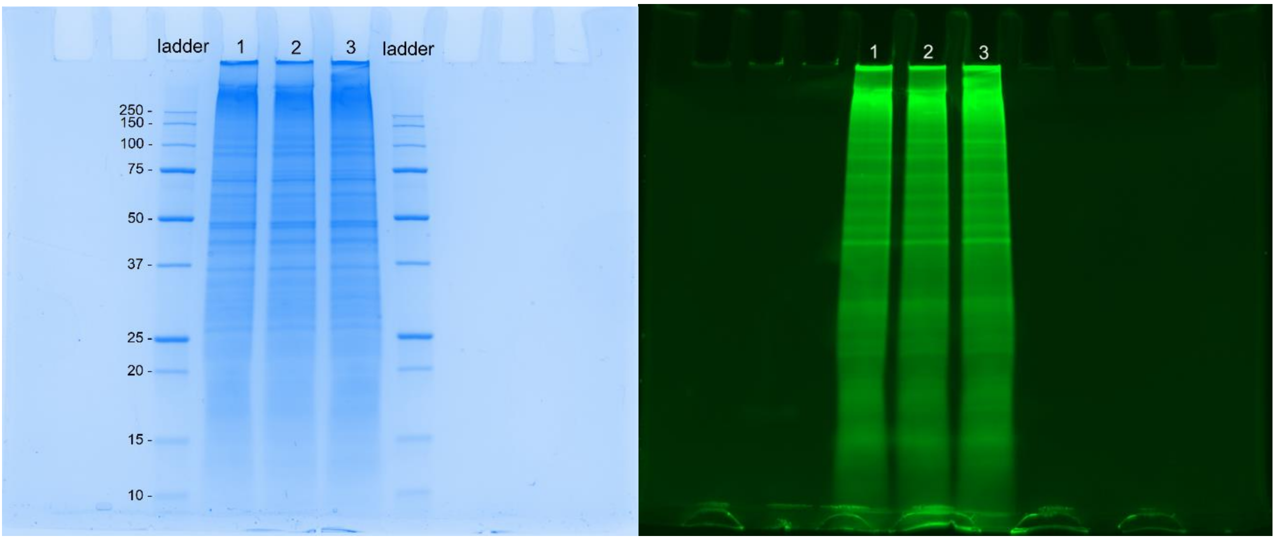

Figure S39. Protein gel after Coomassie blue staining (left) and the fluorescence image of the same gel (right).

DATA ANALYSIS

Labeling efficiency and enrichment specificity

Processing of raw data was performed using MaxQuant 2.6.3.0[8] on the HPC system Raven at the Max Planck Computing and Data Facility. MS^2 spectra were assigned to the reference proteome from Uniprot (*H. sapiens*: UP000005640). Trypsin specificity was required and a maximum of two missed cleavages was allowed. Carbamidomethylation of cysteine, oxidation of methionine, and protein N-terminal acetylation were set as variable modifications.

The **TTD**-based modification was defined as a variable modification on one of the nucleophilic amino acids (Cys, Lys, His, Ser, Thr, Glu, Asp, Arg, Tyr) with formula $C_{20}H_{32}N_6O_5S$ and H_2O as a neutral loss:

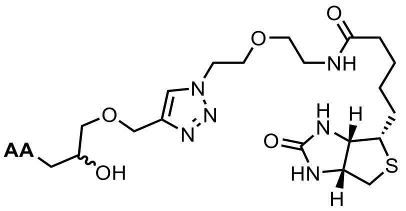

A false discovery rate of 1% for peptide spectrum matches and proteins was applied. The total number of peptides of interest was obtained after filtering off the decoy peptides and potential contaminants from the output table:

replicate	# All peptides	# modified peptides	% unmodified peptides
1	5903	5446	7.7%
2	5755	5353	7.0%
3	5967	5512	7.6%

For the analysis of the modification selectivity, only modified peptides with localization probability of at least 95% were considered:

AA	# modified peptides, replicate 1	# modified peptides, replicate 2	# modified peptides, replicate 3
Cys	1324	1290	1297
Lys	79	84	62
Arg	43	35	34
Glu	34	20	20
His	24	25	23
Ser	14	21	24
Asp	14	19	16
Thr	11	8	11
Tyr	6	4	7

Cross-link data analysis

Cross-link search

Raw data were processed using MSConvert 3.0.24[9] to convert the raw files to mzML format for the subsequent mass recalibration[10-12]. An open modification search with MSFragger 4.1[13] was then performed to recalibrate precursor masses, and the obtained files were converted to mgf format with MSConvert 3.0.24[9]. The mgf spectra were split for parallel database search on the HPC system Raven at the Max Planck Computing and Data Facility.

Each of the peakfiles was searched separately against the *H. sapiens* proteome (UP000005640) using xiSEARCH 1.8.7[14] with the following settings: MS^1 tolerance: 3 ppm; MS^2 tolerance: 5 ppm[12], allowing up to two missing monoisotopic peaks and three missed tryptic cleavages. Cysteine carbamidomethylation and oxidation of methionine were defined as variable modifications. –H_2O/–NH_3 were defined as losses.

The **TTD** cross-linker was defined as follows: the linkage mass was set to 450.204926 Da and the specificity was set to C and one of the following residues: C, H, K, E, D, S, T, R, Y, C-terminus, N-terminus. The cross-link search for each of the considered cross-link combinations was performed separately. Variable modifications to account for the hydrolysis of episulfonium with water (18.0105647 Da), or formation of a cross-link within a peptide (450.204926 Da) were also defined. Non-covalent cross-link with a mass of zero was included in all searches to flag spectra potentially arising from gas-phase associated peptides[15].

The output files obtained for all the cross-link combinations and biological replicates were combined together. The matches obtained for the non-covalent cross-link were removed prior to false-discovery-rate (FDR) estimation, which was performed using xiFDR 2.3.5[14]. FDR estimation was performed separately for the intramolecular and intermolecular cross-links. In each case, the input file was filtered to obtain peptide pairs with at least three matched fragment ions in each cross-linked peptide. For the intramolecular cross-links, the ambiguity on the residue pair and protein group levels was set to 1 and the results were filtered on residue pair level (1%) with the boosting feature enabled.

For the intermolecular cross-links, the ambiguity on the residue pair level was set to 1 and the results were filtered on residue pair level (1%) and protein pair level (1%) with the boosting feature enabled on the residue pair level. We verified that none of the detected cross-linked peptides belongs to a contaminant protein[16].

The obtained files were uploaded to xiView.org[17] and the list of the non-ambiguous cross-links was used for the further analysis.

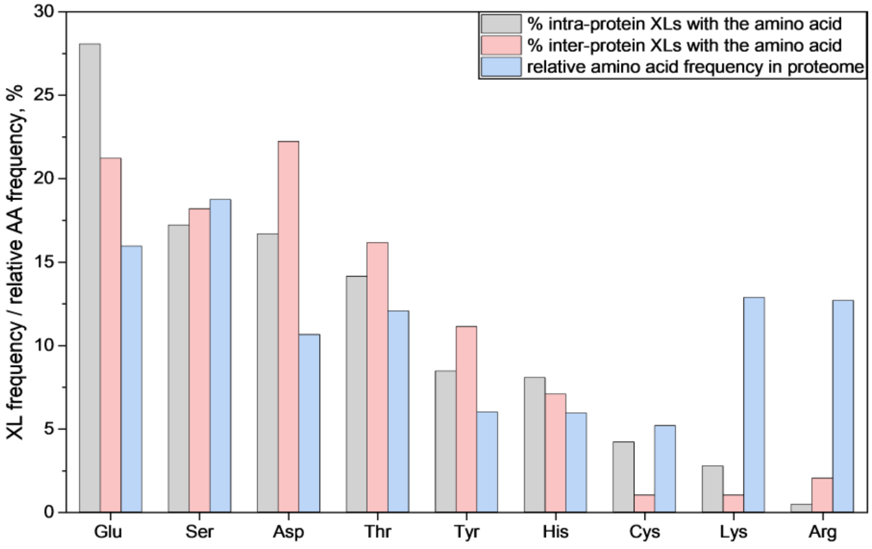

Figure S40. Relative frequencies of amino acids in the detected crosslinks and relative frequencies of the corresponding amino acids in the proteome.

Identified PPIs

Protein 1	Protein 2	# Unique Distance Restraints	STRING confidence score (functional interaction)	STRING confidence score (physical interaction)
P12956	P78527	6	0.999	0.999
Q15631	Q99598	3	0.999	0.999
P63244	Q8NC51	6	0.998	0.957
P17987	P49368	4	0.999	0.999
P48643	P78371	4	0.999	0.999
P49321	P84243	10	0.949	0.919
Q13619	Q86VP6	4	0.988	0.735
P62888	Q8NC51	6	0.987	0.915
Q8N5N7	Q9BYD3	6	0.989	0.96
Q15003	Q15021	3	0.999	0.999
P48643	Q99832	4	0.999	0.999
O43815	Q9Y3A3	6	0.999	0.98
Q13033	Q9Y3A3	6	0.999	0.98
P45880	Q9Y277	4	0.997	0.995
Q16555	Q9BPU6	2	0.99	0.965
P46821	Q15388	1	0.369	-
P63244	Q00341	7	0.774	0.508
Q6ZN66	Q99497	3	n.a.	n.a.
P63244	Q9UKY7	5	n.a.	n.a.
P07099	P10746	3	n.a.	n.a.
P40926	Q7RTU4	3	n.a.	n.a.
O43175	Q8N8E3	4	n.a.	n.a.

GO annotations

GO annotations for the proteins in the obtained datasets were exported from QuickGO[18] after restricting the search to Swiss-Prot entries in the canonical reference proteome. Only manually curated annotations backed by primary literature were considered for the analysis: direct or high-throughput experimental evidence (ECO:0000269 EXP, 0314 IDA, 0315 IMP, 0316 IGI, 0270 IEP, 0353 IPI, as well as the high-throughput counterparts 0007005 / 7001 / 7003 / 7007 / 6056), traceable author statements (ECO:0000304 TAS) and curator inferences (ECO:0000305 IC). Annotations based on sequence transfer or fully automatic pipelines (ECO:0000250, 0247, 0266, 0255, 0501) and entries lacking supporting data (ECO:0000303 NAS, 0000307 ND) were excluded, as well as all negation (`NOT|…`) qualifiers.

The filtered GO terms were then mapped to the goslim_generic subset in two distinct steps, depending on the size of each protein set. For proteins detected in the previously unreported PPIs (data presented in Figure 7 of the manuscript), we applied QuickGO's "Use these terms as a GO slim" option with relationships restricted to "is_a," "part_of" and "occurs_in" to preserve broader hierarchical context. For all proteins with at least two identified cross-links (intra- or intermolecular cross-links), QuickGO's "Use these terms as an exact match" option was used (data presented in Figure 4a of the manuscript).

Docking studies

General

Docking was carried out using the HADDOCK 2.4 web server[19] though the expert mode interface. PDB structures were used as input for the docking studies unless no available structure was available or the regions containing the cross-linked residues in the reported structures were unresolved. The distance restraints were used as unambiguous restraints between the cross-linked residues as follows: 23 Å for **TTD** and 35 Å for **DSSO**. “Center of mass” restraints were implemented in all calculations. The first model of the top-scoring cluster was used for the analysis, unless structural variability within the cluster indicated a lack of convergence. If the mean HADDOCK score (± SD) of the top-scoring cluster overlapped with the mean HADDOCK score (± SD) of another cluster, the first models of both clusters were analyzed.

CCT5 and CCT7

Input coordinates for both CCT5 (P48643) and CCT7 (Q99832) were extracted from the PDB structure of the Human TRiC complex (7NVL). The docking was performed in water using standard sampling parameters (1000 structures for rigid body docking; 200 structures for semi-flexible refinement; 200 structures for the final refinement and the analysis).

VDAC2 and VDAC3

Input coordinates for VDAC2 (P45880) were extracted from the PDB structure 9EII. AlphaFold-predicted structure (#AF-Q9Y277-F1-v4, pLDDT 95.4) was used for VDAC3 (Q9Y277). The docking was performed in DMSO using modified sampling parameters (10000 structures for rigid body docking; 500 structures for semi-flexible refinement; 500 structures for the final refinement and the analysis) and a C2 symmetry restraint was defined in all cases.

RACK1 and HDLBP

Input coordinates for RACK1 (P63244) were extracted from the PDB structure 4AOW. AlphaFold-predicted structure (#AF-Q00341-F1-v4, pLDDT 75.0) was used for HDLBP (Q00341). The docking was performed in water using modified sampling parameters (10000 structures for rigid body docking; 500 structures for semi-flexible refinement; 500 structures for the final refinement and the analysis).

Two top-scoring clusters (cluster 2 and cluster 3) with overlapping HADDOCK score values were obtained:

Calculated value	cluster 2	cluster 3
HADDOCK score	-117.3 ± 5.7	-108.5 ± 4.0
Cluster size	158	25
RMSD from the overall lowest-energy structure	14.3 ± 0.2	2.9 ± 0.6
Van der Waals energy	5.8 ± 4.5	-34.5 ± 5.0
Electrostatic energy	-736.0 ± 30.5	-419.6 ± 22.2
Desolvation energy	24.1 ± 3.8	9.9 ± 5.8
Restraints violation energy	0.0 ± 0.0	0.0 ± 0.0
Buried Surface Area	1459.2 ± 81.4	1898.4 ± 160.7
Z-Score	-1.7	-1.3

The top models in both clusters place RACK1 in a proximity to the KH-14 domain of Vigilin. The structure from cluster 3 was used for the analysis because of the negative E_{vdW}, lower RMSD and E_{desolv} values, and higher BSA value consistent with the formation of a stable complex:

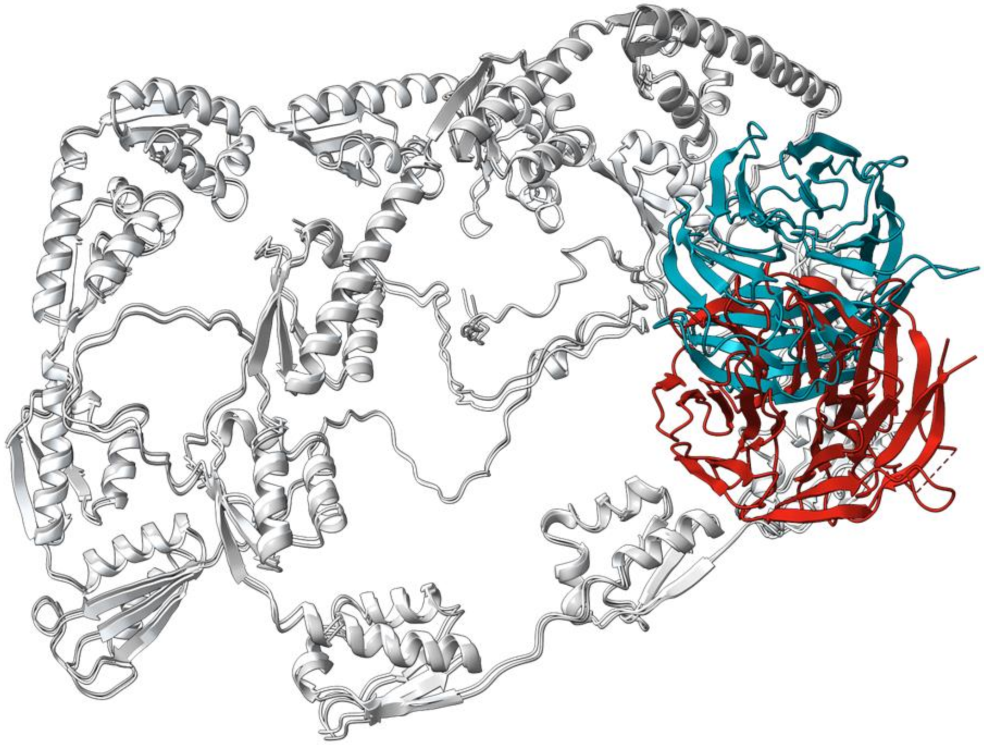

Figure S41. Aligned structures of best-scoring models for the two top-scoring clusters. Vigilin, cluster 2: white; Vigilin, cluster 3: grey; RACK1, cluster 2: red; RACK1, cluster 3: green.

CEP112 and PHGDH

Input coordinates for PHGDH (O43175) were extracted from the PDB structure 7DKM. AlphaFold-predicted structure (#AF-Q8N8E3-F1-v4, pLDDT 76.1) was used for CEP112 (Q8N8E3). The docking was performed in water using modified sampling parameters (10000 structures for rigid body docking; 500 structures for semi-flexible refinement; 500 structures for the final refinement and the analysis).

Estimation of the binding affinity in the complex was performed using the PRODIGY Web Server[20,21]. The obtained output includes the predicted value of the binding affinity (ΔG), the calculated value of the dissociation constant (K_d), numbers of intermolecular contacts (ICs) at the interface within the threshold distance of 5.5 Å, separately listed according to the contact properties, and percentage of the charged and apolar non-interacting surface (NIS%) of the complex:

**ΔG (kcal mol^{-1})**	**K_d (M) at 25 ℃**	**ICs charged-charged**	**ICs charged-polar**	**ICs charged-apolar**	**ICs polar-polar**	**ICs polar-apolar**	**ICs apolar-apolar**	**NIS charged**	**NIS apolar**
-8.8	3.4×10^{-7}	13	8	20	0	4	8	35.1	34.33

Data availability

The following data was deposited to Zenodo (record #15991853, zenodo.org/records/15991853):

- Raw NMR data
- Raw protein LC–MS data and MS peak files
- Raw SDS-PAGE data
- HADDOCK input and output files
- Python scripts used for data processing
- Quick GO annotation tables
- Xi Search output tables

The cross-linking mass spectrometry proteomics data have been deposited to the ProteomeXchange Consortium via the PRIDE[22,23] partner repository with the dataset identifier PXD066251 (10.6019/PXD066251): ebi.ac.uk/pride/archive/projects/PXD066251.

NMR DATA

NMR Characterization of 1

^{1}H NMR of **1**

600 MHz, CD_3CN, 298 K

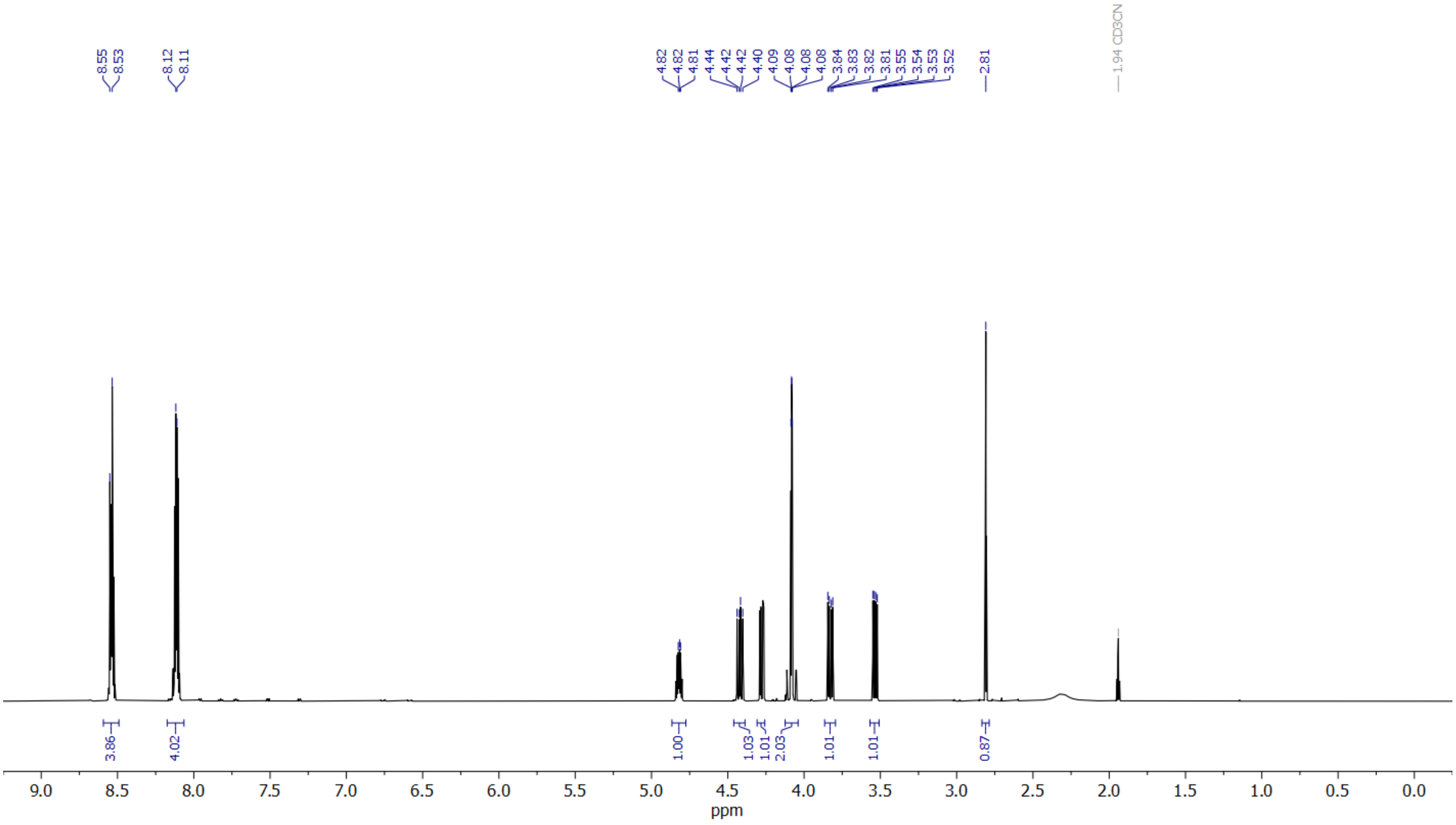

^{13}C NMR of **1**

151 MHz, CD_3CN, 298 K

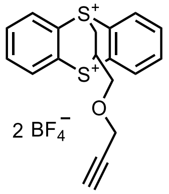

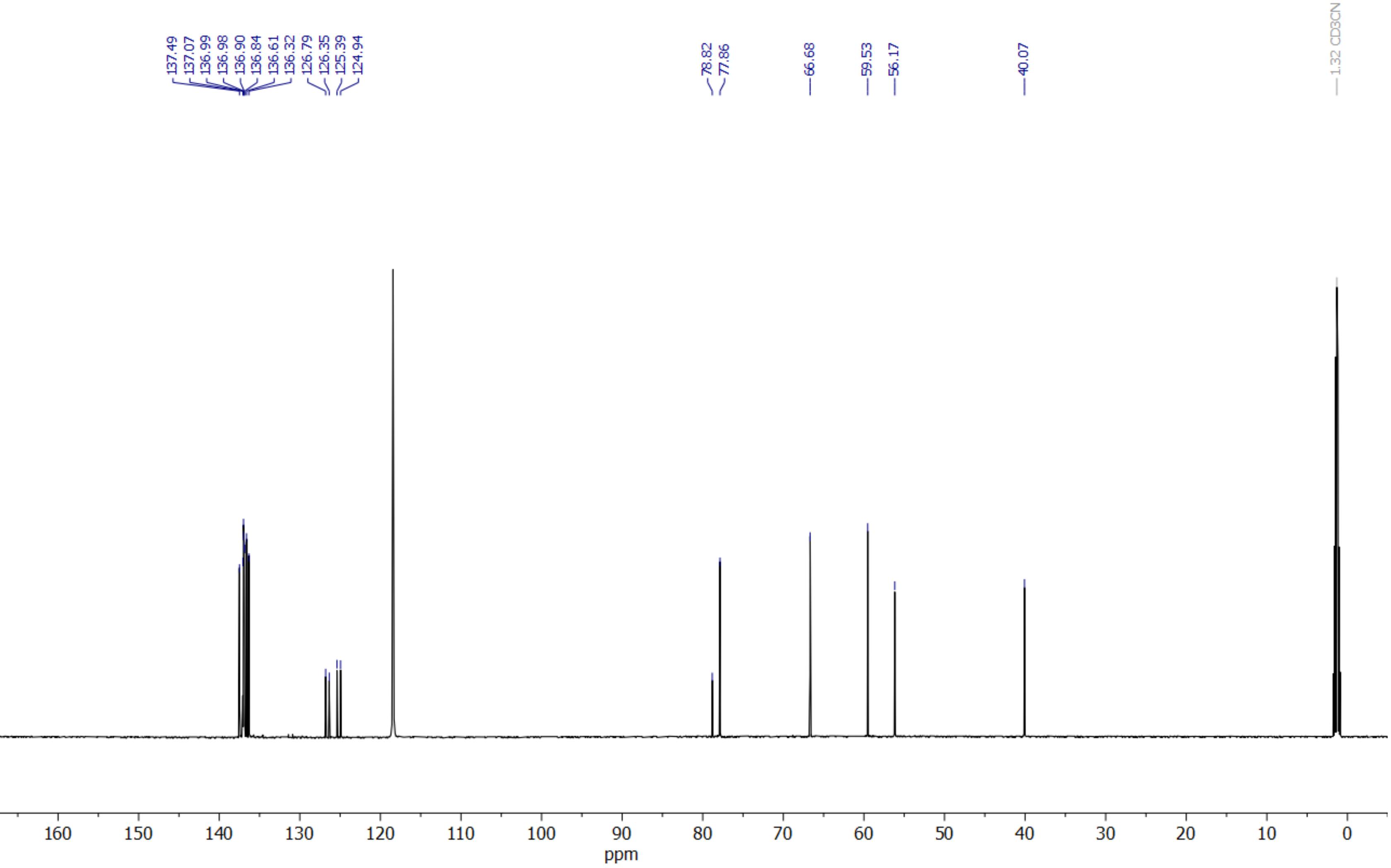

^{19}F NMR of **1**

471 MHz, CD_3CN, 298 K

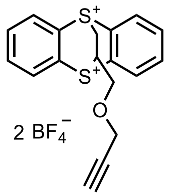

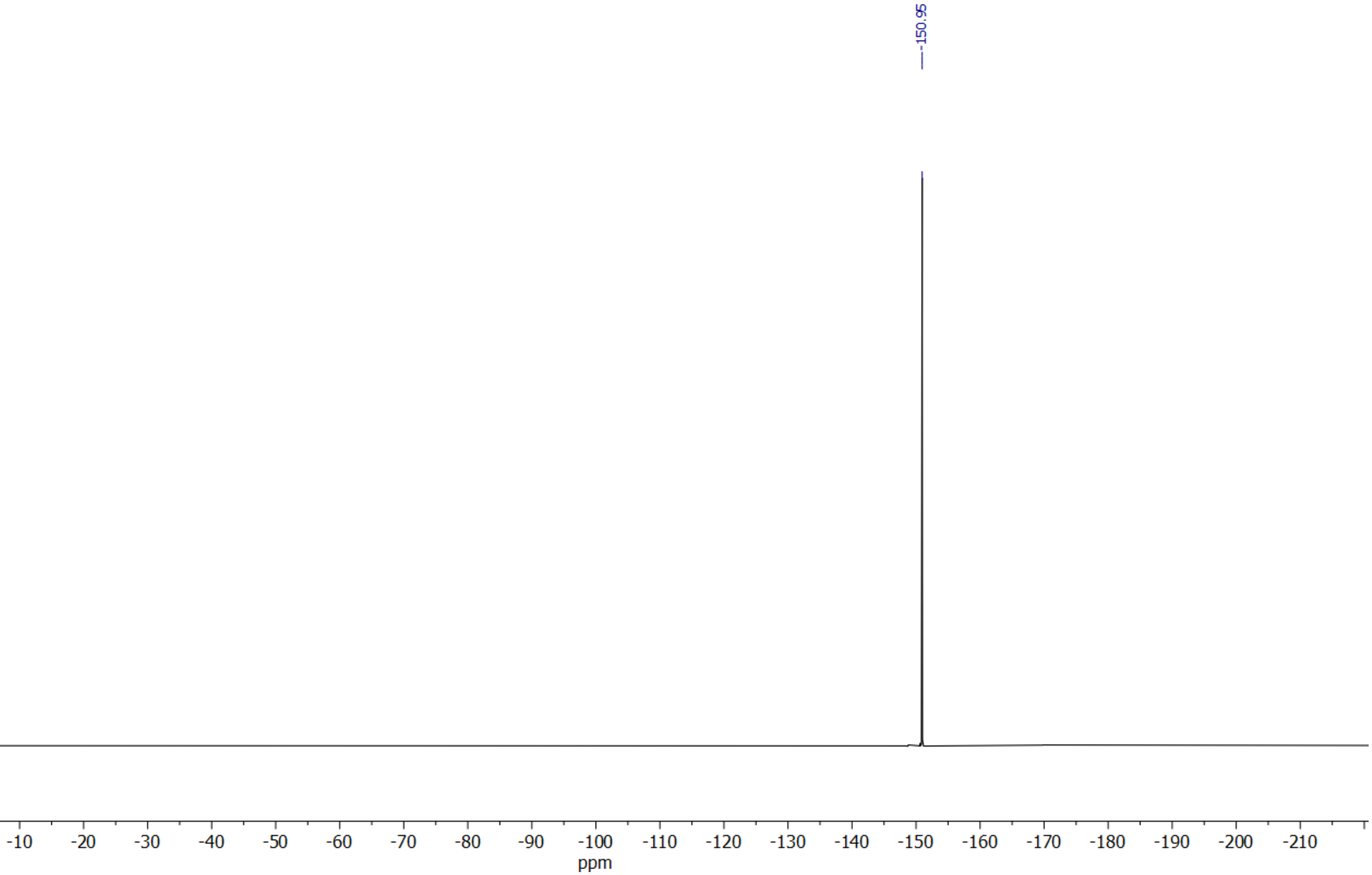

NMR Characterization of 2

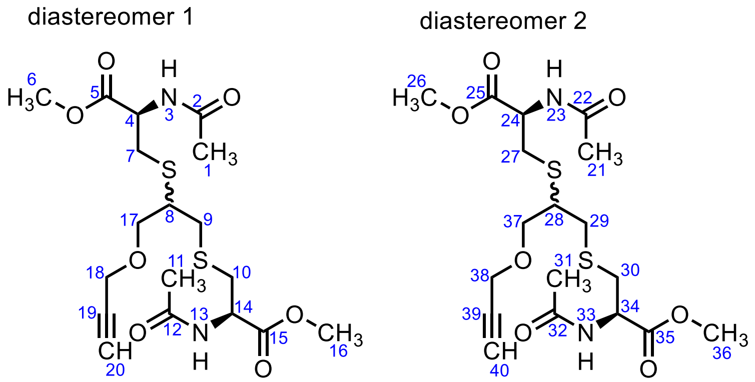

Table S1. Characterization table of 2 (diastereomer 1).

Atom	δ (ppm)	J	HSQC	HMBC	COSY	ROESY	15N-HMBC
C1	22.29		1	3			
H1	1.859	s (o.l.)	1	2		3	3
C2	169.45			1, 3, 4			
N3	-259.6						1, 3, 4, 7a, 7b
H3	8.376/8.374/8.371/8.365	d 7.9(4)		1, 2, 4, 5, 7	4	1, 4, 7b	3
C4	52.43/52.41		4	3, 7a, 7b			
H4	4.438	m (o.l.)	4	2, 5, 7	3, 7a, 7b	3	3
C5	171.19			3, 4, 6, 7a, 7b			
C6	52.08		6				
H6	3.639	s (o.l.)	6	5			
C7	32.21		7a, 7b	3, 4, 8			
H7a	2.954	d 13.7(7b), d 5.5(4)	7	4, 5, 8	4, 7b		3
H7b	2.826	d 13.7(7a), d 8.6(4)	7	4, 5, 8	4, 7a	3	3
C8	45.19		8	7a, 7b, 9, 17a, 17b			
H8	3.046	m (o.l.)	8	7, 9, 17	9, 17a, 17b	17a, 17b	
C9	34.33		9	8, 10a, 10b, 17a, 17b			
H9	2.773	m (o.l.)	9	8, 10, 17	8	17a, 17b	
C10	33.56		10a, 10b	9, 13, 14			
H10a	2.921	d 13.7(10b), d 5.3(14)	10	9, 14, 15	10b, 14		13
H10b	2.778	d 13.7(10a), d 9.6(14)	10	9, 14, 15	10a, 14	13	13

**C11**	22.29		11	13			
**H11**	1.859	s (o.l.)	11	12		13	13
**C12**	169.45			11, 13, 14			
**N13**	-259.6						10a, 10b, 11, 13, 14
**H13**	8.376/8.374/8.371/8.365	d 7.9(14)		10, 11, 12, 14, 15	14	10b, 11, 14	13
**C14**	52.08		14	10a, 10b, 13			
**H14**	4.438	m (o.l.)	14	10, 12, 15	10a, 10b, 13	13	13
**C15**	171.25/171.24			10a, 10b, 13, 14, 16			
**C16**	52.08		16				
**H16**	3.639	s (o.l.)	16	15			
**C17**	70.62		17a, 17b	8, 9, 18			
**H17a**	3.648	d 9.8(17b), d 5.1(8)	17	8, 9, 18	8, 17b	8, 9, 18	
**H17b**	3.574	d 9.8(17a), d 5.1(8)	17	8, 9, 18	8, 17a	8, 9, 18	
**C18**	57.71/57.69		18	17a, 17b, 20			
**H18**	4.155/4.151	d 2.5(20)	18	17, 19, 20	20	17a, 17b	
**C19**	80.03/80.01			18, 20			
**C20**	77.46/77.44		20	18			
**H20**	3.438/3.434	t 2.5(18)	20	18, 19	18		

Table S2. Characterization table of 2 (diastereomer 2).

Atom	δ (ppm)	J	HSQC	HMBC	COSY	ROESY	15N-HMBC
**C21**	22.29		21	23			
**H21**	1.859	s (o.l.)	21	22		23	23
**C22**	169.45			21, 23, 24			
**N23**	-259.6		23				21, 23, 24, 27a, 27b
**H23**	8.376/8.374/8.371/8.365	d 7.9(24)	23	21, 22, 24, 25, 27	24	21, 24, 27b	23
**C24**	52.41/52.43		24	23, 27a, 27b			
**H24**	4.438	m (o.l.)	24	22, 25, 27	23, 27a, 27b	23	23
**C25**	171.19			23, 24, 26, 27a, 27b			
**C26**	52.08		26				
**H26**	3.639	s (o.l.)	26	25			
**C27**	32.21		27a, 27b	23, 24, 28			
**H27a**	2.978	d 13.7(27b), d 5.5(24)	27	24, 25, 28	24, 27b		23
**H27b**	2.812	d 13.7(27a), d 8.6(24)	27	24, 25, 28	24, 27a	23	23

**C28**	45.26		28	27a, 27b, 29a, 29b, 37a, 37b			
**H28**	3.036	m (o.l.)	28	27, 29, 37	29a, 29b, 37a, 37b	37a, 37b	
**C29**	34.49		29a, 29b	28, 30a, 30b, 37a, 37b			
**H29a**	2.780	m (o.l.)	29	28, 30, 37	28		
**H29b**	2.757	m (o.l.)	29	28, 30, 37	28		
**C30**	33.68		30a, 30b	29a, 29b, 33, 34			
**H30a**	2.909	d 13.7(30b), d 5.5(34)	30	29, 34, 35	30b, 34		33
**H30b**	2.791	d 13.7(30a), d 8.3(34)	30	29, 34, 35	30a, 34	33	33
**C31**	22.29		31	33			
**H31**	1.859	s (o.l.)	31	32, 35		33	33
**C32**	169.45			31, 33, 34			
**N33**	-259.6						30a, 30b, 31, 33, 34
**H33**	8.376/8.374/8.371/8.365	d 7.9(34)		30, 31, 32, 34, 35	34	30b, 31, 34	33
**C34**	52.18		34	30a, 30b, 33			
**H34**	4.438	m (o.l.)	34	30, 32, 35	30a, 30b, 33	33	33
**C35**	171.24/171.25			30a, 30b, 31, 33, 34			
**C36**	52.08		36				
**H36**	3.639	s (o.l.)	36				
**C37**	70.69		37a, 37b	28, 29a, 29b, 38			
**H37a**	3.660	d 9.9(37b), d 5.1(28)	37	28, 29, 38	28, 37b	28, 38	
**H37b**	3.568	d 9.9(37a), d 5.9(28)	37	28, 29, 38	28, 37a	28, 38	
**C38**	57.69/57.71		38	37a, 37b, 40			
**H38**	4.151/4.155	d 2.5(40)	38	37, 39, 40	40	37a, 37b	
**C39**	80.01/80.03			38, 40			
**C40**	77.44/77.46		40	38			
**H40**	3.434/3.438	t 2.5(38)	40	38, 39	38		

^{1}H NMR of **2**

600 MHz, DMSO-d$_6$, 298 K

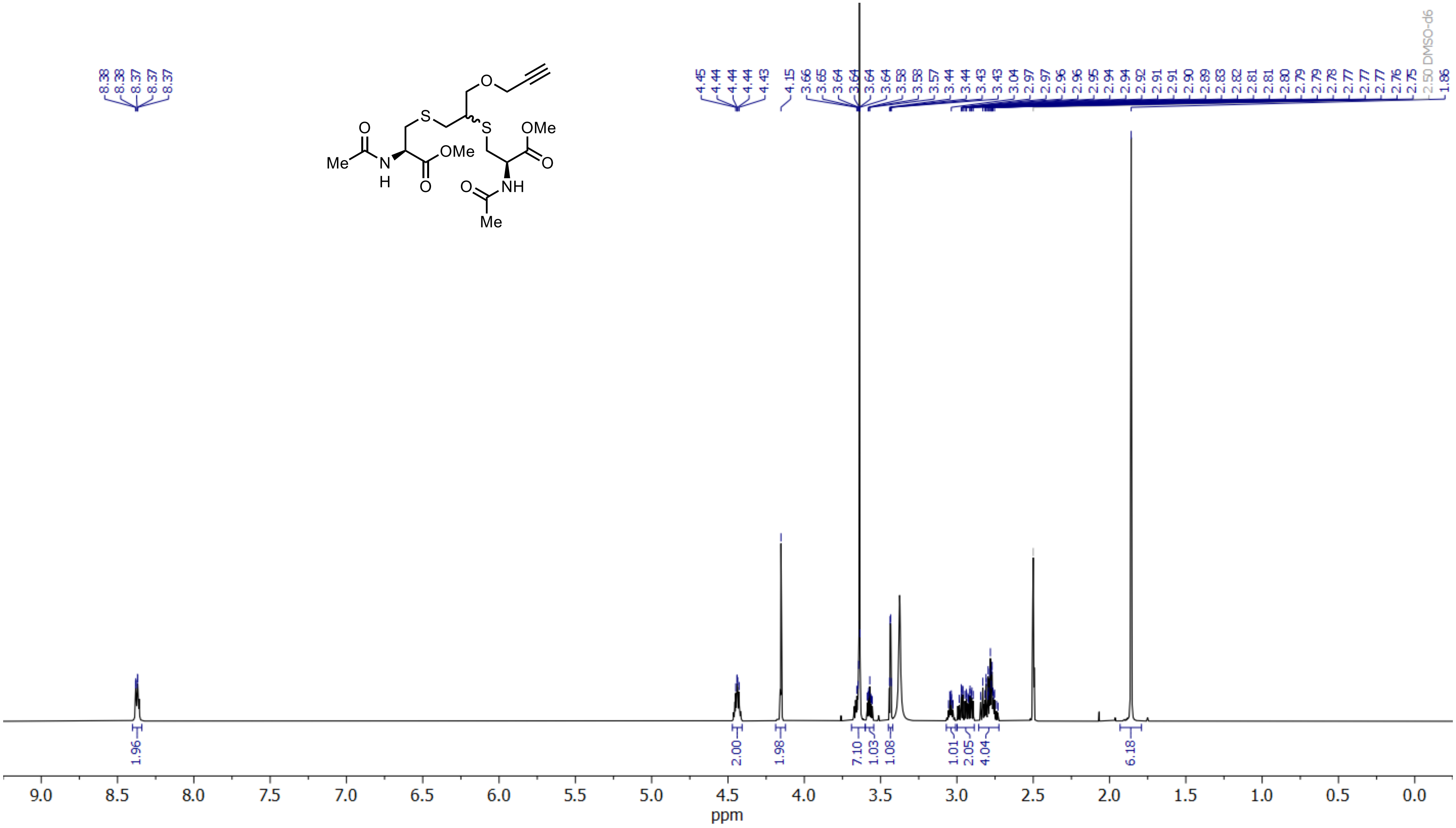

^{13}C NMR of **2**

151 MHz, DMSO-d$_6$, 298 K

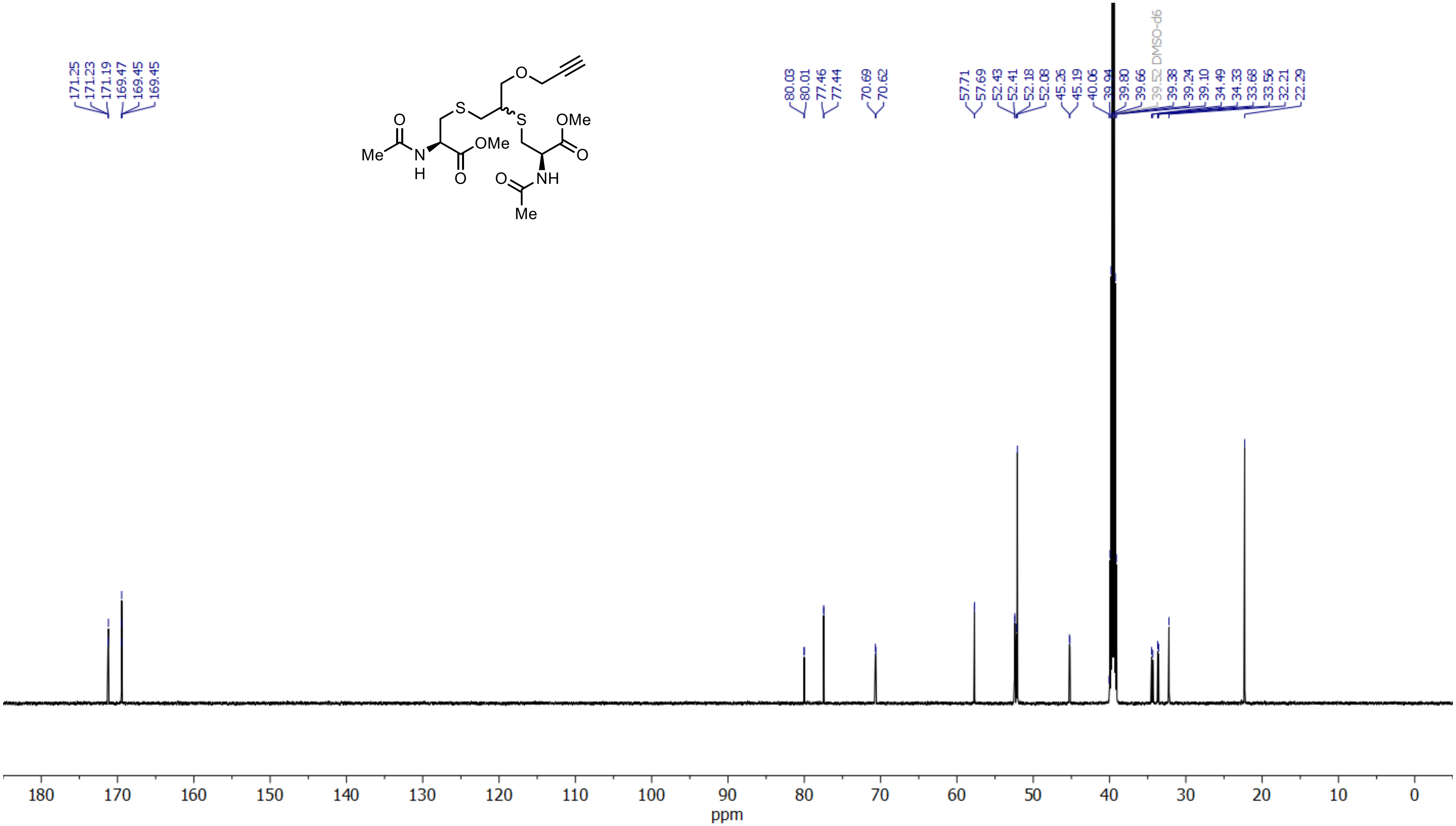

HSQC-NMR of **2**

DMSO-d6, 298 K

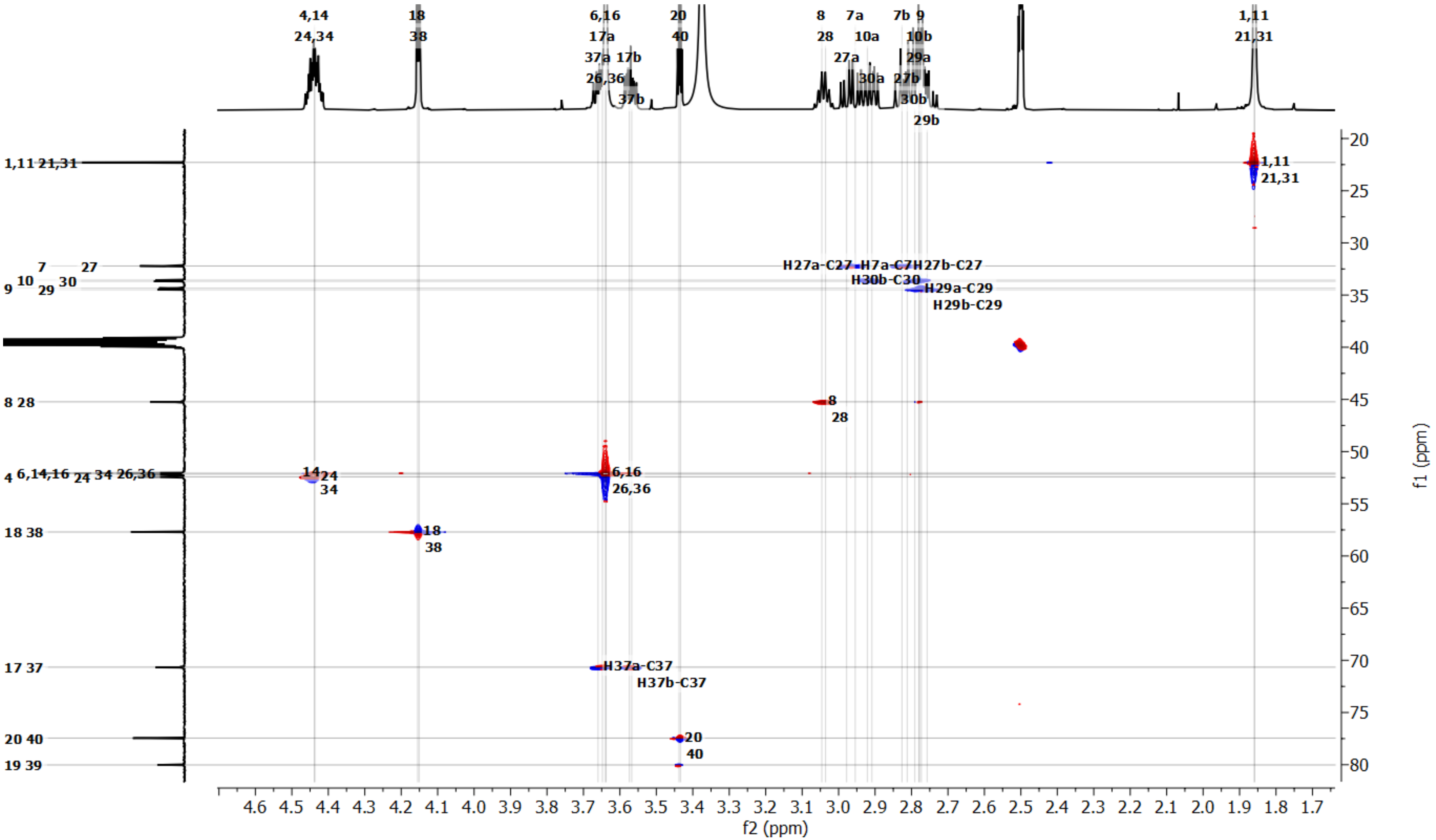

HMBC-NMR of **2**

DMSO-d6, 298 K

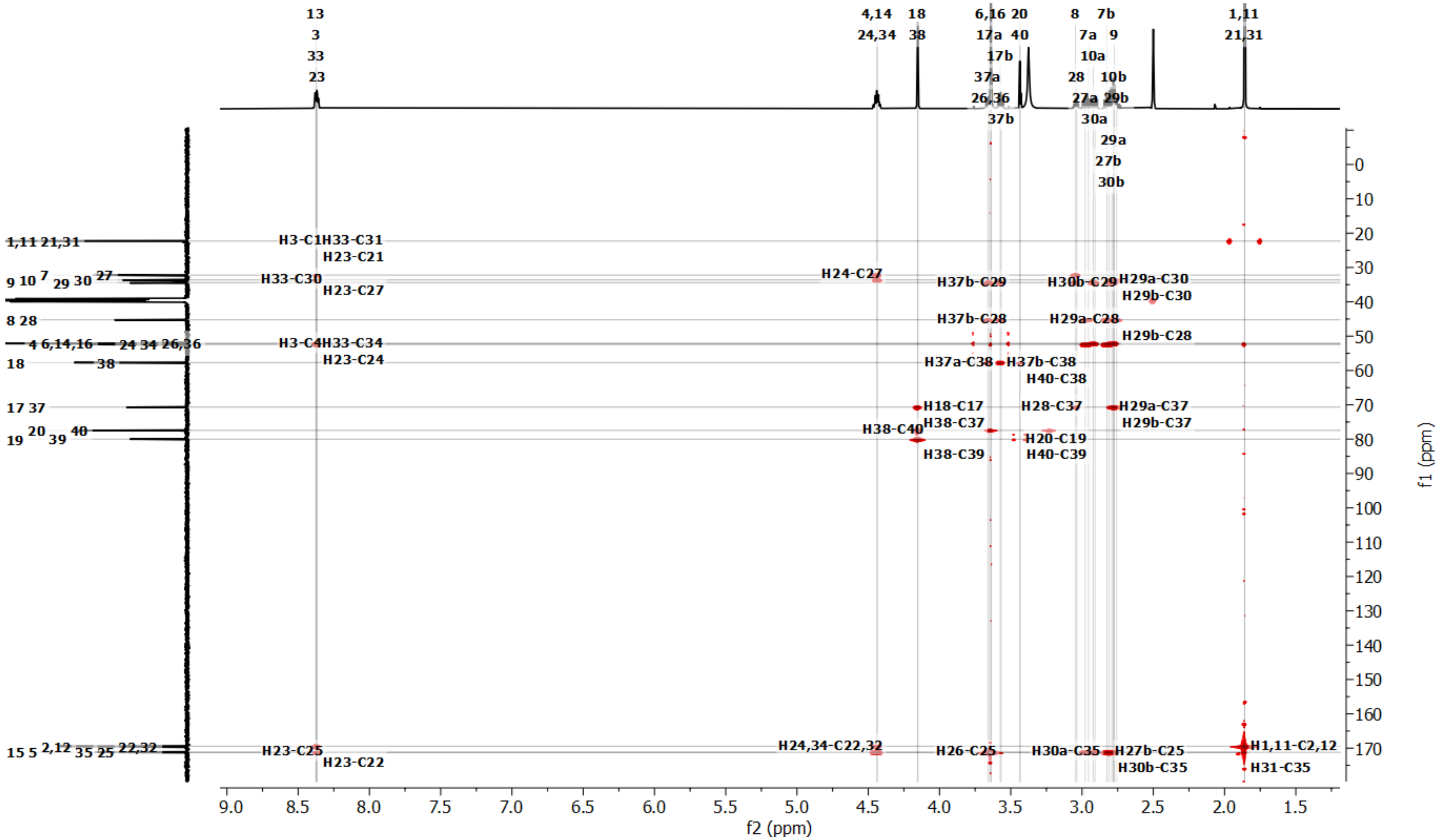

COSY-NMR of **2**

DMSO-d_6, 298 K

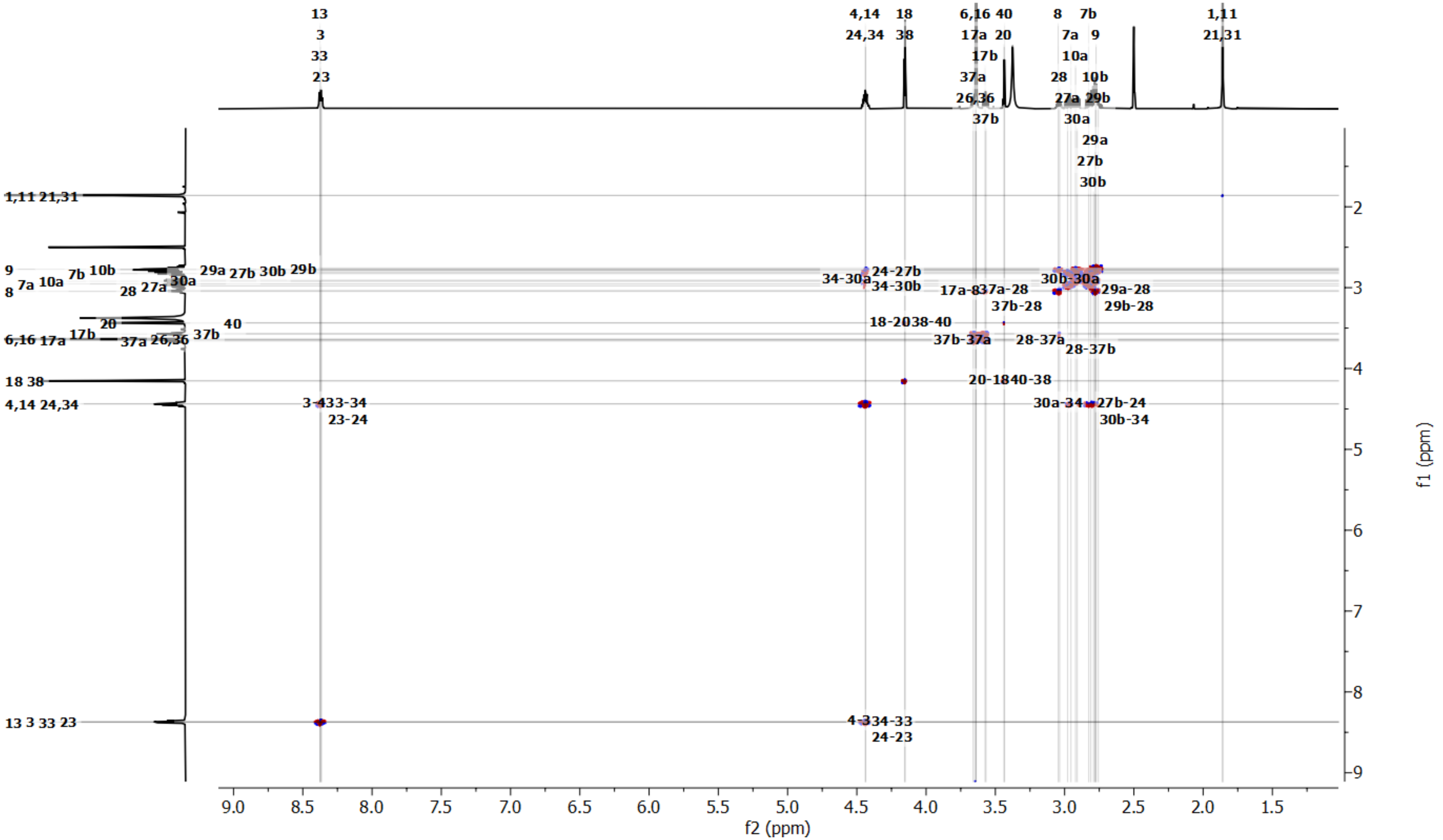

ROESY-NMR of **2**

DMSO-d6, 298 K

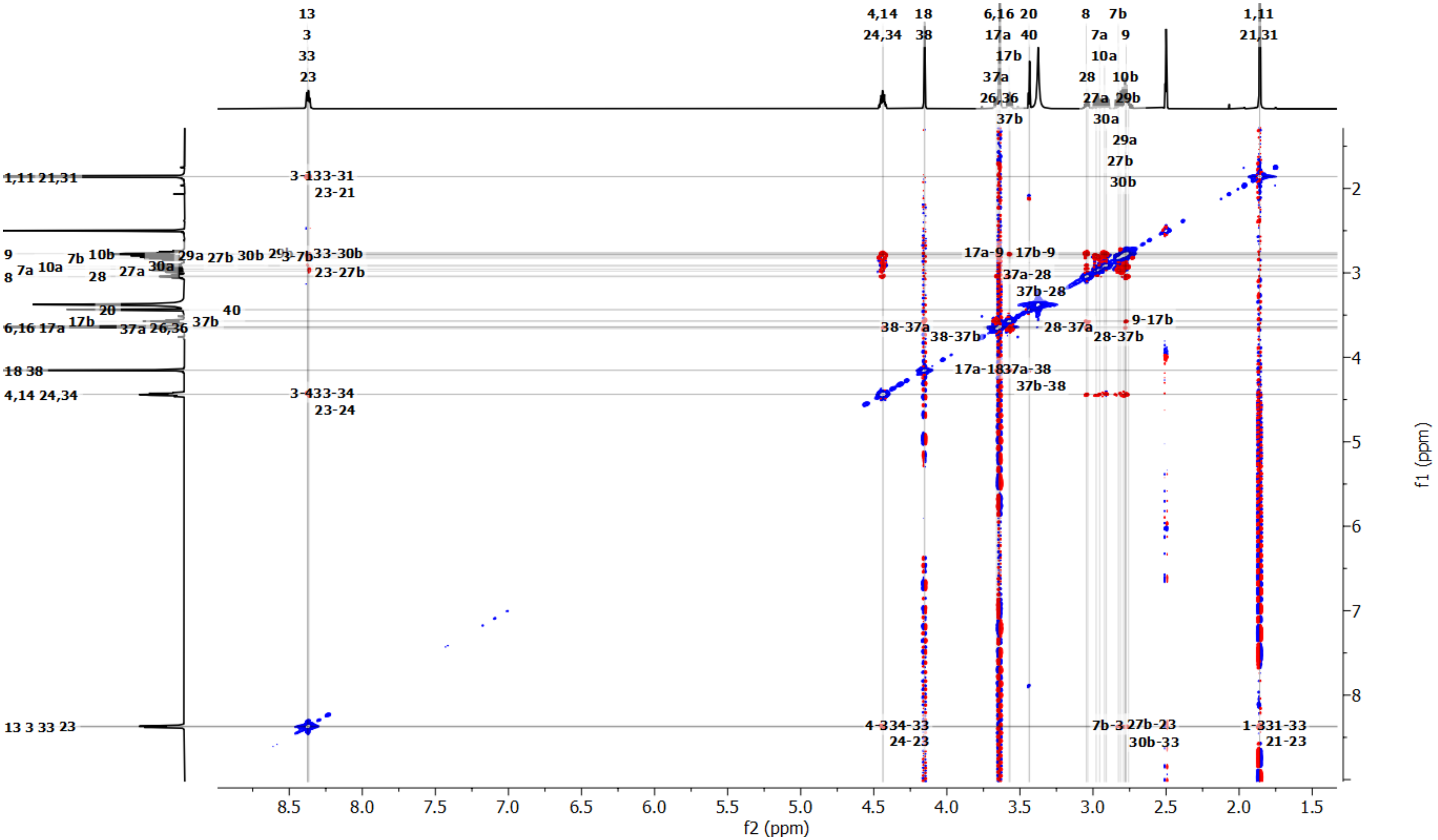

Alkenyl TT salts 1e, 1z, 1t

Deprotonation product 1e

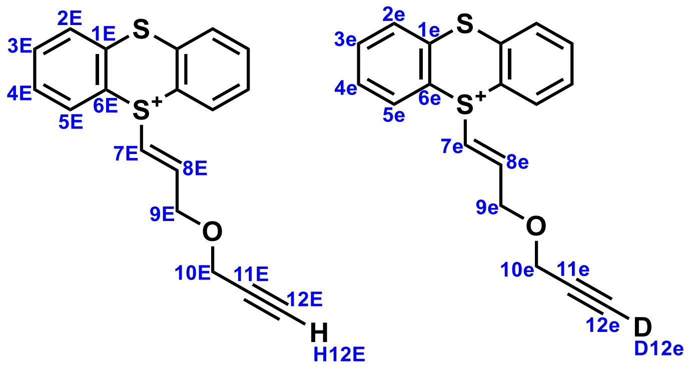

Table S3. Characterization table of 1e.

Atom	δ (ppm)	J	HSQC	HMQC	COSY	NOESY
**C1e**	135.95			3e, 5e		
**C2e**	130.29		2e	3e, 4e		
**H2e**	7.981	d 8.0(3e), d 1.3(4e), d 0.4(5e)	2e	4e, 6e	3e	
**C3e**	134.63		3e			
**H3e**	7.839	d 8.0(2e), d 7.5(4e), d 1.4(5e)	3e	1e, 2e, 4e, 5e	2e, 4e	
**C4e**	129.77		4e	2e, 3e		
**H4e**	7.730	d 8.0(5e), d 7.5(3e), d 1.3(2e)	4e	2e, 6e	3e, 5e	5e
**C5e**	133.19		5e	3e		
**H5e**	8.170	d 8.0(4e), d 0.4(2e), d 1.4(3e)	5e	1e	4e	4e, 7e
**C6e**	118.88			2e, 4e		
**C7e**	112.08		7e	8e, 9e		
**H7e**	6.680	d 14.8(8e), t 1.6(9e)	7e	9e	8e, 9e	5e
**C8e**	146.68		8e	9e		
**H8e**	6.629	d 14.8(7e), t 3.9(9e)	8e	7e, 9e	7e, 9e	9e
**C9e**	68.04		9e	7e, 8e, 10e		
**H9e**	4.274	d 3.9(8e), d 1.6(7e)	9e	7e, 8e	7e, 8e	8e, 10e
**C10e**	58.04		10e			
**H10e**	4.163	s	10e	9e, 11e, 12e		9e
**C11e**	78.45	(from HMQC)		10e		

**C12e**	76.20	t ~39 Hz(D12e; from HMQC)		10e		
**D12e**	n.d.					
**H10E**	4.165	d 2.4(12E)				
**C12E**	76.46					
**H12E**	2.812	t 2.4(10E)				

Deprotonation product 1z

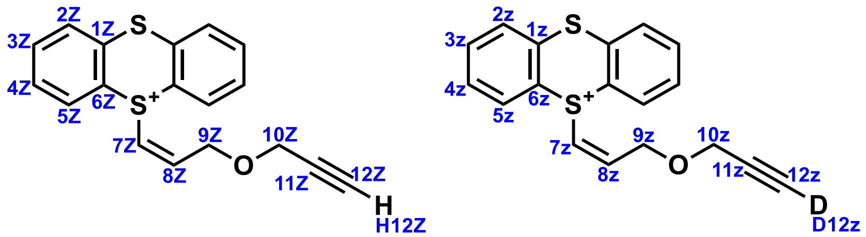

Table S4. Characterization table of 1z.

Atom	δ (ppm)	J	HSQC	HMQC	COSY	NOESY
**C1z**	135.99					
**C2z**	130.51		2z			
**H2z**	7.981	m (o.l.)	2z	4z, 6z	3z	
**C3z**	134.12		3z	5z		
**H3z**	7.795	d 8.0(2z), d 7.5(4z), d 1.4(5z)	3z	5z	2z, 4z	
**C4z**	129.55		4z	2z		
**H4z**	7.698	d 8.0(5z), d 7.5(3z), d 1.3(2z)	4z	6z	3z, 5z	5z
**C5z**	132.97		5z	3z		
**H5z**	8.170	m (o.l.)	5z	3z	4z	4z, 7z, 10z
**C6z**	121.91			2z, 4z		
**C7z**	115.36		7z	8z, 9z		
**H7z**	6.774	d 9.6(8z), t 1.3(9z)	7z	9z	8z, 9z	5z
**C8z**	146.37		8z	9z		
**H8z**	6.800	d 9.6(7z), t 3.5(9z)	8z	7z	7z, 9z	9z
**C9z**	66.95		9z	7z, 10z		
**H9z**	4.491	dm 3.5(8z)	9z	7z, 8z	7z, 8z	8z

**C10z**	58.34		10z			
**H10z**	4.461	s	10z	9z, 11z, 12z		5z
**C11z**	78.29	(from HMQC)		10z		
**C12z**	76.94	t ~38.0 Hz(D12z; from HMQC)		10z		
**D12z**	n.d.					
**H10Z**	4.462	d 2.4(12Z)				
**C12Z**	77.20					
**H12Z**	3.051	t 2.4(10Z)				

Deprotonation product 1t

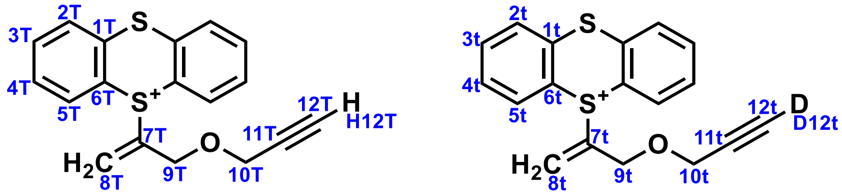

Table S5. Characterization table of 1t.

Atom	δ (ppm)	J	HSQC	HMQC	COSY	NOESY
**C1t**	137.15			3t, 5t		
**C2t**	130.44		2t			
**H2t**	7.997	d 8.0(3t), d 1.3(4t), d 0.4(5t)	2t	6t	3t	
**C3t**	135.03		3t	5t		
**H3t**	7.882	d 8.0(2t), d 7.5(4t), d 1.4(5t)	3t	1t, 5t	2t, 4t	
**C4t**	129.79		4t			
**H4t**	7.778	d 8.0(5t), d 7.5(3t), d 1.3(2t)	4t	6t	3t, 5t	5t
**C5t**	134.90		5t	3t		
**H5t**	8.248	d 8.0(4t), d 1.4(3t), d 0.4(2t)	5t	1t, 3t	4t	4t, 8tb
**C6t**	116.01			2t, 4t		
**C7t**	129.32			8ta, 8tb, 9t		
**C8t**	128.50		8ta, 8tb	9t		
**H8ta**	6.245	d 3.9(8tb), t 1.0(9t)	8t	7t, 9t	8tb, 9t	8tb, 9t
**H8tb**	5.326	d 3.9(8ta)	8t	7t, 9t	8ta	5t, 8ta

**C9t**	67.22		9t	8ta, 8tb, 10t		
**H9t**	4.133	d 1.0(8ta)	9t	7t, 8t	8ta	8ta, 10t
**C10t**	57.56		10t			
**H10t**	4.068	s	10t	9t, 11t, 12t		9t
**C11t**	77.66	(from HMQC)		10t		
**C12t**	76.91	t ~38.0 Hz(D12t; from HMQC)		10t		
**D12t**	n.d.					
**H10T**	4.069	d 2.4(12T)				
**C12T**	77.18					
**H12T**	2.920	t 2.4(10T)				

^{1}H NMR of **1e**, **1z**, **1t**

600 MHz, D_2O, 298 K

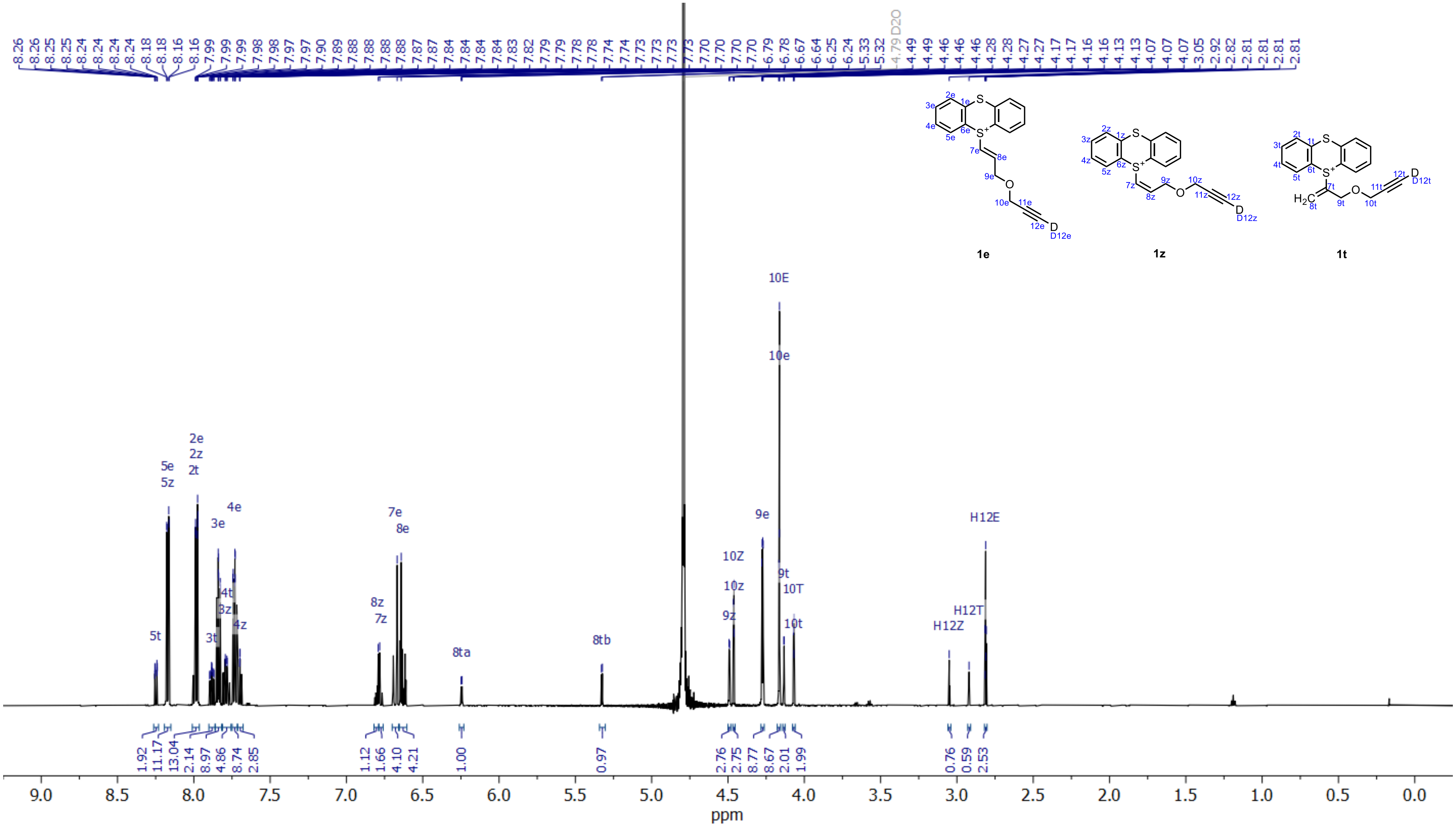

^{13}C NMR of **1e**, **1z**, **1t**

151 MHz, D_2O, 298 K

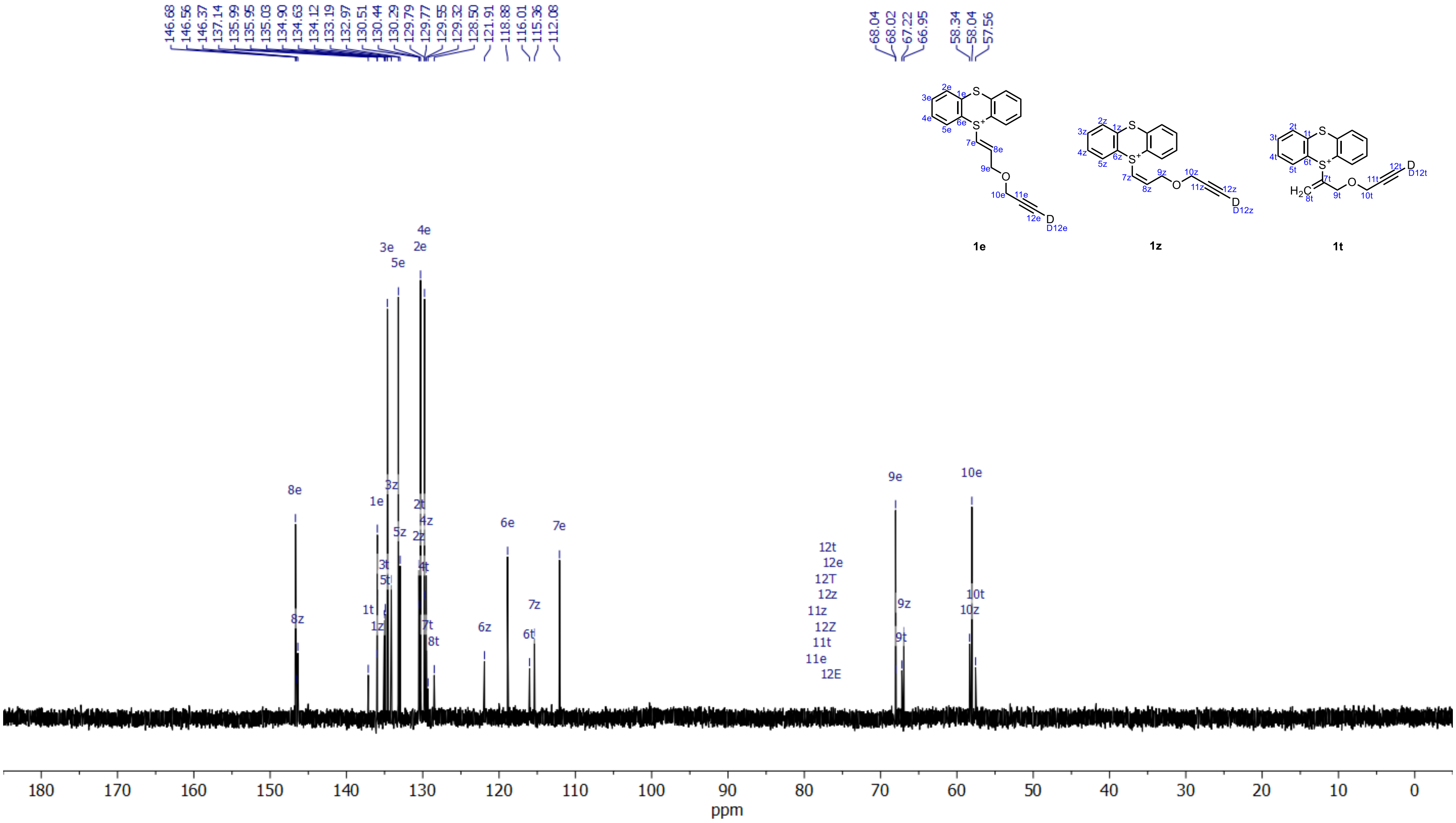

HSQC-NMR of **1e**, **1z**, **1t**

D_2O, 298 K

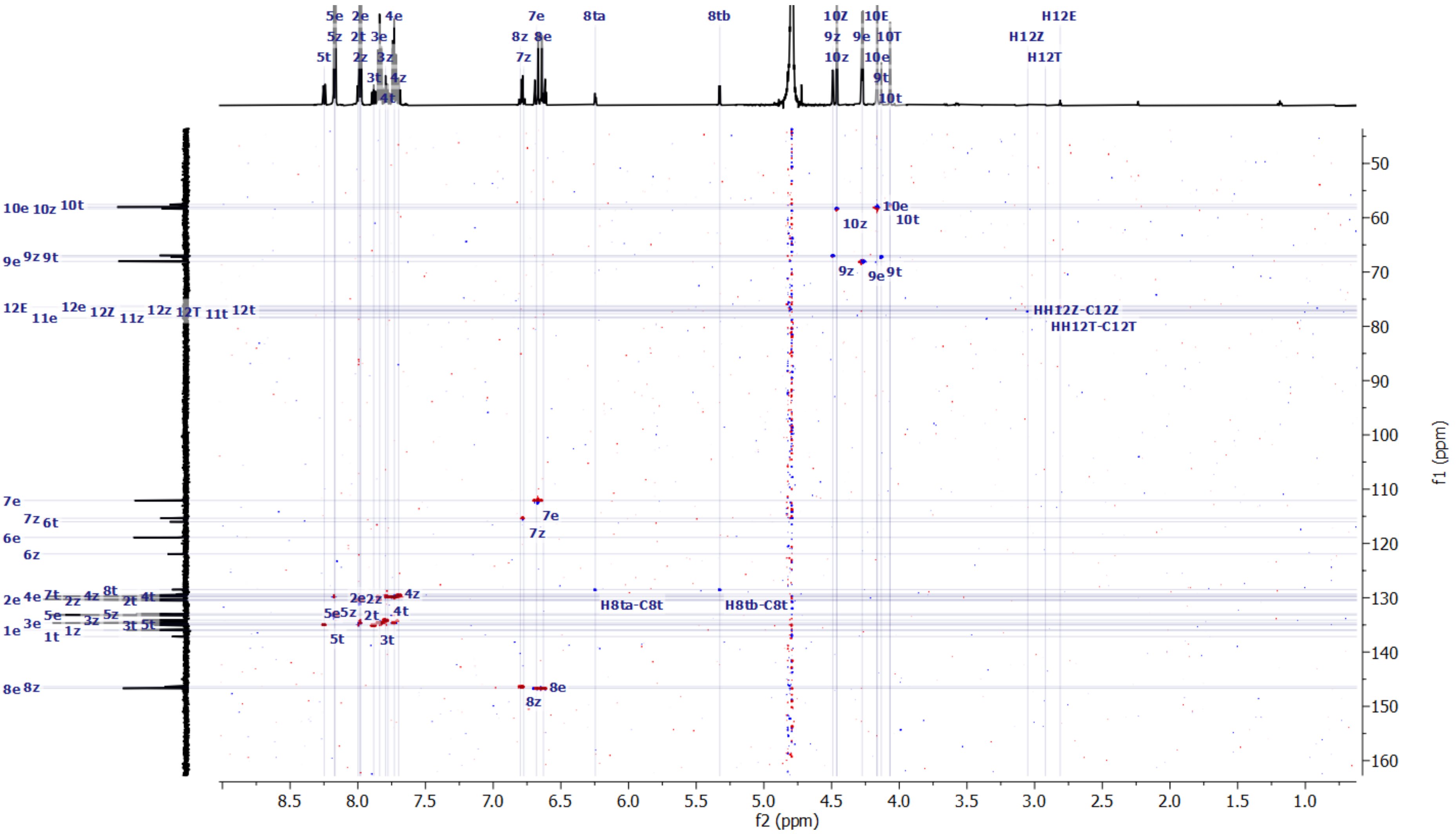

HMQC-NMR of **1e**, **1z**, **1t**

D_2O, 298 K

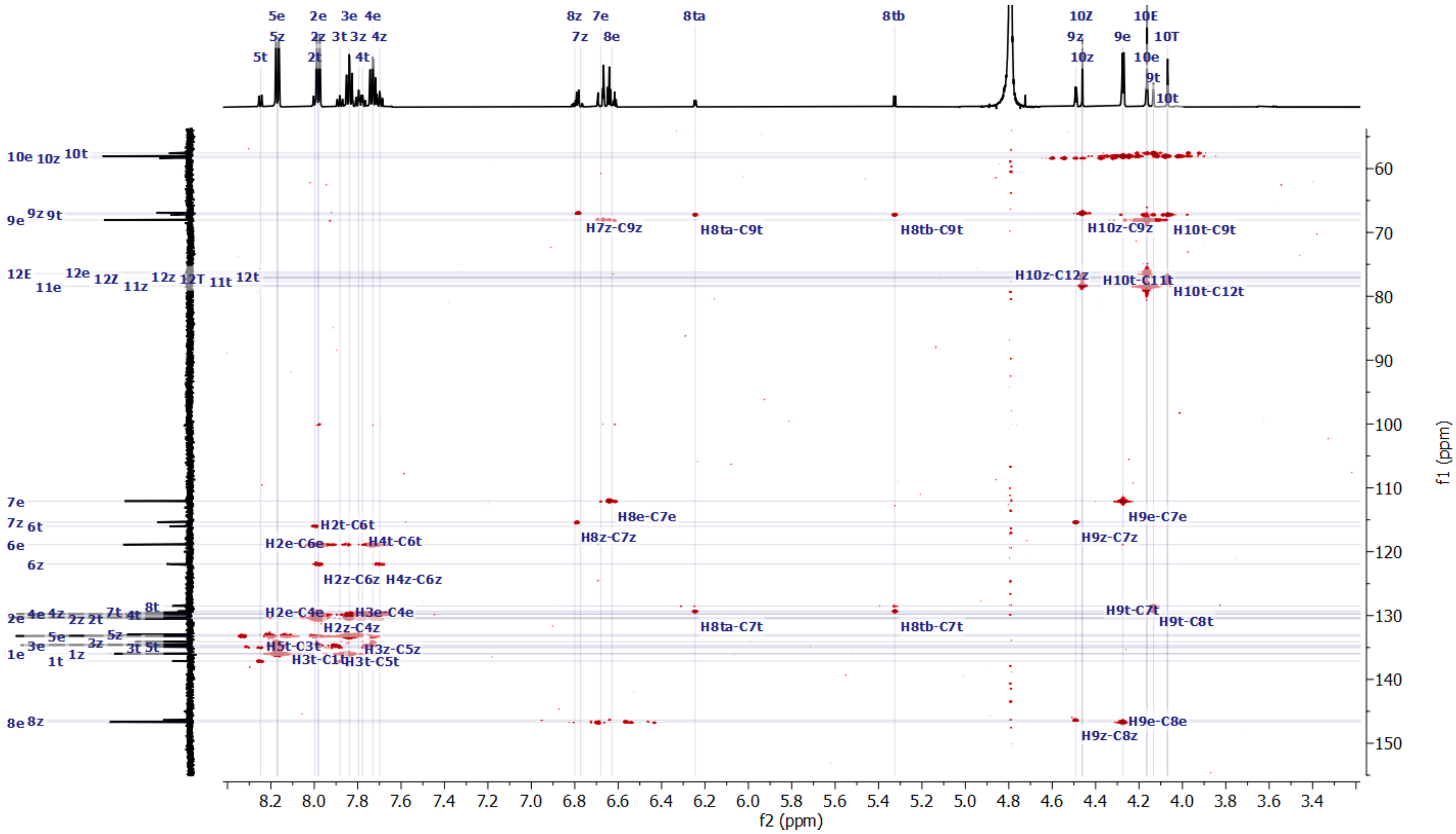

COSY-NMR of **1e**, **1z**, **1t**

D_2O, 298 K

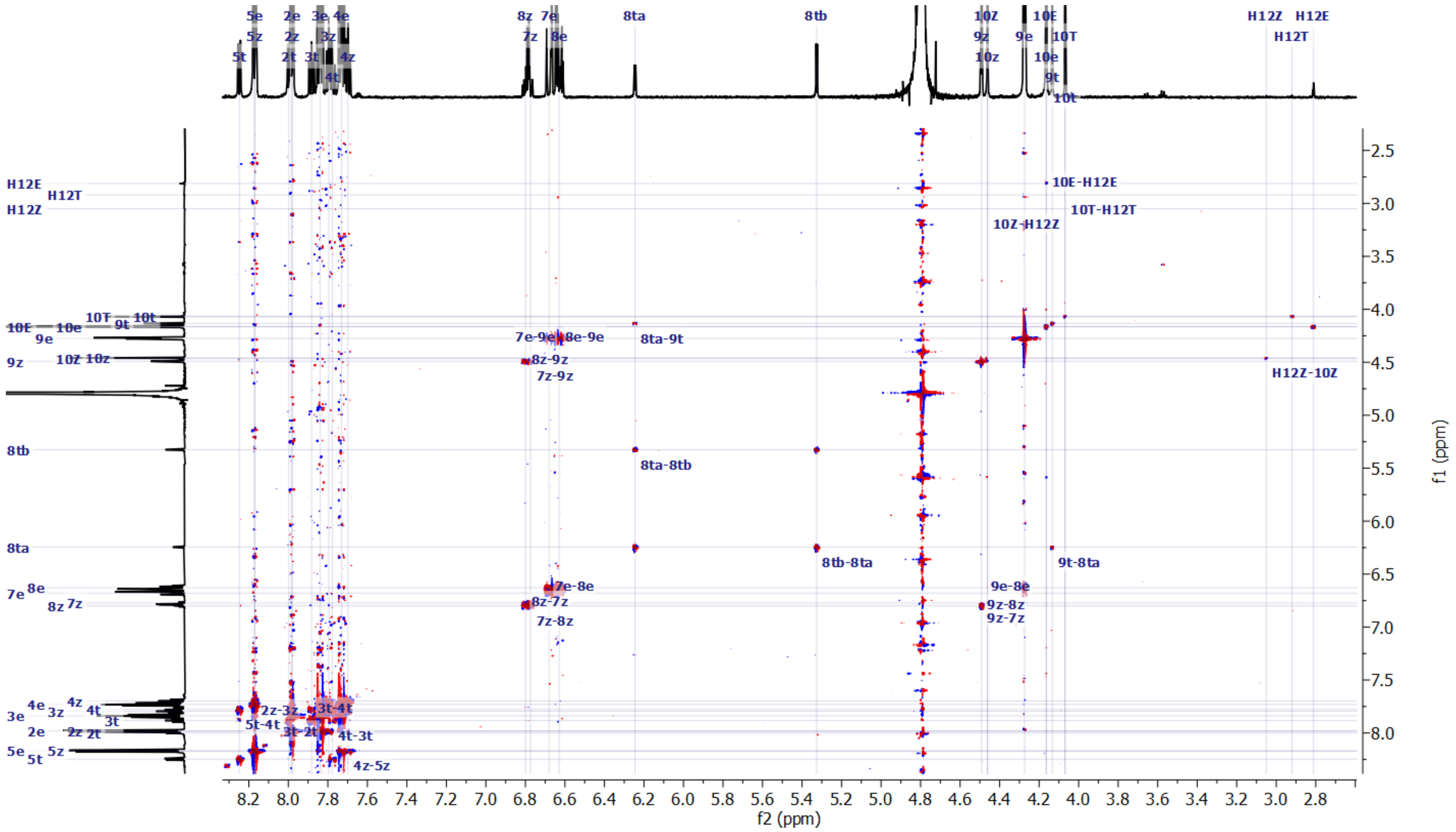

NOESY-NMR of **1e**, **1z**, **1t**

D_2O, 298 K

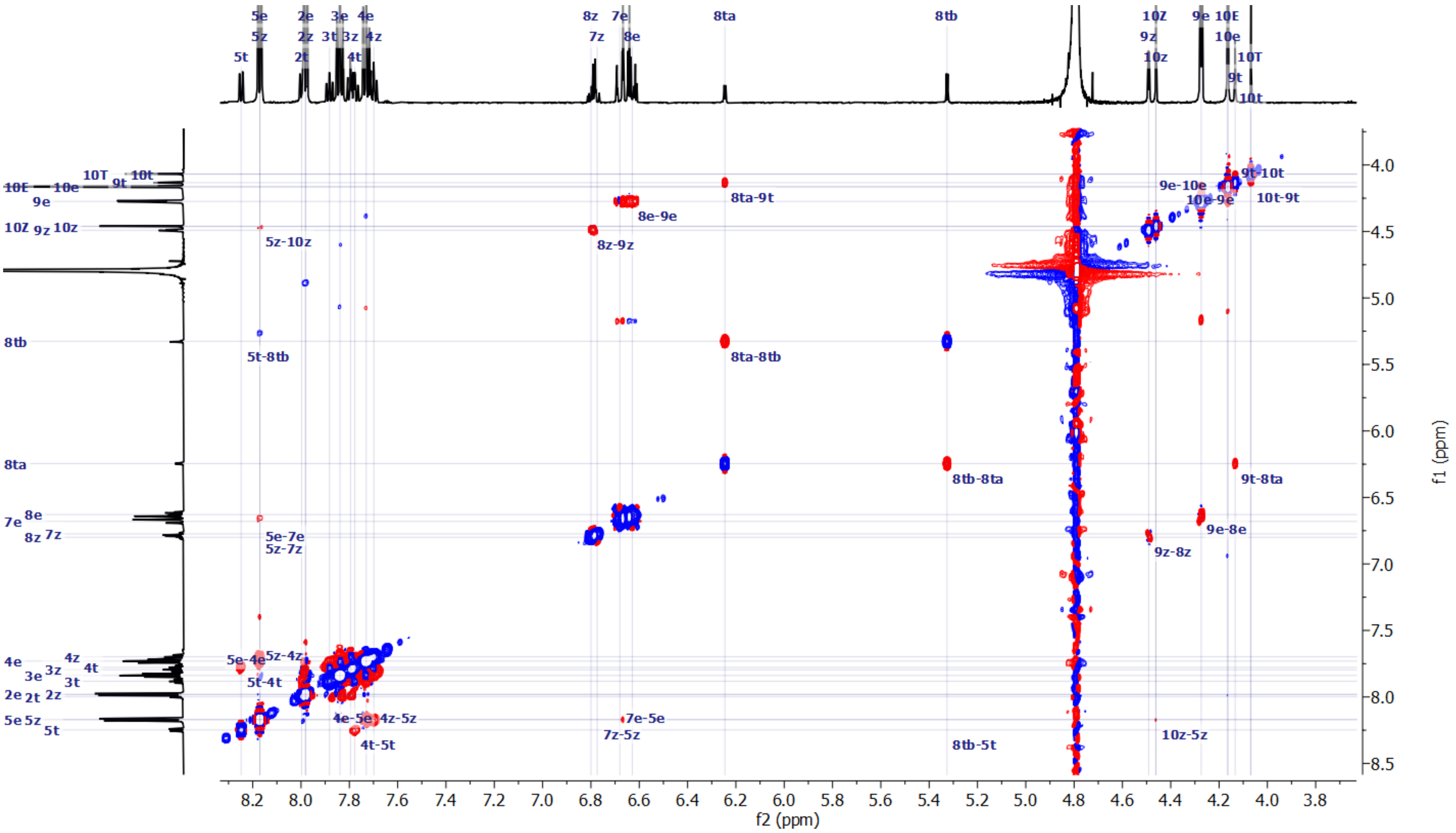

REFERENCES

1 Juliá, F., Yan, J., Paulus, F. & Ritter, T. Vinyl Thianthrenium Tetrafluoroborate: A Practical and Versatile Vinylating Reagent Made from Ethylene. *Journal of the American Chemical Society* **143**, 12992-12998, doi:10.1021/jacs.1c06632 (2021).

2 Matzinger, M. *et al.* Mimicked synthetic ribosomal protein complex for benchmarking crosslinking mass spectrometry workflows. *Nature Communications* **13**, 3975, doi:10.1038/s41467-022-31701-w (2022).

3 Bio-Rad. *A Guide to Polyacrylamide Gel Electrophoresis and Detection*.

4 Daina, A., Michielin, O. & Zoete, V. SwissADME: a free web tool to evaluate pharmacokinetics, drug-likeness and medicinal chemistry friendliness of small molecules. *Scientific Reports* **7**, 42717, doi:10.1038/srep42717 (2017).

5 Hartmann, P. *et al.* Chemoselective umpolung of thiols to episulfoniums for cysteine bioconjugation. *Nature Chemistry* **16**, 380-388, doi:10.1038/s41557-023-01388-7 (2024).

6 Hughes, C. S. *et al.* Single-pot, solid-phase-enhanced sample preparation for proteomics experiments. *Nature Protocols* **14**, 68-85, doi:10.1038/s41596-018-0082-x (2019).

7 Yan, T. *et al.* SP3-FAIMS Chemoproteomics for High-Coverage Profiling of the Human Cysteinome. *ChemBioChem* **22**, 1841-1851, doi:https://doi.org/10.1002/cbic.202000870 (2021).

8 Cox, J. & Mann, M. MaxQuant Enables High Peptide Identification Rates, Individualized p.p.b.-range Mass Accuracies And Proteome-Wide Protein Quantification. *Nature Biotechnology* **26**, 1367-1372, doi:10.1038/nbt.1511 (2008).

9 Chambers, M. C. *et al.* A Cross-platform Toolkit for Mass Spectrometry and Proteomics. *Nature Biotechnology* **30**, 918-920, doi:10.1038/nbt.2377 (2012).

10 MSFragger:. *Converting raw files to mzML*, https://fragpipe.nesvilab.org/docs/tutorial_convert.html.

11 Lenz, S. *et al.* Reliable identification of protein-protein interactions by crosslinking mass spectrometry. *Nature Communications* **12**, 3564, doi:10.1038/s41467-021-23666-z (2021).

12 Github:. *XiSearch*, https://github.com/Rappsilber-Laboratory/XiSearch.

13 Kong, A. T., Leprevost, F. V., Avtonomov, D. M., Mellacheruvu, D. & Nesvizhskii, A. I. MSFragger: ultrafast and comprehensive peptide identification in mass spectrometry–based proteomics. *Nature Methods* **14**, 513-520, doi:10.1038/nmeth.4256 (2017).

14 Mendes, M. L. *et al.* An Integrated Workflow for Crosslinking Mass Spectrometry. *Molecular Systems Biology* **15**, e8994, doi:https://doi.org/10.15252/msb.20198994 (2019).

15 Giese, S. H., Belsom, A., Sinn, L., Fischer, L. & Rappsilber, J. Noncovalently Associated Peptides Observed during Liquid Chromatography-Mass Spectrometry and Their Effect on Cross-Link Analyses. *Analytical Chemistry* **91**, 2678-2685, doi:10.1021/acs.analchem.8b04037 (2019).

16 Frankenfield, A. M., Ni, J., Ahmed, M. & Hao, L. Protein Contaminants Matter: Building Universal Protein Contaminant Libraries for DDA and DIA Proteomics. *Journal of Proteome Research* **21**, 2104-2113, doi:10.1021/acs.jproteome.2c00145 (2022).

17 Combe, C. W., Graham, M., Kolbowski, L., Fischer, L. & Rappsilber, J. xiVIEW: Visualisation of Crosslinking Mass Spectrometry Data. *Journal of Molecular Biology* **436**, 168656, doi:https://doi.org/10.1016/j.jmb.2024.168656 (2024).

18 Binns, D. *et al.* QuickGO: a web-based tool for Gene Ontology searching. *Bioinformatics* **25**, 3045-3046, doi:10.1093/bioinformatics/btp536 (2009).

19 Honorato, R. V. *et al.* The HADDOCK2.4 web server for integrative modeling of biomolecular complexes. *Nature Protocols* **19**, 3219-3241, doi:10.1038/s41596-024-01011-0 (2024).

20 Vangone, A. & Bonvin, A. M. J. J. Contacts-based prediction of binding affinity in protein–protein complexes. *eLife* **4**, e07454, doi:10.7554/eLife.07454 (2015).

21 Xue, L. C., Rodrigues, J. P., Kastritis, P. L., Bonvin, A. M. & Vangone, A. PRODIGY: a web server for predicting the binding affinity of protein-protein complexes. *Bioinformatics* **32**, 3676-3678, doi:10.1093/bioinformatics/btw514 (2016).

22 Perez-Riverol, Y. *et al.* The PRIDE database at 20 years: 2025 update. *Nucleic Acids Research* **53**, D543-D553, doi:10.1093/nar/gkae1011 (2024).

23 Perez-Riverol, Y. *et al.* The PRIDE database resources in 2022: a hub for mass spectrometry-based proteomics evidences. *Nucleic Acids Research* **50**, D543-D552, doi:10.1093/nar/gkab1038 (2021).
